# Vacancy Engineered Zero‐Valent Iron Steer Hydrogen Spillover toward Per‐ and Polychlorinated Organics Rapid Complete Dechlorination

**DOI:** 10.1002/advs.202512668

**Published:** 2025-09-02

**Authors:** Zimin Yan, Bin Wu, Zhiling Li, Tianyi Huang, Di Cao, Yunxia Zu, Shih‐Hsin Ho, Aijie Wang

**Affiliations:** ^1^ State Key Laboratory of Urban‐rural Water Resource and Environment School of Environment Harbin Institute of Technology Harbin 150090 P. R. China; ^2^ State Key Laboratory of Urban‐rural Water Resource and Environment School of Civil and Environmental Engineering Harbin Institute of Technology Shenzhen Shenzhen 518055 P. R. China

**Keywords:** carbon vacancy, hydrogen spillover, per‐ and polychlorinated organics, rapid and complete dichlorination, zero‐valent iron

## Abstract

The elevated toxicity and persistent bioaccumulative propensity of per‐ and polychlorinated organics (PCOs) pose a substantial environmental hazard; however, current dechlorination technologies encounter challenges in surmounting the cumulative reductive inertia inherent to PCOs, resulting in low dechlorination efficiency and the persistence of ecotoxicity. Here, a vacancy‐engineered zero‐valent iron (ZVI) is proposed to address this challenge. The surface‐modified carbon vacancies can extract outward‐flowing electrons from lattice copper‐doped ZVI (CvCu‐ZVI), which react with trapped protons to generate reactive hydrogen in situ that subsequently spills over onto ZVI. Reversible hydrogen spillover enhances electron transfer, and superhydrophobic modification leads to a 38.1‐fold increase in unit site activity and up to 95% electron utilization in CvCu‐ZVI. Excitingly, this approach achieves carbon tetrachloride complete dechlorination, a typical PCOs, with a record intrinsic activity of 13.7 h^−1^, outperforming state‐of‐the‐art ZVI‐based reductants. Theoretical calculations reveal that modulated hydrogen spillover substantially reduces the dechlorination energy barrier of low‐chlorinated intermediates, facilitating C─Cl bond dissociation. Furthermore, this novel ZVI has a lower production cost and environmental impact, and can be integrated into permeable reaction units for long‐term efficient organohalogen pollution remediation in natural groundwater. This broadly applicable approach establishes a promising paradigm for the sustainable remediation of PCOs pollution.

## Graphical abstract

Zero‐valent iron with a vacancy‐rich carbon shell and lattice Cu doping in Fe^0^ cores generates a dechlorination mechanism combining enhanced electron transfer and modulated H^*^ spillover, which significantly lowers the energy barrier for dechlorinating low‐chlorinated intermediates, accelerates C─Cl bond dissociation, and then delivers breakthrough performance in the rapid and complete dechlorination of carbon tetrachloride.

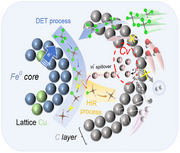

## 1 Introduction

Per‐ and polychlorinated organics (PCOs) are ubiquitous in groundwater, where they pose considerable threats to ecological safety and human health.^[^ ^1^, ^2^ ^]^ Chemical reduction can effectively break C─Cl bonds to dechlorinate PCOs.^[^ ^3^ ^]^ However, for PCOs with low electron affinity, such as carbon tetrachloride (CCl_4_), the energy barrier required for dechlorination is high.^[^ ^4^ ^]^ Meanwhile, hydrogenation and a decrease in the number of chlorine substituents usually lead to structural saturation and a large decrease in electron affinity for the dechlorination intermediates, resulting in incomplete dechlorination and the accumulation of toxic intermediates.^[^ ^5^ ^]^ Despite numerous reports on dechlorination approaches,^[^ ^6^, ^7^, ^8^ ^]^ achieving rapid and complete dechlorination of PCOs remains a challenge.

Zero‐valent iron (Fe^0^, ZVI) is highly promising for dechlorination. ZVI is characterized by a unique core–shell structure with an electron‐rich Fe° core and a surface iron oxide (FeO*_x_*) layer.^[^ ^9^ ^]^ Because of its hydrophilicity, ZVI is less selective for hydrophobic pollutants than for water, which results in loss of electrons.^[^ ^10^ ^]^ To address this dilemma, ZVI modified with hydrophobic non‐metallic elements such as nitrogen, phosphorus, and sulfur has been synthesized as an alternative. This modified ZVI increases the electron utilization rate and enhances the dechlorination of various PCOs by several orders of magnitude.^[^ ^6^, ^11^, ^12^, ^13^ ^]^ Notably, this hydrophobization modification inevitably inhibits hydrogen adsorption and the generation of active hydrogen (H*), which leads to ZVI's reduction ability becoming reliant on the direct electron transfer.^[^ ^6^, ^14^ ^]^ However, the substantial reduction in electron affinity of dechlorination intermediates often leaves the direct electron transfer (DET) mechanism with an insufficient driving force for deep dechlorination.^[^ ^4^ ^]^ For instance, sulfonated ZVI fails to proceed with dechlorination after converting CCl_4_ to chloroform (CHCl_3_) or trace dichloromethane (CH_2_Cl_2_).^[^ ^8^, ^15^, ^16^ ^]^ The H^*^, which has a high reduction potential (−2.3 V vs the standard hydrogen electrode), can possibly act as an additional driving force to promote deep dechlorination.^[^ ^6^, ^8^, ^15^, ^16^, ^17^ ^]^ Therefore, promoting the formation of H^*^ and inhibiting its recombination to H_2_ (resulting in the loss of electrons from ZVI) without site competition with the substrate is essential for the deep dechlorination of PCOs.

As an inherent ionic defect, vacancies are rich in local electrons that can attract protons and be reduced to form H^*^.^[^ ^18^, ^19^ ^]^ During photocatalysis, the hollow sites can act as electron traps to trap excited photogenerated electrons for reduction reactions.^[^ ^20^ ^]^ The carbon structure can reportedly stabilize and facilitate the transfer of H^*^.^[^ ^21^ ^]^ Therefore, the strategic addition of carbon vacancies (Cv) can be used to design a hydrogen spillover pathway and transfer the in situ generated H^*^ from Cv to the adjacent Fe^0^ site to participate in dechlorination. Furthermore, the transition metal copper (Cu, d10) is utilized as a Lewis acid for lattice tuning to promote electron release from the iron nucleus, thus ensuring the continued production of H^*^ on Cv.

**Figure 1:**
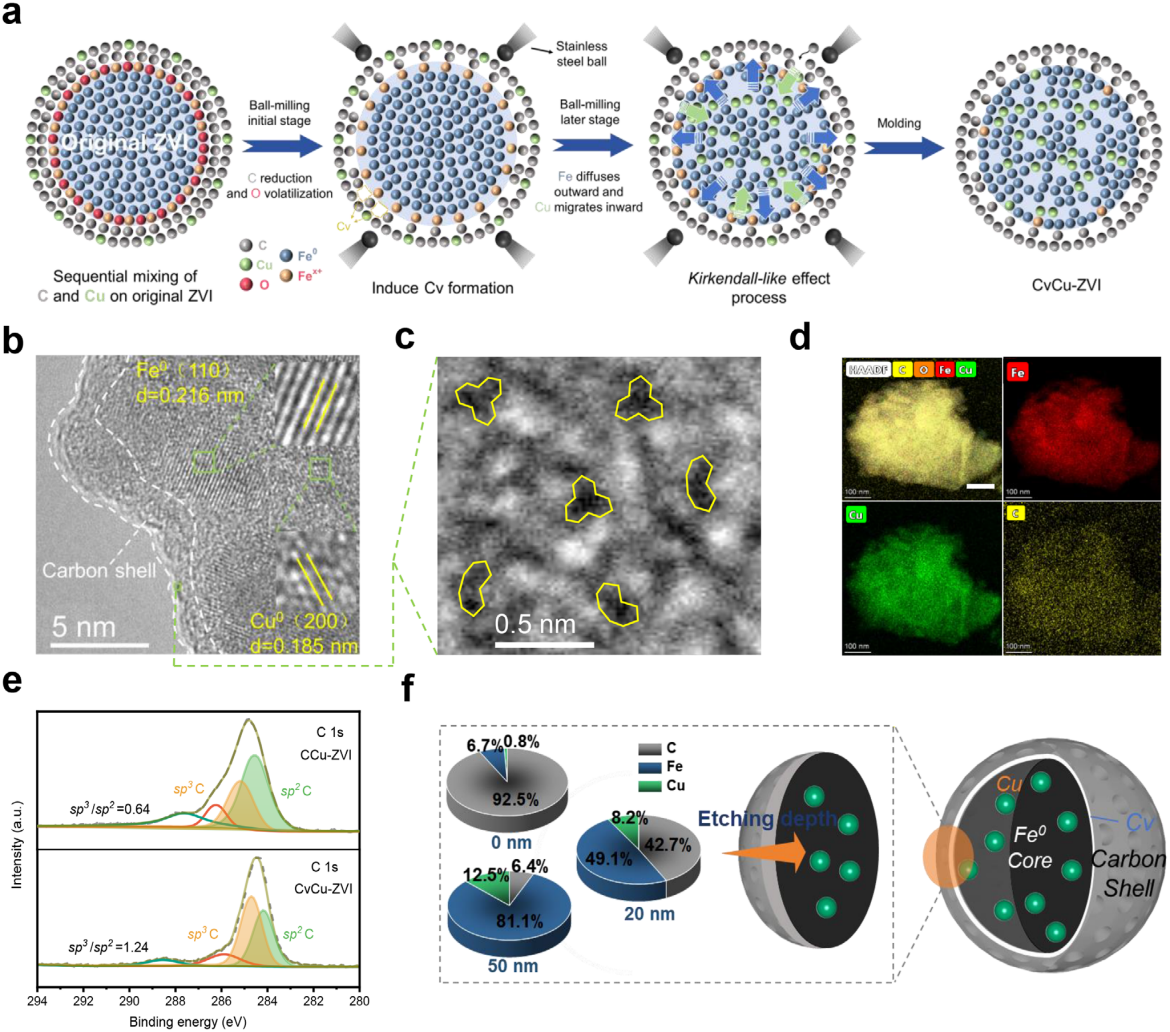
a) The formation process of Cv and lattice Cu co‐modified ZVI (CvCu‐ZVI). b) High‐resolution transmission electron microscopy and c) aberration‐corrected high‐angle annular dark‐field transmission electron microscopy images and d) the corresponding elemental mapping of CvCu‐ZVI. e) High‐resolution XPS C 1s spectra of CCu‐ZVI and CvCu‐ZVI. f) Content analysis of the elements in the XPS depth profile and the structure of CvCu‐ZVI consisted of Cv‐containing carbon shells and a lattice Cu‐doped Fe° core.

Here, we envisioned the FeO*_x_* layer as a sacrificial template to promote oxygen volatilization and carbon reduction to form Cv under high temperature and stress during ball milling, and simultaneously facilitate the lattice impregnation of Cu (**Figure** **1**a). The objective was to use the Cv and electrons extracted from ZVI to convert the captured proton to H^*^ in situ and facilitate hydrogen spillover to the dechlorination site using a concentration difference. The H^*^ will provide a driving force for the dechlorination of PCOs. The tunable physicochemical properties, enhanced electron transfer, and reversible hydrogen spillover significantly improve electron utilisation efficiency and greatly reduce the dechlorination energy barrier of the intermediate, resulting in complete dechlorination of CCl_4_ and selective hydrogenation of the product. The material's intrinsic activity should exceed that of all reported advanced dechlorination approaches. In addition, the stability of Cv and lattice‐Cu co‐modified ZVI (CvCu‐ZVI) was investigated along with the scale‐up cost and environmental impact of the synthesis and application.

## 2 Results and Discussion

### 2.1 Materials Synthesis and Characterization

ZVI with various structural modifications was synthesized using top‐down mechanical ball milling (Figure 1a; Figure S1a, Supporting Information). Scanning electron microscopy images (Figure S1b–e, Supporting Information) revealed that Cu doping induced fragmentation of ZVI and roughened its surface. A carbon shell covered the surface of ZVI with its porous and folded honeycomb structure. The encapsulation of the carbon shell gives ZVI a larger specific surface area and mesoporous structure, which is conducive to improving the mass transfer efficiency (Figure S2 and Table S1, Supporting Information). Slight displacement of the Fe (110) peak and an increase in the interplanar spacing in Cu‐ZVI compared with ZVI suggested that Cu was doped into the Fe^0^ body‐centered‐cubic structure (Figure S3, Supporting Information), which induced tensile strain in the lattice.^[^ ^6^, ^9^ ^]^ However, the Fe^0^ lattice structure remained unchanged in Cv‐ZVI, which indicated that the carbon was distributed on the ZVI surface. High‐resolution transmission electron microscopy showed a lattice continuity break corresponding to the Fe (110) facet, which may correspond to asymmetric displacement between Fe and Cu (Figure 1b; Figure S4, Supporting Information).^[^ ^22^ ^]^ The energy dispersive X‐ray spectroscopy analysis revealed Fe and Cu were uniformly distributed within CvCu‐ZVI, whereas carbon was predominantly located on the surface (Figure 1d; Figure S5 and Table S2, Supporting Information). Aberration‐corrected high‐angle annular dark‐field transmission electron microscopy revealed that the surface of CvCu‐ZVI was rich in topological carbon defects (Figure 1c).^[^ ^18^ ^]^

To verify the role of FeO*_x_* layers in the formation of Cv, carbon defect‐free ZVI (CCu‐ZVI) for use as a control was prepared in parallel using pre‐acidified iron powder ball‐milled under the same conditions. The carbon defects in various ZVI‐based materials were characterized using X‐ray photoelectron spectroscopy (XPS) and Raman spectroscopy (Figure 1e; Figures S6 and S7, Supporting Information). Specifically, the sp^3^/sp^2^ ratio derived from C1s XPS spectra could reflect the extent of defects in materials.^[^ ^17^, ^23^ ^]^ The sp^3^/sp^2^ value of CvCu‐ZVI (1.24) was much larger than that of CCu‐ZVI (0.64) (Figure 1e). Raman spectra (Figure S8, Supporting Information) clearly showed characteristic D and G peaks at 1358 cm^−1^ for disordered carbon and 1593 cm^−1^ for sp^2^‐hybridized graphitic carbon.^[^ ^24^ ^]^ The intensity ratios of the D and G peaks (*I*_D_/*I*_G_ value) were in the following order: CvCu‐ZVI (0.89) > Cv‐ZVI (0.85) > CCu‐ZVI (0.72). Additionally, in electron paramagnetic resonance (EPR) spectroscopy (Figure S9, Supporting Information), the peak for CCu‐ZVI was minimal, whereas the peaks for Cv‐ZVI and CvCu‐ZVI were large, which showed that Cv was present in the latter samples.^[^ ^25^ ^]^ These results demonstrate that the FeO*_x_* layer is crucial for the formation of Cv.

Because the generation of Cv is associated with the thermal sublimation of the FeO*_x_* layer, thermogravimetric mass spectrometry was used to trace the formation of Cv. The signal intensity of CO (*m/z* = 28, appropriate temperature) and CO_2_ (*m/z* = 44, appropriate temperature) released from CvCu‐ZVI increased with decreases in the ball milling time (Figure S10, Supporting Information). This change indicated that sustained thermal reduction of carbon occurred, and this corresponded to the formation of Cv. XPS was used to monitor the process of Cv formation. As the ball milling time increased from 0 to 3 h, the O═C─O bond content initially increased and then decreased, whereas the Fe─O bond content decreased continually (Figures S11–S13, Supporting Information). These results showed that thermal reduction and oxygen loss occurred after the bonding of carbon with the O atoms of FeO*_x_* during ball milling. The accumulated evidence confirmed that, under the elevated temperature and stress compression of ball milling, cleavage of Fe─O bonds led to loss of O atoms, which induced the formation of Cv. XPS analysis of the depth profile of CvCu‐ZVI was performed to determine the chemical distribution of the material (Figure 1f; Figure S14, Supporting Information). Almost no Cu signal was detected on the surface of CvCu‐ZVI, which was consistent with the absence of Cu on the surface of the material (Figure 1d) and further confirmed that Cu was doped in the lattice in the Fe° core rather than deposited on the surface. The depth distribution of C signal in the material showed the opposite trend to that of Cu signal. Therefore, CvCu‐ZVI consisted of a lattice Cu‐doped Fe° core and Cv‐containing carbon shells (Figure 1f).

### 2.2 Atomic‐Level Structure and Tunable Physicochemical Properties

**Figure 2:**
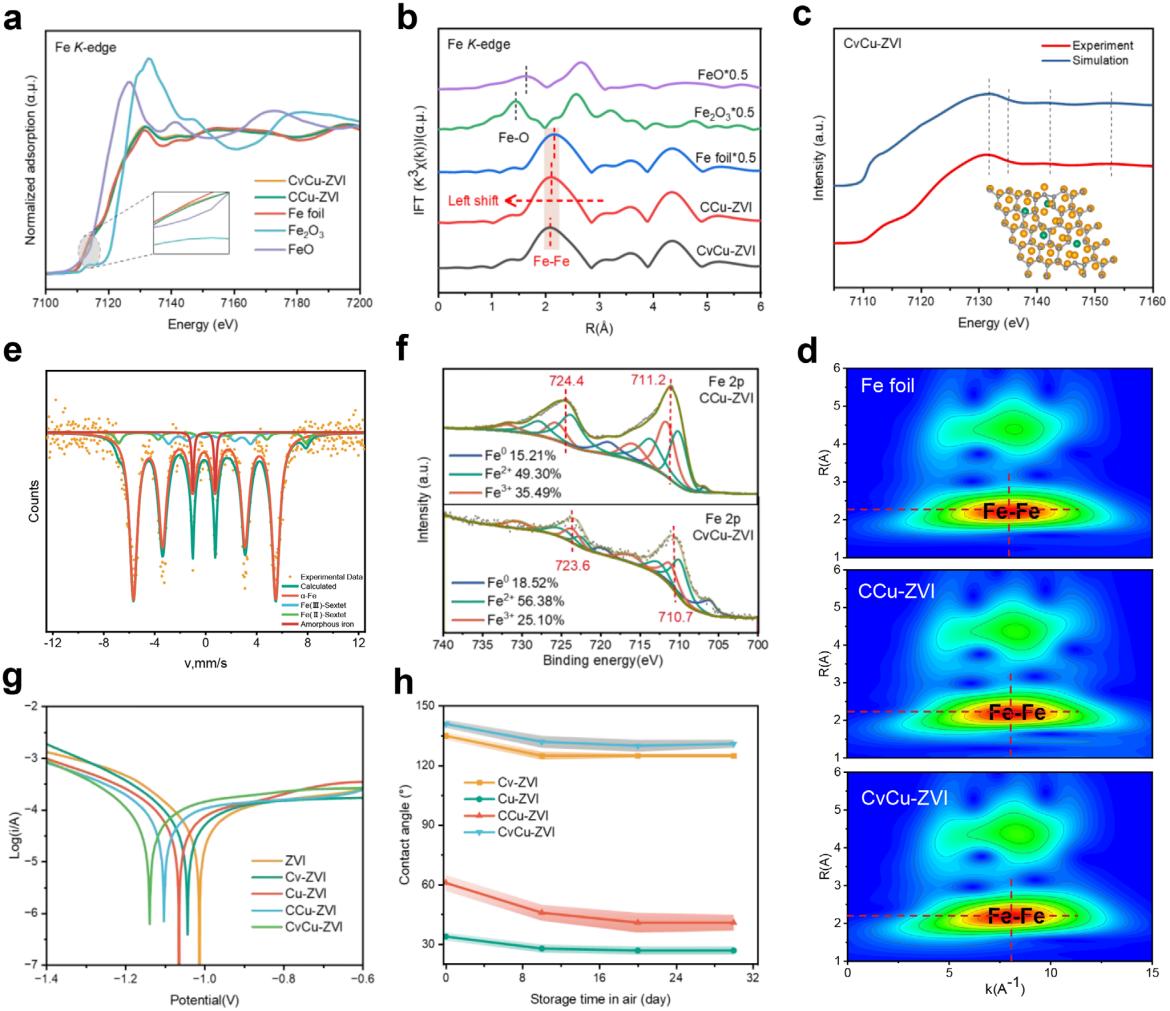
a) Fe *K*‐edge XANES spectra and b) corresponding FT‐EXAFS results in R space of CvCu‐ZVI, CCu‐ZVI, Fe foil, FeO, and Fe_2_O_3_. c) Simulated XANES spectra derived by finite‐difference near‐edge structure simulation for CvCu‐ZVI. d) Wavelet transform plots of CvCu‐ZVI, CCu‐ZVI, and Fe foil. e) ^57^Fe Mössbauer spectroscopy spectra of CvCu‐ZVI. (f) XPS Fe 2p spectra of CCu‐ZVI and CvCu‐ZVI. g) Tafel polarization curves of different materials. (h) WCA measurements of different materials over a 30‐day period.

The chemical state and coordination environment of Fe atoms were investigated using X‐ray absorption near‐edge structure (XANES) and extended X‐ray absorption fine structure (EXAFS) measurements.^[^ ^26^, ^27^, ^28^ ^]^ The XANES spectra (**Figure** **2**a) showed that the absorption edge energy of the Fe *K*‐edge in CvCu‐ZVI and CCu‐ZVI was similar to that of Fe foil, which indicated that the oxidation state of Fe atoms was close to zero.^[^ ^11^ ^]^ The EXAFS spectra were further analyzed using Fourier transform *k* ^3^‐weighted χ(k) analysis to determine the atomic configuration of iron in the materials. The dominant peaks associated with the Fe–Fe shell appeared at approximately 2.2 Å in the R‐space for both CvCu‐ZVI and CCu‐ZVI (Figure 2b). Quantitative structural parameters for iron sites were obtained through least‐squares fitting of the EXAFS and Fourier transform *k* ^3^‐weighted χ(k) EXAFS data (Figure S15 and Table S4, Supporting Information). The coordination number of Fe–Fe in CCu‐ZVI was 4.4, and that in CvCu‐ZVI was 4.2. By comparison, Fe foil has a coordination number of eight.^[^ ^13^ ^]^ This decrease in the coordination number is consistent with the reduction in the Fe–Fe shell intensity and implies that Cv and lattice Cu weaken Fe–Fe interactions, which will enhance electron release and reactivity.^[^ ^29^ ^]^ A finite‐difference near‐edge structure simulation of CvCu‐ZVI was conducted and validated against experimental XANES data (Figure 2c). The simulation results closely matched the experimental spectra, which confirmed the accuracy of the predicted configuration. Wavelet transform analysis was also conducted to examine the Fe *K*‐edge EXAFS oscillations in the samples. The wavelet transform maximum was observed at ≈8.1 Å^−1^ in both CvCu‐ZVI and CCu‐ZVI, which was consistent with the Fe–Fe structure present in the Fe foil (Figure 2d). Therefore, modification of the lattice‐Cu and Cv did not alter the dominant position of Fe^0^ in the structure but adjusted the local coordination and geometric configuration. To efficiently differentiate among the various Fe species in CvCu‐ZVI, we used ^57^Fe Mössbauer spectroscopy. CvCu‐ZVI displayed four fitted dipoles for D_1_ (α‐Fe, Fe^0^), D_2_ (Fe^2+^), D_3_ (Fe^3+^), and D_4_ (amorphous Fe^0^), with isomeric shifts of less than two and abundances of 85.0%, 4.8%, 6.2%, and 4.0%, respectively (Figure 2e; Table S5, Supporting Information). This demonstrated that the Fe species in CvCu‐ZVI were predominantly in the zero‐valent state, which was consistent with the XANES results (Figure 2a,b). Conventional XPS and Auger electron spectroscopy were used to characterize the surface chemical composition and state of the material. The results show that both Cv and lattice‐Cu synergistically induced Fe^0^ regeneration and iron reduction on CvCu‐ZVI surface (Figure 2f; Figures S16 and S17 and Table S1, Supporting Information).

Although *d*‐orbital hybridization by Cu can facilitate lowering of the *d*‐band center and band gap of ZVI to accelerate electron transfer,^[^ ^30^ ^]^ this may enhance the hydrophilicity of the material and reduce its reaction selectivity to PCOs. By contrast, carbon shells can provide hydrophobic protection and electrical conductivity to materials.^[^ ^18^, ^23^ ^]^ Thus, we investigated the synergistic influence of Cv and lattice Cu on the physicochemical properties of ZVI. Changes in the open‐circuit potential (OCP) of the different materials at the electrode were first measured to evaluate their susceptibility to spontaneous corrosion in water (Figure S18a, Supporting Information). As expected, CvCu‐ZVI exhibited the most positive and stable OCP, which suggested that the coexistence of Cv and lattice Cu synergistically enhanced the passivation resistance of ZVI. The corrosion rates and electron transfer efficiencies of the materials were determined from the Tafel polarization curves. The corrosion current magnitudes of the materials were in the following order: CvCu‐ZVI > CCu‐ZVI > Cu‐ZVI > Cv‐ZVI > ZVI (Figure 2g). The corrosion potentials showed the opposite trend, which indicated that Cv and lattice Cu enabled ZVI to exhibit stronger electron release and faster electron transfer.^[^ ^22^, ^31^ ^]^ Additionally, the relative radius of the Nyquist circle observed in the electrochemical impedance spectroscopy (Figure S18b, Supporting Information) matched well with the trend of the Tafel polarization curves, and CvCu‐ZVI had the lowest impedance and charge transfer resistance, which is beneficial to the reactivity of the material.^[^ ^32^ ^]^ Notably, the above electrochemical tests showed that the incorporation of lattice Cu into the Fe° cell more significantly promoted the corrosion tendency and electron transfer of Fe compared to the encapsulation of carbon shells on the surface. Combining these benefits with the hydrophobicity of the carbon shells could effectively prevent ZVI from reacting in contact with water, but rather promote proton reduction to H^*^ to improve reactivity to pollutants.

Increasing the hydrophobicity of ZVI particles and inhibiting their direct contact with water can make the material more stable in air and water, facilitating transport and storage.^[^ ^13^ ^]^ This was evaluated by measuring water contact angle (WCA), as shown in Figure 2h and Figure S19 (Supporting Information). As expected, compared with pristine Fe^0^ (WCA < 40°), lattice Cu resulted in enhanced hydrophilicity of Cu‐ZVI. This is because lattice‐Cu can promote Fe° corrosion, as mentioned above), forming more hydrophilic iron (hydrated) oxides on the particle surface.^[^ ^33^ ^]^ Surprisingly, the presence of Cv significantly increased the hydrophobicity of CvCu‐ZVI compared to carbon shell‐encapsulated CCu‐ZVI without vacancies (WCA: 141° ± 2° vs 61° ± 4°), which suggested that the formation of Cv facilitated the improvement of ZVI's hydrophobicity. CvCu‐ZVI exhibited superhydrophobic properties, and water droplets are repelled by particles made of this material, with the measured WCA remaining almost constant even after several wetting attempts. This result suggests that the synergistic modification of ZVI by lattice‐Cu and Cv provides good stability and facilitates the transport, storage, and reaction selectivity of the material.

### 2.3 Evaluation of the Reductive Dechlorination Performance

**Figure 3:**
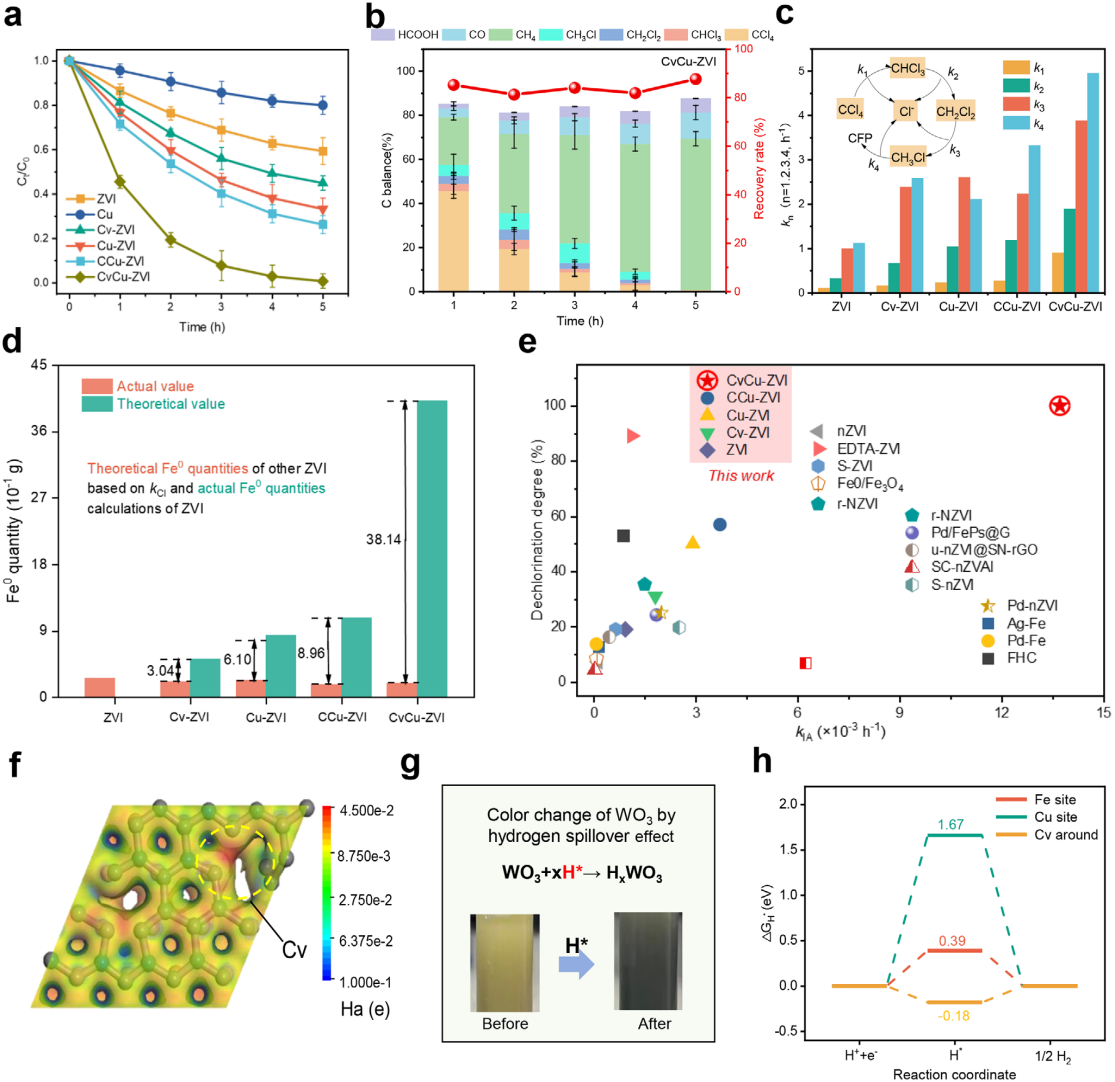
a) The efficiency of different materials for CCl_4_ removal. b) Product analysis of CvCu‐ZVI for CCl_4_ removal. c) Apparent rate constants of *k*_1_, *k*_2_, *k*_3_, and *k*_4_ for CCl_4_ removal by different materials. (Reaction conditions: [Materials]_0_ = 0.30 g L^−1^, [CCl_4_]_0_ = 5.0 mg L^−1^, pH = 7.0, T = 298K, Error bars indicate standard deviations obtained from thrice independent measurements). d) The theoretical and actual quantities of Fe^0^ required for different materials to degrade CCl_4_. e) Comparison of the published intrinsic activity rate constants (*k*_IA_) and dechlorination degree of Fe‐based materials for CCl_4_ removal (The related information was presented in Table S7, Supporting Information). f) Electrostatic potential map of CvCu‐ZVI. g) Color change of WO_3_ on CvCu‐ZVI by hydrogen spillover effect. h) Gibbs free energy diagram for hydrogen adsorption on CvCu‐ZVI.

CCl_4_, which is a typical per‐ and polychlorinated organic compound with low electron affinity (Figure S20 and Table S6, Supporting Information), was chosen to evaluate the performance of CvCu‐ZVI. Pristine ZVI exhibited a low CCl_4_ reduction efficiency of 40.63% (**Figure** **3**a). By contrast, ZVI with Cv or lattice Cu modifications had a higher reduction efficiency. With CvCu‐ZVI, the CCl_4_ reduction efficiency reached 100% within 5 h. The apparent rate constant (*k*_1_) of CvCu‐ZVI for CCl_4_ conversion was 0.91 h^−1^, which was notably higher than the conversion rates of CCu‐ZVI and other materials (Figure S21, Supporting Information). The contents of Cv and lattice Cu were crucial for the reductive dechlorination of CCl_4_ (Figures S22 and S23, Supporting Information). Importantly, CvCu‐ZVI maintained high activity after six consecutive reduction cycles (Figure S24, Supporting Information), which indicated it was stable.

Considering that the reduction of CCl_4_ only represents its conversion to CHCl_3_ (*k*_1_) and does not reflect the total dechlorination, we determined the content of intermediates, dechlorination rate, and dynamic mass balance of chlorine during the reaction (Figure 3b,c).^[^ ^34^ ^]^ The reductive dechlorination of CCl_4_ occurred as follows: CCl_4_ → CHCl_3_ → CH_2_Cl_2_ → monochloromethane (CH_3_Cl) → chlorine‐free products (Figure 3c; Figure S25, Supporting Information). Among them, only CvCu‐ZVI was able to completely dechlorinate CCl_4_ to chlorine‐free products, including methane (≈71.26%), carbon monoxide (≈12.0%), and formic acid (≈5.80%) (Figure 3b; Figure S26, Supporting Information). The ultra‐high dechlorination performance of CvCu‐ZVI was further confirmed by the release of chloride ions (Figure S27, Supporting Information). For the detected intermediate CHCl_3_, the apparent rate constant (*k*_2_) for CvCu‐ZVI was 1.90 h^−1^, which was 5.76‐, 2.84‐, 1.80‐, and 1.60‐fold the rate constants of ZVI, Cv‐ZVI, Cu‐ZVI, and CCu‐ZVI, respectively. Additionally, for CH_2_Cl_2_ and CH_3_Cl, CvCu‐ZVI had apparent rate constants *k*_3_ and *k*_4_ of 3.89 and 4.96 h^−1^, respectively, which were much larger than those of the other materials (Figure 3c; Figure S28, Supporting Information). This result suggested that the underlying reason for the high dechlorination activity of CvCu‐ZVI against CCl_4_ was the facilitated reductive conversion of the lower chlorine intermediates (CH_2_Cl_2_ and CH_3_Cl). In fact, previous studies reported that H^*^ may have higher reactivity selectivity for low chlorine intermediates,^[^ ^15^, ^16^, ^17^ ^]^ we thus speculate that CvCu‐ZVI might overcome the dechlorination inertia via H^*^ reduction to achieve CCl_4_ complete dechlorination.

However, the actual quantities of Fe^0^ showed no correlation with the observed apparent rate of dechlorination (*k*_Cl_) (Figure S29, Supporting Information). For this reason, further analysis was performed to understand the high performance of CvCu‐ZVI. Assuming equal activity of unit Fe^0^ sites in each material, the theoretically required Fe^0^ quantity for CvCu‐ZVI, based on the dechlorination activity of ZVI for CCl_4_ and its Fe^0^ content, was calculated to be 4.02 g (Figure 3d; Figure S30, Supporting Information). This value was significantly higher than the actual Fe^0^ loading of CvCu‐ZVI (0.20 g). In other words, the per unit Fe^0^ activity of CvCu‐ZVI increased by 38.14 times. The ZVI intrinsic activity rate constant (*k*_IA_) was calculated by normalizing the observed apparent rate of dechlorination (*k*_Cl_) by the Fe^0^ quantity. The *k*_IA_ of CvCu‐ZVI (13.70 h^−1^) was 3.7–14.94 times that of any other material, which showed it had superior intrinsic chemical‐reducing properties (Figure 3e; Figure S31 and Tables S7 and S8, Supporting Information). These results are the highest level of dechlorination (100%) and the highest intrinsic activity rate constant for removal of CCl_4_ by a reduction process reported to date. We also investigated the reductive dechlorination activity of CvCu‐ZVI for several other PCOs (Figure S32, Supporting Information). The results showed that trichloroethylene and perchloroethylene were completely removed (100% dechlorination) in 4 h, with dechlorination rates of 0.84 and 0.72 h^−1^. The removal rates of 2,4,6‐trichlorophenol, 1,2,4‐trichlorobenzene, and hexachlorobutadiene were 76.49%, 88.93%, and 67.56%, respectively, after 5 h. These results demonstrated the universality of CvCu‐ZVI for the reductive dechlorination of different PCOs.

### 2.4 Hydrogen Spillover Facilitated by Carbon Vacancies

Regarding the occurrence of hydrogen spillover is closely related to proton trapping, H^*^ generation and subsequent H^*^ transfer, we have investigated the dechlorination mechanism of CvCu‐ZVI using a combination of controlled experiments and theoretical calculations. Theoretically, proton adsorption on ZVI and Cu‐ZVI was endothermic (0.30 and 0.17 eV), which rendered it thermodynamically unfavorable (Figure S33, Supporting Information). In contrast, protons were strongly adsorbed at the vacancies in the carbon shell of CvCu‐ZVI, with an adsorption energy of −1.13 eV. Electrostatic potential analysis showed that the negative potential at Cv indicated a strong capacity for proton trapping and stabilization compared to the evidently positive potentials at Fe and Cu sites (Figure 3f; Figure S34, Supporting Information). These results underscored the potential role of Cv in reducing the trapped proton to H^*^.

Generally, the anodic Tafel slope (*β*_a_) and cathodic Tafel slope (*β*_b_) by fitting the Tafel curve reflect the surface oxidizing activity of a material and the reduction kinetics of the reactants, respectively (Figure S35, Supporting Information).^[^ ^30^ ^]^ The presence of Cv and lattice Cu significantly increased the *β*_a_, indicating enhanced corrosion resistance of ZVI. This was consistent with the OCP results (Figure S18a, Supporting Information). Notably, the *β*_b_ (62 mV dec^−1^) of CvCu‐ZVI was significantly lower than the theoretical Tafel slope for the Volmer step (120 mV dec^−1^) and significantly larger than that for the Heyrovsky step (40 mV dec^−1^), suggesting more efficient generation of H^*^ rather than H_2_.^[^ ^13^ ^]^ Subsequently, cyclic voltammetry (CV) was employed to investigate the types and quantities of hydrogen species on the ZVI surface. Oxidation peaks corresponding to adsorbed H^*^ and H_2_ were observed in the CV curves of ZVI. Interestingly, the H_2_ oxidation peak disappeared in the CV profile of CvCu‐ZVI, while the intensity of the H^*^ oxidation peak was much higher than that of ZVI (Figure S36, Supporting Information), indicating suppressed H_2_ evolution and enhanced H^*^ generation. Notably, under the same applied potential, the H^*^ oxidation peak of CvCu‐ZVI shifted to −0.03 V, more negative than those of the other ZVI materials, suggesting a stronger reducing capability for H^*^ generation. We also observed that CCu‐ZVI, without vacancies in the carbon shell, produced less H^*^ than ZVI. In contrast, the H^*^ generated by CvCu‐ZVI, in which Cu was doped into the Fe⁰lattice, was 1.94 times that of Cv‐ZVI. The cumulative evidence suggested that lattice Cu impregnation enhances electron release from the Fe core, while Cv sites in the carbon shell extracted these electrons to generate H^*^ in situ.

Due to the significant color change of WO_3_ during its interconversion with the hydrogen bronze phase (H_x_WO_3_) from bright yellow to deep blue and the concurrent decrease in the Raman signals of W–O stretching vibrations, we employed the color change measurement of WO_3_ to investigate the hydrogen spillover behavior of CvCu‐ZVI. The results revealed that the WO_3_ on CvCu‐ZVI underwent a more distinct color change from bright yellow to deep blue compared to other ZVI samples. Concurrently, it exhibited the most intense W–O Raman signals at 715 and 805 cm^−1^ (Figure 3g; Figure S37, Supporting Information), confirming that CvCu‐ZVI generated a greater quantity of H^*^, which rapidly reduced WO_3_ to H_x_WO_3_ via hydrogen spillover. Subsequently, the specific roles of Fe, Cu, and Cv sites in hydrogen spillover were elucidated by analyzing their Gibbs free energy of hydrogen adsorption (Δ*G*_H*_). As expected, Cv exhibited the highest Δ*G*_H*_, which favored H^*^ formation and inhibited H^*^ recombination, indicating that Cv served as the primary site for H^*^ generation in CvCu‐ZVI. Notably, the Δ*G*_H*_ of Fe^0^ was slightly lower than that of Cv sites (Figure 3h), suggesting that H^*^ generated at Cv sites could be further desorbed and reverse spillover to the adjacent Fe^0^ surface.

### 2.5 Origin of Hydrogen Spillover‐Enhanced Dechlorination of PCOs

**Figure 4:**
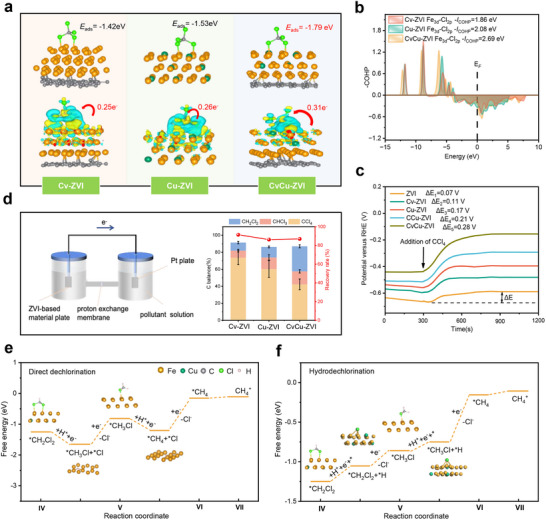
a) The models of CCl_4_ adsorbed on Cv‐ZVI, Cu‐ZVI, and CvCu‐ZVI, along with the corresponding differential charge density map. The difference charge density of charge accumulation and depletion regions is represented in yellow and blue, respectively. b) *I*_COHP_ analysis of the interaction between the Fe 3d orbitals of different materials and the Cl 2p orbitals of CCl_4_. c) Open‐circuit potential curve measurement on the glassy carbon electrode of the loaded material after the addition of CCl_4_. d) Schematic diagram of the double‐chamber galvanic cell and the products of CCl_4_ removal by Cv‐ZVI, Cu‐ZVI, and CvCu‐ZVI. Free energy step diagram of e) the direct dechlorination and f) hydrodechlorination by CvCu‐ZVI during the CCl_4_ removal process.

The entire reaction process was systematically investigated in greater detail through theoretical calculations to elucidate the intrinsic mechanism responsible for the enhanced performance of CvCu‐ZVI. The interaction configuration is pivotal for digging into the origin of the enhanced dechlorination activity. The adsorption energy (*E*_ads_) of CvCu‐ZVI on CCl_4_ was −1.79 eV, which was much lower than that of Cu‐ZVI (−1.53 eV) or Cv‐ZVI (−1.42 eV) (**Figure** **4**a; Figure S38, Supporting Information). This difference indicated that the Cv and lattice Cu synergistically enhanced CvCu‐ZVI binding to CCl_4_. Notably, a more pronounced stretching of the C─Cl bond occurred in the CCl_4_ molecules adsorbed on CvCu‐ZVI than in those adsorbed on Cu‐ZVI and Cv‐ZVI, which suggests that CCl_4_ was more susceptible to dechlorination on CvCu‐ZVI (Table S9, Supporting Information).^[^ ^35^ ^]^ The partial fractional density of states and integrated crystal orbital Hamiltonian population (*I*_COHP_), which are descriptors of reductive activation, confirmed the interaction between Fe and Cl atoms.^[^ ^36^ ^]^ There was a greater energy overlap between the Fe 3d orbitals of CvCu‐ZVI and the Cl 2p orbitals of CCl_4_ (Figure S39, Supporting Information), which demonstrated there was a strong interaction between the CCl_4_ molecule and the Fe^0^ site.^[^ ^37^ ^]^ Furthermore, when CCl_4_ was hybridized to the Fermi energy level of CvCu‐ZVI, the absolute value of the combined *I*_COHP_ of Fe and Cl was much larger (2.69 eV) than that of Cu‐ZVI (2.08 eV) and Cv‐ZVI (1.86 eV) (Figure 4b). This difference suggested that the binding between CCl_4_ and CvCu‐ZVI was more robust compared to other materials.

The differential charge density was used to visualize the electron transfer behavior between Fe^0^ and CCl_4_. Apparently, more charge was transferred from the Fe^0^ site on CvCu‐ZVI (0.31 e^−^) to the Cl atom of CCl_4_ than from Cv‐ZVI (0.25 e^−^) and Cu‐ZVI (0.26 e^−^) (Figure 4b). To further elucidate the electron transfer behavior between CvCu‐ZVI and CCl_4_, electrochemical experiments were conducted.^[^ ^13^, ^38^ ^]^ The change in the OCP on the glassy carbon electrode of the loaded material after the addition of CCl_4_ was recorded (Figure 4c). The potentials of the Cv‐ZVI, Cu‐ZVI, and CvCu‐ZVI electrodes increased immediately to 0.11, 0.17, and 0.21 eV, respectively, after the addition of CCl_4_. In contrast, the potential of the CvCu‐ZVI electrode exhibited a larger increase (0.28 eV), which indicated that Cv and lattice Cu synergistically accelerated electron transfer from ZVI to CCl_4_. The linear scanning voltammetry curve further confirmed this conclusion. The current density response on the CvCu‐ZVI electrode was much higher than that of other materials when CCl_4_ was added to the electrolyte (Figure S40, Supporting Information). These results demonstrated that Cv and lattice Cu synergistically enhanced the DET mechanism, which promoted the dechlorination activity of ZVI on CCl_4_. However, *E*_LUMO_ of CCl_4_ and its dechlorinated intermediates were significantly lower, highlighting their pronounced reductive inertness and the significant challenge of achieving complete dechlorination of CCl_4_ (Figure S20a, Supporting Information). If the complete dechlorination of CCl_4_ was dominated by the enhancement of the DET, the reduction efficiencies of CvCu‐ZVI for CCl_4_, CHCl_3_, CH_2_Cl_2_, and CH_3_Cl would be expected to decrease sequentially. Notably, the reduction rates of CvCu‐ZVI on CCl_4_, CHCl_3_, CH_2_Cl_2_, and CH_3_Cl after 4 h within a single‐chamber galvanic cell were 0.86, 1.12, 1.31, and 1.66 h^−1^ (Figures S41 and S42, Supporting Information), respectively, which exhibited a progressively increasing trend. As expected, the conversion of CCl_4_ to CH_2_Cl_2_ is thermodynamically favourable for either type of ZVI (Figure S48a, Supporting Information).

To distinguish the contribution of DET and H^*^ indirect reduction to the dechlorination conversion of CCl_4_, we constructed experiments in a dual‐chamber galvanic cell with separated ZVI and PCOs. As shown in Figure 4d and Figure S43 (Supporting Information), the products of CCl_4_ removal by Cv‐ZVI, Cu‐ZVI, and CvCu‐ZVI were primarily CHCl_3_ and CH_2_Cl_2_. Among them, CvCu‐ZVI achieved greater CCl_4_ removal, produced less CHCl_3_, and generated more CH_2_Cl_2_ compared with Cv‐ZVI and Cu‐ZVI, suggesting enhanced electron release from CvCu‐ZVI. When the four pollutants were individually used as probes, it was found that reduction efficiencies of CCl_4_ and CHCl_3_ were 57.70 and 45.84%, respectively, while CH_2_Cl_2_ and CH_3_Cl showed negligible reduction. Therefore, we inferred that H^*^ facilitates the reduction of low‐chlorinated intermediates in the complete dechlorination of CCl_4_ by CvCu‐ZVI.^[^ ^39^ ^]^ To further confirm the role of H^*^, we conducted quenching experiments using tert‐butanol (TBA) as the H^*^ scavenger.^[^ ^40^ ^]^ The addition of TBA resulted in a low level of inhibition, which suggested that most of the CCl_4_ reduction occurred via DET rather than H^*^ (Figure S44, Supporting Information). A similar phenomenon was also observed in the TBA quenching experiments for CHCl_3_ reduction. Instead, TBA significantly inhibited the reductive transformations of CH_2_Cl_2_ (≈74%) and CH_3_Cl (≈86%). Furthermore, when the solution pH was adjusted to an acidic value, CvCu‐ZVI exhibited more pronounced increases in the reduction rates of CH_2_Cl_2_ and CH_3_Cl than for CCl_4_ and CHCl_3_ (Figure S45, Supporting Information). This evidence suggested that H^*^ was advantageous for the dechlorination of low‐chlorinated organics, which was further supported by EPR measurements (Figure S46, Supporting Information).^[^ ^41^ ^]^ Considering that the aforementioned H^*^‐mediated dechlorination pathway was closely associated with H^*^ transfer attacking the C─Cl bond to form the C─H bond,^[^ ^42^ ^]^ we investigated the H/D kinetic isotope effect (KIE) during the dechlorination of CCl_4_ and its intermediates. Compared with CCl_4_ (1.34) and CHCl_3_ (1.43), the KIE values of CH_2_Cl_2_ (1.91) and CH_3_Cl (2.16) (Figure S47, Supporting Information) were markedly higher than the threshold value of 1.5, above which hydrogen species are considered to play a decisive role in the rate‐limiting step. This indicates that H^*^ transfer was crucial for the reduction of low‐chlorinated intermediates. Moreover, the KIE value of CvCu‐ZVI (1.75) was lower than that of Cu‐ZVI (3.67) but higher than that of CCu‐ZVI (1.41), suggesting that the introduction of Cv significantly facilitated H^*^ transfer to the surface of Cu‐ZVI through the hydrogen spillover effect. In summary, thanks to the introduction of Cv, the generation, transfer, and utilisation of H^*^ on CvCu‐ZVI were facilitated by the hydrogen spillover effect, resulting in efficient reductive dechlorination.

To understand the energetics associated with the removal of low‐chlorinated intermediates by CvCu‐ZVI, we employed density functional theory calculations to assess the thermodynamics of two possible reaction pathways: hydrodechlorination (hydrogen spillover) and direct dechlorination (DET) (Figure 4e,f). In the hydrodechlorination pathway, H^+^ underwent a Volmer step to generate H^*^ in Cv. This H^*^ then reacted with adsorbed CH_2_Cl_2_, leading to the formation of CH_3_Cl and Cl^−^. Subsequently, H^*^ further reacted with the adsorbed CH_3_Cl, resulting in the formation of CH_4_ and Cl^−^, with the free energy change calculated to be 0.60 eV. This step was the potential‐limiting step of the reaction. As reactions with energy barriers below 0.87 eV are typically considered facile under ambient conditions,^[^ ^43^ ^]^ this hydrodechlorination pathway is energetically plausible, which is consistent with our experimental observation. In contrast, the direct dechlorination pathway involved a proton‐coupled electron transfer step to break the Fe─Cl bond on the CvCu‐ZVI surface, leading to the formation of CH_3_Cl and surface‐adsorbed Cl. Subsequently, the ^*^Cl underwent electrochemical reduction to produce Cl^−^, with a free energy change of 1.05 eV. Therefore, this pathway was less likely to occur spontaneously under ambient conditions.

Here, we presented a reaction mechanism for the complete dechlorination of CCl_4_ by CvCu‐ZVI. The complete dechlorination of CCl_4_ occurred in seven steps (Figure 4e,f; Figure S48, Supporting Information): I) adsorption of CCl_4_ at the Fe^0^ active site, II, III) formation of CHCl_3_ by heterolytic dissociation‐coupled electron‐transfer reduction of CCl_4_ and IV) its further conversion to CH_2_Cl_2_, V) indirect reduction via H^*^ to sequentially form CH_3_Cl and VI) CH_4_, and VII) product desorption. The synergistic coupling between the DET mechanism and hydrogen spillover endowed CvCu‐ZVI with superior reductive activity, achieving complete dechlorination of CCl_4._

### 2.6 Practical Application Potential

Because of the protective carbon shell on its surface, CvCu‐ZVI exhibited high hydrophobicity, which modulated the corrosion reaction between Fe^0^ and water to selectively target pollutants.^[^ ^44^ ^]^ According to the evolution of dechlorination products and H_2_, the electron efficiency of CvCu‐ZVI increased from 58% to 95% but the amount of H_2_ generated was much lower than with other materials (Figure S49, Supporting Information). Moreover, the surface morphology and elemental distribution of the material were largely unchanged throughout the reaction, which indicated that CvCu‐ZVI exhibited excellent structural stability (Figures S50–S53 and Table S10, Supporting Information). We hypothesized that this structural design could be applied to other zero‐valent metals to remove stubborn PCOs. Zero‐valent manganese (ZVM) and zero‐valent nickel (ZVN) were selected as substitutes for ZVI (Figures S54 and S55, Supporting Information). As expected, both CvCu‐ ZVM and CvCu‐ ZVN achieved ≈100% CCl_4_ reduction after 5 h, with dechlorination rates of 0.72 and 0.68 h^−1^, respectively.

**Figure 5:**
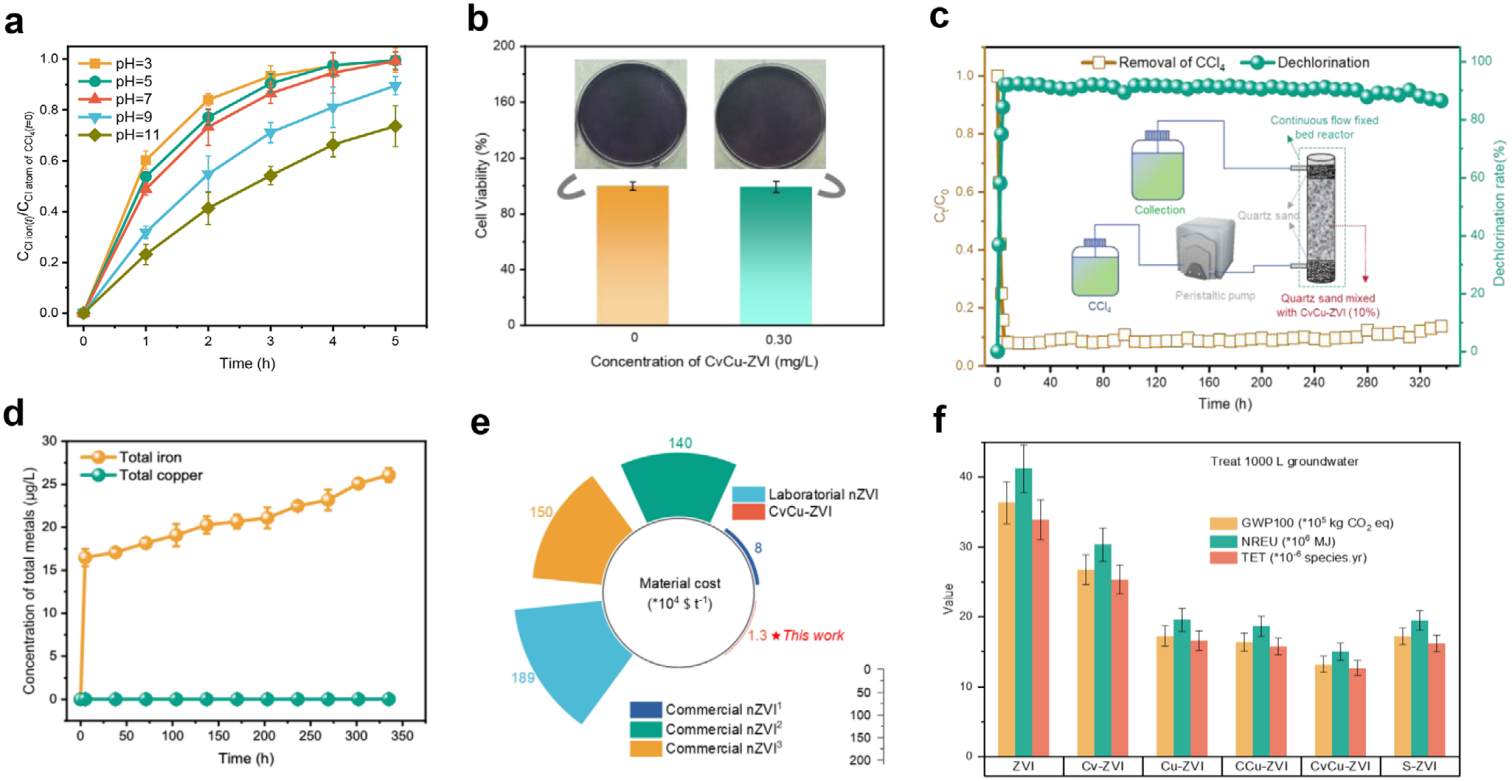
a) Concentration ratios of Cl^−^ ions in solution and Cl atoms in original CCl_4_ removal by CvCu‐ZVI under various pH levels. (Reaction conditions: [CvCu‐ZVI]_0_ = 0.30 g L^−1^, [CCl_4_]_0_ = 5.0 mg L^−1^, T = 298 K.) b) Cytotoxicity experiments were conducted to analyze the impact of CvCu‐ZVI on the growth of *Escherichia coli*. c) Permeable reaction units of CvCu‐ZVI for CCl_4_ removal. Reaction conditions: Flow rate = 2.0 mL min^−1^, HRT = 45 min, [CCl_4_]_0_ = 5.0 mg L^−1^. d) The leaching concentrations of total iron and total copper during continuous flow experiment of CvCu‐ZVI for CCl_4_ removal. e) Techno‐economic analysis of CvCu‐ZVI and other materials. Subscript numbers used in commercial 1–3 represent the material obtained from different companies. f) Life cycle assessment results per functional unit for the production of the treatment of 1000 L of groundwater (containing 5 mg L^−1^ of CCl_4_), including global warming potential (GWP), non‐renewable energy utilization (NREU), and terrestrial ecotoxicity (TET).

To assess the practical application potential of CvCu‐ZVI, we investigated the influence of environmental factors on its dechlorination performance (**Figure** **5**a; Figures S56–S59, Supporting Information). The results showed that CvCu‐ZVI exhibited stable and excellent dechlorination performance over a wide pH range (3–11), in the presence of various inorganic ions, and with varying water quality. The effect of CvCu‐ZVI on the growth of *Escherichia coli* was analyzed to evaluate the ecotoxicity of the material (Figure 5b; Figure S60, Supporting Information).^[^ ^45^ ^]^ As expected, the survival rate of *E. coli* in the experimental group was not significantly different from that of the control group, which was evidenced by the similar shades of dark purple observed on the petri dishes. The outstanding performance of CvCu‐ZVI in dechlorination prompted us to explore the potential integration of its equipment into permeable reaction units. Even after 336 h of continuous operation, the CCl_4_ removal rate (> 90%) and dechlorination rate (> 85%) in groundwater remained high with the continuous flow unit (Figure 5c). Furthermore, the co‐leaching concentrations of Fe and Cu ions detected (Figure 5d) were well below the reclaimed water limits established by the United States Environmental Protection Agency.^[^ ^46^ ^]^

Cost‐effectiveness and environmental impact are critical considerations in the practical application of ZVI technology for pollution control and environmental remediation. To evaluate the economic feasibility of large‐scale synthesis, we conducted a technical and economic analysis. The cost of producing CvCu‐ZVI at scale using our synthesis strategy was <USD13 000 per tonne (Figure 5e), which was 147.90 times cheaper than the cost of commercial ZVI (Tables S11–S13, Supporting Information).^[^ ^9^, ^27^ ^]^ The environmental impacts of various materials in the cradle‐to‐gate life cycle for CCl_4_ reduction were assessed using a life cycle assessment focusing on three environmental aspects: terrestrial ecotoxicity, global warming potential, and non‐renewable energy utilization.^[^ ^47^, ^48^, ^49^ ^]^ Compared with ZVI, the terrestrial ecotoxicity, global warming potential, and non‐renewable energy utilization of the CvCu‐ZVI amplification synthesis process and tonnage groundwater pollution remediation process were 63%, 64%, and 64% lower, respectively (Figure 5f; Figures S61–S63 and Tables S14 and S15, Supporting Information). Regarding the impact of CCl_4_ conversion products on the global greenhouse effect, we calculated that the global warming potential was significantly reduced by 98.8% for every 1 kg of CCl_4_ removed in this study (Figure S64, Supporting Information). Together, these results show that CvCu‐ZVI is suitable for industrial and commercial synthesis, with low production costs and minimal environmental impact.

## 3 Conclusion

We successfully prepared a novel ZVI material modified with Cv and Cu. These modifications effectively addressed the challenge of slow and incomplete dechlorination of PCOs. The tunable physicochemical properties, enhanced electron transfer, and reversible hydrogen spillover resulted in a 38.1‐fold increase in unit‐site activity of CvCu‐ZVI, leading to complete dechlorination of CCl_4_. Excitingly, the intrinsic activity of CvCu‐ZVI reaches 13.7 h^−1^, outperforming state‐of‐the‐art ZVI‐based reductants. Besides CCl_4_, CvCu‐ZVI also completely dechlorinated other PCOs, including perchloroethylene and trichloroethylene, which demonstrated it had excellent dechlorination potential. Sustained removal of CCl_4_ in groundwater over 14 days, integrated into permeable reaction units, showed that CvCu‐ZVI had excellent stability. Importantly, the environmental impact of the synthesis and application of CvCu‐ZVI was much lower than for conventional ZVI. Overall, this study presents an innovative strategy for zero‐valent metal modification that couples enhanced electron transfer and controlled hydrogen spillover to provide a powerful tool for sustainable groundwater remediation.

## 4 Experimental Section

### Materials Synthesis

To prepare CvCu‐ZVI, the grinding process was carried out using a stainless steel tank (100 mL) and zirconia balls (6 mm diameter) in a planetary ball mill (Boyuntong Instrument Technology Company, Nanjing, China) with a milling speed of 500 rpm under an argon atmosphere. Initially, 0.3091 g of citric acid was milled for 4 h, then 4.4891 g micron ZVI was added at room temperature and ground in an argon atmosphere for 2 h, followed by the addition of 0.5109 g Cu at room temperature and ground in an argon atmosphere for 6 h to obtain the CvCu‐ZVI sample. The CvCu‐ZVI was collected in an argon‐filled glove bag and stored in an argon‐filled glove box. The Cu/Fe^0^ ratio ranged from 0.1 to 0.9, and the C/Fe^0^ ratio ranged from 0.06 to 0.30. Cv‐ZVI, Cu‐ZVI, and CCu‐ZVI were synthesized using the same method, with variations including the omission of copper powder, citric acid, or the incorporation of pre‐treatment of micron‐sized ZVI (adding 1 mol L^−1^ hydrochloric acid to micron‐sized ZVI and stirring ultrasonically for 10 min), respectively. In addition, Cv‐ZVM, Cu‐ZVM, CvCu‐ZVM, Cv‐ZVN, Cu‐ZVN, and CvCu‐ZVN were synthesized using the same method.

### Batch Experiments

Batch reactivity studies were conducted in 120‐mL serum vials containing 100 mL of deoxygenated de‐ionized water (pH∼ 7.0, adjusted with a HAc‐NaAc buffer solution) and 20 mL of headspace, which was placed in a thermostatic oscillator at a rate of 200 rpm and maintained at 25.0 ± 0.5 °C for 5 h. The pH of the system remained unchanged unless otherwise stated. A batch experiment was conducted using 0.30 g L^−1^ of material to degrade 5 mg L^−1^ CCl_4_. CCl_4_ degradation kinetics, CCl_4_ degradation products, and CCl_4_ dechlorination rates were analyzed during the first 5 h of the reaction. CvCu‐ZVI was used to degrade CHCl_3_, CH_2_Cl_2_, and CH_3_Cl at 5 mg L^−1^ to investigate the reactivity and selectivity of the material for low chlorine compounds. All batch experiments were repeated three times. Data are presented as mean values with error bars representing standard deviations. The potential of the material as an advanced treatment process for practical water treatment was validated by evaluating Songhua River water in Heilongjiang, China, groundwater sampled from a well in Jiangxi, China, and effluent from a wastewater treatment plant in Harbin, China. The detailed experimental process was identical to the batch experiment described above.

### Materials Characterization

The morphologies and elemental distributions of particles were characterized using scanning electron microscopy (SEM, Sigma500, ZEISS, Germany), aberration‐corrected high‐angle annular dark‐field transmission electron microscopy (JEM‐2100F), high‐resolution transmission electron microscopy, and high‐angle annular dark‐field imaging (Talos, FEI, 200 kV) combined with an energy dispersive X‐ray system (Super‐X EDS, four detector configurations, FEI). The degree of carbon defects was characterized by Raman spectroscopy (LABRAM HR EVO, Horiba Co, Japan), EPR (A300, Bruker, Germany), and thermogravimetric mass spectrometry (NETZSCH STA 449F5‐QMS403). The crystal structure, elemental composition, and specific surface area of the samples were determined using XRD (Rigaku Geigerflex with K_α_ X‐ray source), XPS (ESCALAB 250Xi, Thermo, USA), Auger electron spectroscopy, and specific surface area (Quantachrome 02108‐KR‐1) analysis, respectively. The local coordination environment of iron was identified through the analysis of Fe *K*‐edge EXAFS characteristics, relative shell fitting of Fourier transform spectra, wavelet transform analysis, and Mössbauer spectroscopy. The hydrophobicity of the materials was evaluated by WCA measurements. The electrochemical properties of the materials were characterized using EIS and Tafel analysis. The biotoxicity of the materials was evaluated through bioassay tests. Detailed experimental procedures can be found in the Supporting Information.

### Analytical Methods

The contribution of H^*^ was proved by using quenching experiments, galvanic cell experiments, and kinetic isotope effect experiments. The theoretical calculations, including adsorption energies, differential charge densities, partial fractional density of states analysis, *I*_CHOP_, electrostatic potentials, and Gibbs free energies, were also included in the Supporting Information. The economic feasibility of large‐scale synthesis was evaluated by using technical and economic analysis. A life cycle assessment method was employed to evaluate the environmental impacts of different materials during CCl_4_ removal. Additional details can be found in Supporting Information.

## Conflict of Interest

The authors declare no conflict of interest.

## Supporting information



Supporting Information

## Data Availability

The data that support the findings of this study are available from the corresponding author upon reasonable request.
