# Supplementary material for: Vacancy Engineered Zero‐Valent Iron Steer Hydrogen Spillover toward Per‐ and Polychlorinated Organics Rapid Complete Dechlorination
Source: Adv Sci (Weinh). 2025 Sep 2;12(44):e12668. doi: 10.1002/advs.202512668 (PMC12667448; doi:10.1002/advs.202512668)
Supplement: Supplementary file 1 — Supporting Information [file ADVS-12-e12668-s001.docx]

**Supplementary Materials**

Vacancy Engineered Zero-Valent Iron Steer Hydrogen Spillover toward Per- and Polychlorinated Organics Rapid Complete Dechlorination

*Authors:*

*Zimin Yan^a,1^, Bin Wu^a,1^, Zhiling Li^a,*^, Tianyi Huang^a^, Di Cao^a^, Yunxia Zu^a^, Shih-Hsin Ho^a^, Aijie Wang^a,b,*^*

Affiliations:

^a^ State Key Laboratory of Urban-rural Water Resource and Environment, School of Environment, Harbin Institute of Technology, Harbin 150090, P. R. China

^b^ State Key Laboratory of Urban-rural Water Resource and Environment, School of Civil and Environmental Engineering, Harbin Institute of Technology Shenzhen, Shenzhen 518055, P. R. China

* Corresponding authors:

lizhiling@hit.edu.cn (Zhiling Li)

waj0578@hit.edu.cn (Aijie Wang)

**List of captions**

[**Experimental Section**](#_Toc6597) 3

[**Supplementary Figures** 1](#_Toc18362)2

[**Supplementary Tables** 7](#_Toc32353)6

[**References**](#_Toc32075) 96

# Experimental Section

## Chemicals and Reagents.

The chemical reagents used in this study were of analytical grade or higher unless otherwise noted. Commercially available iron powder (ZVI, D50 ~38 µm, 98% Fe w/w), copper powder (Cu, D50~1 µm, 99% Cu w/w), citric acid (99% w/w, AR), methanol (99%, gas chromatography), n-hexane (99%, gas chromatography), carbon tetrachloride (CCl_4_, 99%), chloroform (CHCl_3_, 99%), dichloromethane (CH_2_Cl_2_, 99%), methyl chloride (CH_3_Cl, 99%), tetrachloroethylene (PCE, 99%), trichloroethylene (TCE, 99%), trichlorobenzene (TOB, 99%), hexachlorobutadiene (HBCD, 99%), trichlorophenol (TCP, 99%), 5,5-dimethyl-1-pyrroline-N-oxide (DMPO, 97%), sodium hydroxide (NaOH, 99%), hydrochloric acid (HCl, 99.5%), sodium sulfate, sodium chloride, sodium bicarbonate (NaHCO_3_, 99%), tert-butanol (TBA, 99%), humic acid (HA, 99%), LB broth(99%), potassium dihydrogen phosphate(KH_2_PO_4_, 99%), disodium hydrogen phosphate(Na_2_HPO_4_, 99%), acetic acid(HAc, 99%), sodium acetate(NaAc, 99%), 3-(4,5-Dimethylthiazol-2-yl)-2,5- diphenyltetrazolium bromide (MTT, 99%)，deuterium oxide (D_2_O)，Periodate(99%) and potassium chloride(KCl, 99%) were supplied by Aladdin (Shanghai, China). Ultra-high purity nitrogen was obtained from Harbin Special Gas Company (Harbin, China). All solutions were prepared with ultra-pure water and deoxygenated by bubbling nitrogen at room temperature for 0.5 h.

## Characterizations of materials.

Aberration-corrected TEM (AC-TEM) images were obtained using a double aberration-corrected Titan Themis Z electron microscope, operating at 60 kV, with monochromator used at an excitation of 0.4. The crystal structure of the materials was determined using an X-ray diffractometer (XRD, Rigaku Geigerflex with K_α_ X-ray source) in the test range of 10-90°. The Raman spectra (LABRAM HR EVO, Horiba Co, Japan) of the materials were measured using a Raman spectrometer in the frequency range of 1000 cm^-1^ to 2400 cm^-1^. The specific surface area (BET, Quantachrome 02108-KR-1) and pore volume of the materials were measured by N_2_ adsorption-desorption using a Micromeritics model 2460 at 88 K. The surface element composition of the sample was determined using X-ray photoelectron spectroscopy (XPS) and Auger electron spectroscopy (AES). XPS was performed and analyzed using an Al Kα X-ray source. All binding energies were calibrated using the C1s peak (284.8 eV), and the spectra were fitted using XPS-PEAK4.1 software. The element composition of the sample was determined using inductively coupled plasma optical emission spectrometry (ICP-OES, Varian 720-ES). The thermogravimetric mass spectrometry spectrum was obtained using NETZSCH STA 449F5-QMS403 to trace CO and CO_2_ signals during the pyrolysis of materials. The hydrophobicity of the materials was evaluated by WCA measurements. The electron paramagnetic resonance (EPR, A300, Bruker, Germany) spectra of carbon vacancies were monitored using an EPR spectrometer (A300, Bruker, Germany). 5,5-Dimethyl-1-pyrrolidine-N-oxide (DMPO) was used as a spin trap to detect ∙H radicals.

For Mössbauer spectroscopic analysis, after drying in the glove box, 30±5 mg of CvCu-ZVI powder was put into the sample holder, flattened and fixed with adhesive tape. The as-prepared sample was stored in a vacuum bottle and immediately analyzed using a conventional spectrometer (SEE Co W304) in transmission geometry with constant acceleration mode at 77 K. A 57Co(Rh) source with an activity of 25 mCi was used.

## Analytical Methods.

30 mg WO_3_ was dispersed in 3 mL ethanol, and after ultrasound for 30 min, 1.5 mL WO_3_ ink was applied to the material. Finally, a color change experiment was performed and the chemical change of sample surface was measured by Raman spectrometer in the frequency range of 500-1000 cm^-1^.

In situ Fourier Transform Infrared spectroscopy (FTIR, Thermo, Nicolet iS 5 FT-IR) was employed to analyze the reaction in the range of 600–4000 cm^-1^. The procedure involved placing a thin layer of CvCu-ZVI, CCu-ZVI, Cv-ZVI, Cu-ZVI, or ZVI onto an appropriate FTIR sample holder. A 5 mg L^-1^ CCl_4_ solution was then introduced to the sample as required. All measurements were conducted promptly and repeated three times to minimize the influence of atmospheric water.

To evaluate the biotoxicity of CvCu-ZVI, its effects on *Escherichia coli* were assessed using a biotoxicity assay. The bacterial strain was inoculated into LB liquid medium and incubated at 37°C with shaking at 150 rpm for 10 h. Subsequently, 5 mL of the bacterial culture was transferred to 50 mL of sterile liquid medium in conical flasks. To each flask, CvCu-ZVI was added at concentrations of 0.10-, 0.20-, 0.30-, 0.60-, and 1.20 mg L^-1^, respectively, with a control group containing no CvCu-ZVI. The experiments were conducted over a duration of 5 h, and cell viability was determined using the 3-(4,5-dimethylthiazol-2-yl)-2,5-diphenyl- tetrazolium bromide assay at a concentration of 0.5 mg mL^-1^.

For experiment in a galvanic cell, the reduction of pollutants by ZVI-based materials was studied using an H-type electrolytic cell. Two electrolyte compartments (each 100 mL) were separated by proton exchange membranes (PEM, Nafion 117, DuPont Inc., USA). The carbon felt plate (1 cm^2^) filled with ZVI-based material was used as the anode, and the Pt plate was used as the cathode. With the help of ultrasonic treatment, 30.0 mg of ZVI-based material was dispersed in a mixture of 30 μL of 5 wt % Nafion solution and 1.5 mL of isopropanol to prepare the electrode suspension. Then, the suspension was cast onto carbon felt and dried in a nitrogen atmosphere. The anode and cathode were connected with titanium wire to complete the external circuit. The electrolyte was composed of 50 mM HEPES buffer solution (pH 7.0), containing 5 mM Na_2_SO_4_. Only the pollutant (5mg L^-1^) was added to the cathode chamber. The cathode chamber was magnetically stirred at a rotational speed of 300 rpm, and samples were collected for the analysis of pollutants and their products. All these procedures were carried out in an anaerobic chamber at 25 °C.

## Element content determination.

**Fe^0^ content determination.** About 10 mg materials were added into a 200 mL serum vial. After holding the particles onto the side of the vial, 10 mL 37% HCl solution was added while avoiding contact with any particles, until the vial sealed with a Teflon Mininert valve. Then the sample was digested overnight, and the ZVI content of each sample was calculated by measuring the H_2_ in the headspace using a gas chromatography-thermal conductivity detector (GC-TCD) system (FuLi GC9790plus) with a carrier gas (argon). The peak position of the H_2_ fraction was ~0.5 min, and the injection volume of the sample was 500 μL. The injection port temperature, pre-column pressure, and column flow rate were 120 °C, 215 kPa, and 20 mL min^-1^, respectively. The column chamber was kept at a constant temperature of 80 °C. The reference flow rate of the detector was 27.6 mL min^-1^ and the temperature was 130 °C.

**The total Fe and Cu contents.** The total iron and copper content was determined by digesting 10 mg of the sample in 10 mL of aqueous solution.^[1]^ The total Fe and Cu concentrations in each sample were then measured using inductively coupled plasma optical emission spectrometry (ICP-OES).

**Carbon defect density calculations.** The distance between defects (L_D_ cm^−2^) and carbon atoms was calculated using as:

$$\begin{aligned} \frac{I_{D}}{I_{G}}=C_{A}\frac{\left( r_{A}^{2}-r_{S}^{2} \right)}{\left( r_{A}^{2}-2r_{S}^{2} \right)}\left[ e^{-\frac{\pi r_{S}^{2}}{L_{D}^{2}}}-e^{-\frac{\pi\left( r_{A}^{2}-r_{S}^{2} \right)}{L_{D}^{2}}} \right]\text{ } \end{aligned}$$

The polygons have similar sizes, approximately 0.25 nm. The parameter *r_S_* denotes the radius of the “structurally disordered area”, while *r_A_* refers to the surrounding “activated area”.

The ion-induced defects measure approximately 0.25 and 0.775 nm, respectively. The *I*_D_/*I*_G_ values were determined by Raman spectroscopy.^[2, 3]^

The defect density $\eta_{V}$ (cm^−2^) is given by:

$\begin{aligned} \eta_{V}={10}^{14}/\pi L_{D}^{2} \end{aligned}$

## Determination of pollutants concentration and identification of degradation products

The analysis of CCl_4_, CHCl_3_, CH_2_Cl_2_, CH_3_Cl, PCE, TCE, TOB, HBCD, and their reaction intermediates and liquid products was carried out by headspace gas chromatography (GC, 7890A). Detection was performed using an Agilent Technologies GC system. The parameters of the headspace sampler (6980N) were as follows: headspace equilibrium temperature: 65℃, headspace equilibrium time: 21 min, and transfer line temperature: 105℃. The concentration of CO and hydrogen produced in the headspace materials was determined using a FULI 9790 gas chromatograph equipped with a TCD detector and a packed column (3 mm × 1 m, Hangzhou Jiaxuan Technology Co., LTD.). CO and H_2_ were injected into the column through the second channel. Nitrogen was used as the carrier gas. The oven temperature was maintained at 70℃, and the TCD temperature was set to 140℃. Calibration was performed using prepared CO and H_2_ standards. The concentration of TCP and its products was determined using high-performance liquid chromatography (Waters 1525, USA) with a C18 column (4.6 mm × 150 mm, 5 µm particle size) and an ultraviolet detector (Waters 2487, USA). The mobile phase consisted of methanol and formic acid (0.1% (v/v), 70:30). The flow rate was 0.2 mL min^-1^, and the detection wavelength was 289 nm. Formic acid (HCOOH) and chloride ion (Cl^-^) were analyzed using ion chromatography (IC) (ICS-1100) equipped with a Dionex IonPacTM AS11-HC column and a potassium hydroxide ion-eluent generator.^[4]^ The supernatant underneath the serum bottles was drawn using a 1 mL gastight syringe, and the reactors were sacrificed sequentially. materials were then filtered into 2 mL centrifuge tubes using a 0.22 μm nylon filter membrane after adjusting the pH with 0.15 M KOH to precipitate iron ions.

**Key parameter definitions.** The *k*_1_ is expressed as pseudo-first order rate constants of pollutant reduction.

$$\begin{aligned} \ln\frac{C_{t}}{C_{0}}=k_{1,pollutant}\times t \end{aligned}$$

Dechlorination rate constant (*k*_Cl_). The dechlorination rate constant (*k*_Cl_) was normalized with a pseudo-first order kinetic for the comparation of dechlorination efficiency on different materials according to the following as:

$$\begin{aligned} -ln(1-\alpha(t))=k_{\mathrm{Cl}}t\text{ } \end{aligned}$$

In which, α(t) refers to the dechlorination ratio. *t* refers to reaction time. *k_Cl_* refers to the dechlorination rate constant.

Calculation of electron efficiency (*ε_e_*)

$$\begin{aligned} \varepsilon_{e}=\frac{\sum_{i} \eta_{i}p_{i}}{\sum_{i} \eta_{i}p_{i}+2M_{H_{2}}}\text{ } \end{aligned}$$

Where $\eta i$ is the stoichiometry for product *i*, *p*i is the corresponding molar quantity of the product.

## Electrochemical experiments.

All electrochemical experiments were performed in a conventional three-electrode configuration using an electrochemical workstation (CHI600E, Shanghai, China). Preparation of working electrodes: 25 mg of sample was added to 300.0 µL of Nafion^®^ solution (5.0 wt%) and subsequently mixed with 2.0 mL of anhydrous ethanol and sonicated for 2 h. Then a 25 μL suspension was dropped onto the glassy carbon electrode (polished before) for 2-3 times and air-dried in the anaerobic glovebox. Pt sheet and Ag/AgCl electrode were used as counter electrode and reference electrode, respectively. Prior to electrochemical characterization, the test solution (50 mM NaCl) was deoxidation by filling with N_2_. After that, the open circuit potential (OCP) measurements, electrochemical impedance spectroscopy (EIS), and Tafel analysis were testing in sequence. Linear sweep voltammetry (LSV) was measured at a potential of -2.0-1.0 V with a scan rate of 50 mV s^-1^ after the addition of CCl_4_. During this process, the test solution was in the state of N_2_ filling and deoxidation.

## EXAFS measurement and data analysis.

The X-ray absorption spectra (XAS) including X-ray absorption near-edge structure (XANES) and extended X-ray absorption fine stucture (EXAFS) of the materials at Fe *K*-edge (7112eV were colleted at the Singapore Synchrotron Light Source (SSLS)center,where a pair of chanel-cut Si (111) crystals was used in the monochromator. The Fe *K*-edge XANES data were recorded in a transmission mode. Fe foil, FeO and Fe_2_O_3_ were used as references.

The XAFS data normalization and Fourier-transformed data fitting were performed by using Demeter (version 0.9.26) software package. During fitting, k^3^ weights, k-range (3–~11 Å^−1^), and R range (1–~6 Å) were applied for all of the data. The χ(k) data format was imported into the Larch Python code for Wavelet Transform analysis. The parameters were listed as follows: R range was 1-6 Å; k range was 2–14 Å^−1^; k-weight was 3; and the Morlet function with κ =6, σ = 1 was utilized as the mother wavelet to provide the overall distribution.

## DFT calculation

First-principles density functional theory (DFT) calculations were performed using the Vienna Ab initio Simulation Package (VASP) with the projector augmented wave (PAW) method.^[5,6]^ The exchange-functional was treated within the generalized gradient approximation (GGA) employing the Perdew-Burke-Ernzerhof (PBE) functional.^[7]^ The long-range van der Waals interactions are accounted for through the DFT-D3 approach.^[8]^ A plane wave basis set with an energy cutoff of 500 eV was employed, and the geometry relaxation was performed until the forces on each atom were below 0.03 eV/Å. The Brillouin zone was sampled using 3×3×1 k-point grid. Self-consistent calculations were conducted with an energy convergence threshold of 10^-5^ eV. To prevent interactions between periodic structures, a vacuum region of 15 Å was added along the z direction. The computation of COHP is performed via LOBSTER software.^[9]^

The adsorption energy of the Li atom is obtained by the following formula:

$E_{\mathrm{ads}}=E_{*\mathrm{CCl}_{4}}-E_{*}-E_{\mathrm{CCl}_{4}}$

Where, $E_{*\mathrm{CCl}_{4}}$represents the total energy of CCl_4_ adsorbed on the slab, $E_{*}$ represents the total energy of the slab, and $E_{\mathrm{CCl}_{4}}$represents the energy of CCl_4_.

To effectively isolate periodic structures and preclude their interaction, a vacuum buffer of 15 Å was inserted along the z-axis.

 The free energy of the intermediates is calculated：

ΔG = ΔEDFT + ΔZPE − TΔS

Where ΔEDFT, ΔZPE and ΔS are the changes of the reaction energy obtained from DFT calculations, zero-point energy, and the changes of entropy from the initial state to the final state, respectively. T is temperature and the T of 298.15 K was used in all computations.

## Techno-economic analysis of scalable synthesis of CvCu-ZVI

Through technical and economic analysis, the economic potential of large-scale synthesis of CvCu-ZVI was evaluated. Under the conditions of the scalable experiment, we estimated the cost of producing 1 tonne of CvCu-ZVI per day. Costs were categorized into input chemicals (Table S11), equipment, installation, solid-liquid separation, electricity, and other operating costs (labour and maintenance). As prices varied by region, delivery costs were not included in the analysis. Additionally, the separation cost was not separately accounted for, as it was included in the cost of equipment and electricity. Prices for commercial nFe^0^ materials were sourced from previous reports.^[10]^ Details are provided below.

**Cost of Input Chemicals.** Chemical prices were adjusted based on industrial production studies and literature reports. The chemical ratio was based on scalable synthesis conditions. Argon flow was set at 120 mL min^-1^ for a flow time of 3 min and at 500 mL min^-1^ for a flow time of 10 min. The cost of input chemicals was calculated as follows:

$$Input chemical cost [\$ per tonne of material]=\sum all chemcials price$$

$$=42.34 +5915.68+4447.81+596.74 +13.52$$

$$=\boldsymbol{11016.09} [\$ \boldsymbol{per} \boldsymbol{tonne} \boldsymbol{of} \boldsymbol{material}]$$

**Cost of Equipment.** The equipment purchase cost **was calculated** based on our assumed production process. Specific technical data and equipment prices **were presented** in Table S12. The cost of purchasing the equipment **was converted** to the cost of producing one ton of CvCu-ZVI. We **assumed** that these devices **had** a lifespan of 20 years, **with no residual value** at the end of the factory's operational life, a factory capacity factor of 0.9, and a discount rate (i) of 7%.^[11]^ The capital recovery factor (CRF) **was calculated**as:

$$CRF_{eauipment}=\frac{i(1+i)^{lifetime}}{i(1+i)^{lifetime}-1}=\frac{0.07(1+0.07)^{20}}{(1+0.07)^{20}-1}=0.094$$

The equipment cost of per tonne of CvCu-ZVI can be calculated as: $\text{Equipment cost [\$ per }\text{tonne}\text{ of material]}$

$$=\frac{CRFequipment\times total equipment price []}{capacity factor\times365day\times production capacity[\frac{t}{day}]\text{ }}$$

$$=\frac{0.094\times(36982.13+3868.54+7571.28)}{0.9\times365\times1}=\boldsymbol{12.83} [\$ \boldsymbol{per} \boldsymbol{tonne} \boldsymbol{of} \boldsymbol{material}]$$

**Installation costs.** We assumed that the Lang coefficient of the installation cost of the equipment was 1 and that the total capital cost was equal to the equipment cost.

$$Installation cost [\$ per tonne of material] = Lang factor x total captial cost$$

$$=1\times12.83=\boldsymbol{12.83} [\$ \boldsymbol{per} \boldsymbol{tonne} \boldsymbol{of} \boldsymbol{material}]$$

**Electricity costs.** The electricity price was determined by the average price of industrial electricity in Heilongjiang Province in 2025 (0.63 kWh^-1^). The electricity charge was calculated using the following formula: Electricity cost [$ per tonne of material]

$$=\sum all equipment power consumed [kW h] \times electricity price$$

$$=\left( 13.3\times24+0.75\times12\times63+2\times1 \right)\times0.63\times0.1369=\boldsymbol{76.60} [\$ \boldsymbol{per} \boldsymbol{tonne} \boldsymbol{of} \boldsymbol{material}]$$

**Other Operating Costs.** Other operating costs (such as labor, maintenance, and waste disposal) were assumed to be 15% of the total capital cost calculated above.

$$Other operational cost [\$ per tonne of material]$$

$$=15\%\times(11016.09+12.83+12.83+76.60)$$

$$=1667.75 [\$ \boldsymbol{per} \boldsymbol{tonne} \boldsymbol{of} \boldsymbol{material}]$$

Plant-gate levelized cost. The cost of gate levelization for the production of 1 tonne of CvCu-ZVI was calculated as the sum of all the above costs.

$$Plant-gatelevelized cost [pertonneofmaterial]$$

$$=(11016.09+12.83+12.83+76.60+1667.75)$$

$$=12786.10 [\$ \boldsymbol{per} \boldsymbol{tonne} \boldsymbol{of} \boldsymbol{material}]$$

## Life-cycle assessment

The objectives and scope of the life cycle assessment research conducted in this work are presented in Tables S14-15. Additionally, the system boundary of the life cycle assessment is defined as "from cradle to gate", encompassing material preparation and CCl_4_ degradation. The impact assessment analysis was conducted using the software Openlife cycle assessment 2.1.1. In this assessment method, GWP100 was calculated using the CML-IA baseline. Non-renewable energy use (NREU) (or abiotic depletion of fossil fuels) and terrestrial ecotoxicity were calculated using the ReCiPe 2016 Endpoint (E) method. Both evaluation methods are derived from the LCIA integration package of Openlife cycle assessment LCIA Methods v2.4.0. For uncertain data in the carbon footprint process, an average distribution was used to set the uncertain flow, and the mean value obtained after 500 Monte Carlo simulations was considered the final data. In this study, comparative analysis with our testing materials (CvCu-ZVI, CCu-ZVI, Cu-ZVI, Cv-ZVI, and ZVI) and similar preparation processes materials from other researchers, including S-nZVI.^[12]^ The evaluation results for functional units of 1000 L of groundwater treatment were shown in Tables S14-15, respectively.

# Supplementary Figures

a


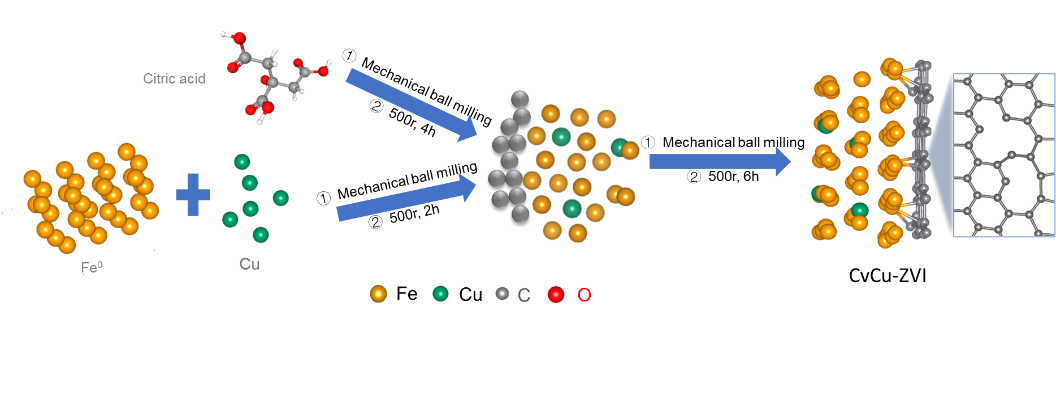


**d**

**e**

**c**


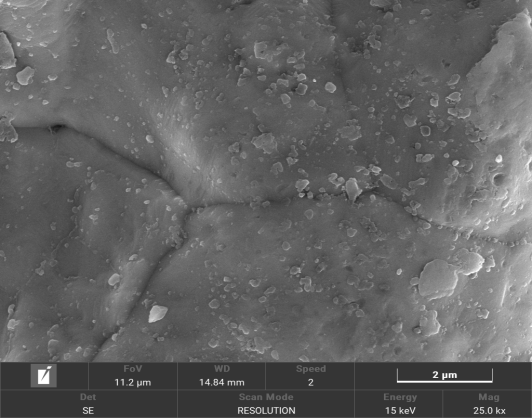

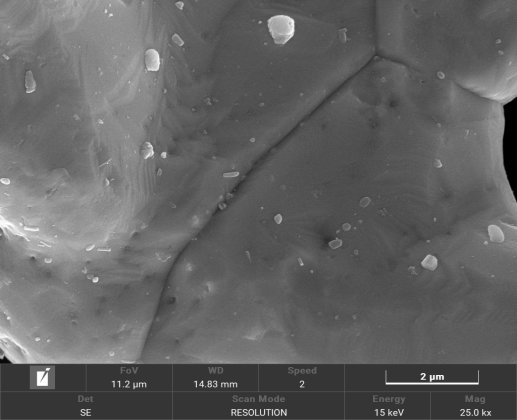

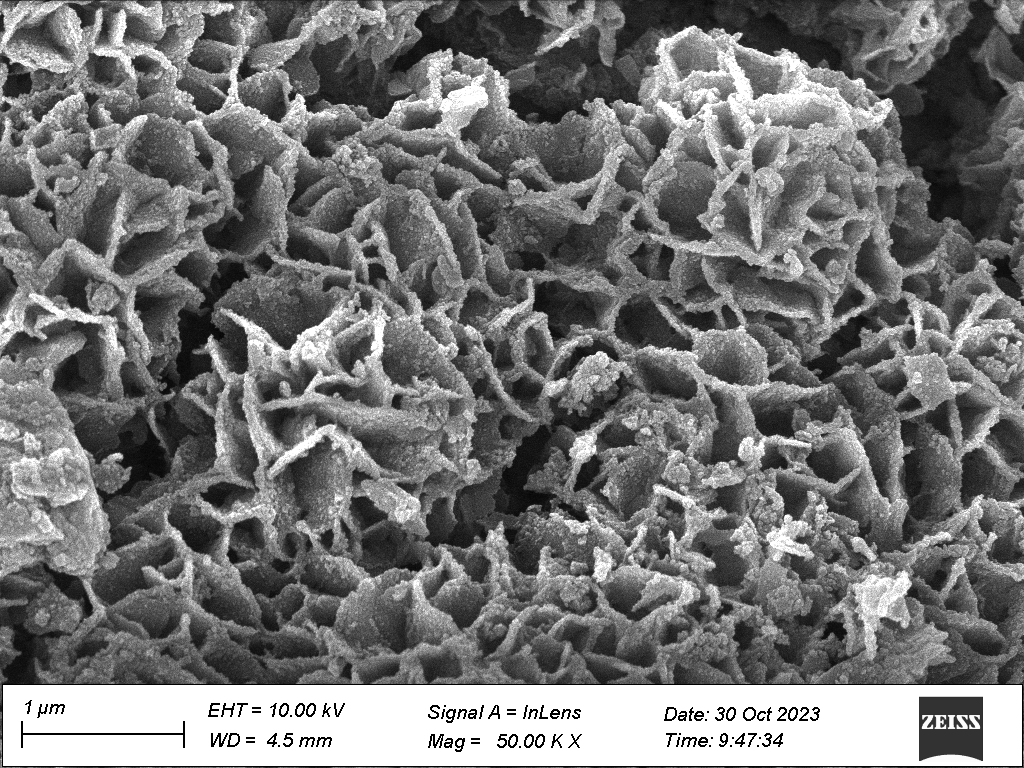


**b**


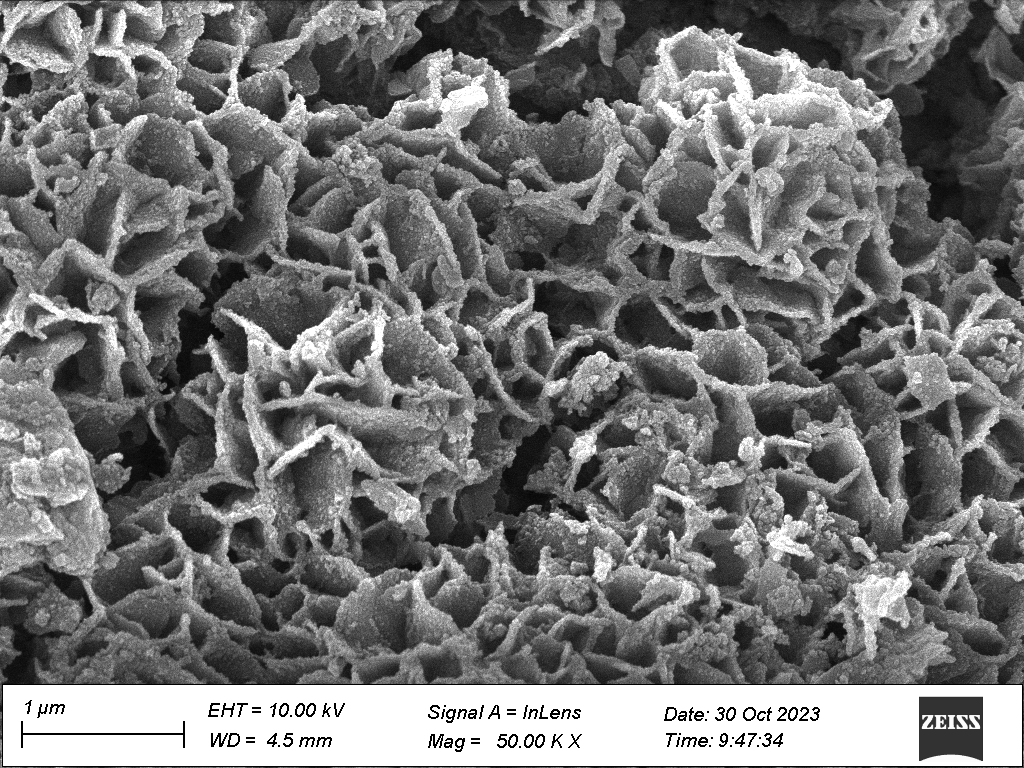

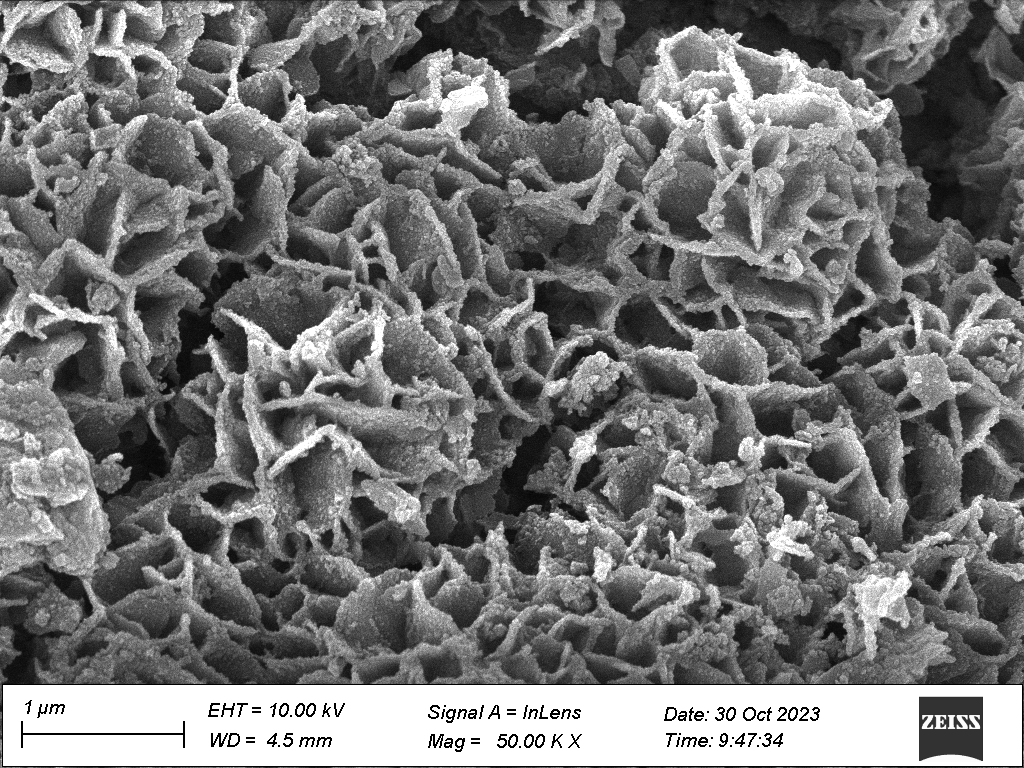

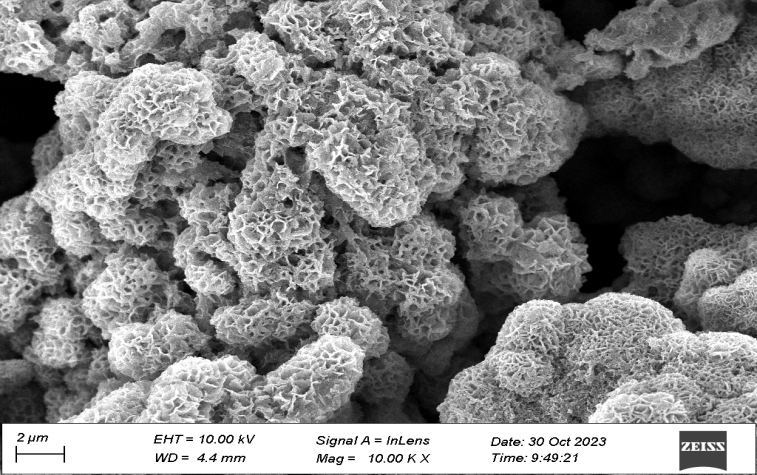

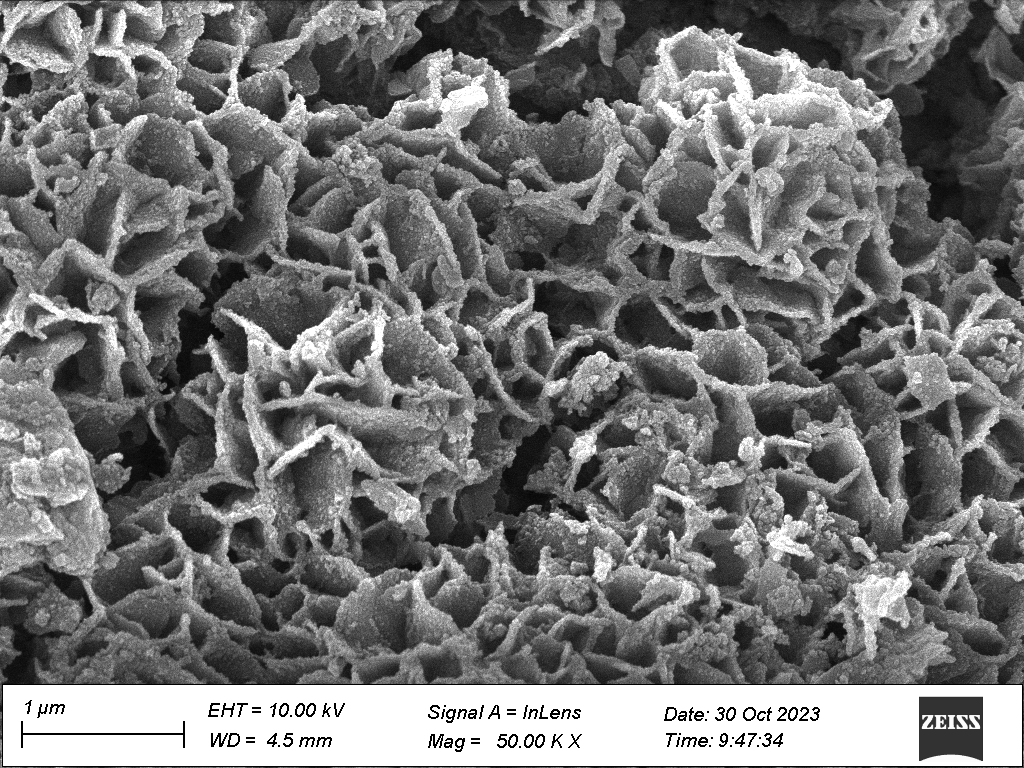

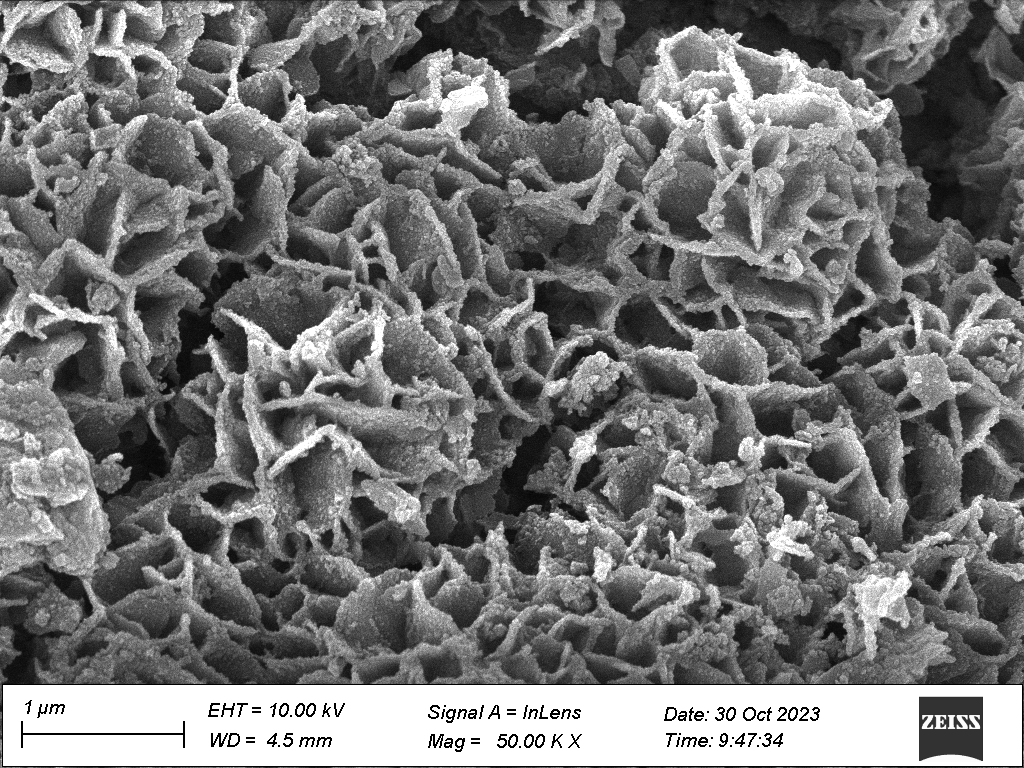


**Figure S1.** (a) The formation process of lattice CvCu-ZVI. SEM images of (b) ZVI, (c) Cu-ZVI, (d) Cv-ZVI and (e) CvCu-ZVI.

**
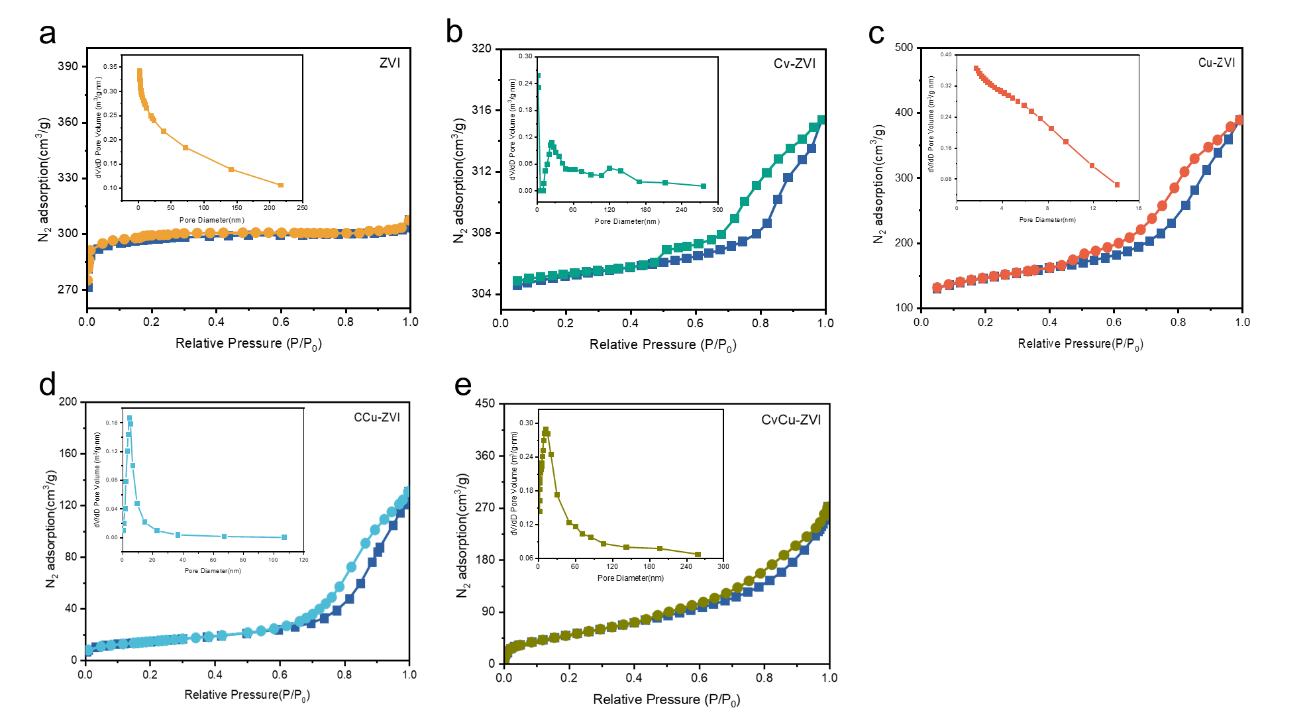
**

**Figure S2.** N_2_ adsorption-desorption curves and pore size distribution curves of (a) ZVI, (b) Cu-ZVI, (c) Cv-ZVI, (d) CCu-ZVI and (e) CvCu-ZVI.

**Figure S3.** XRD pattern results of different materials.


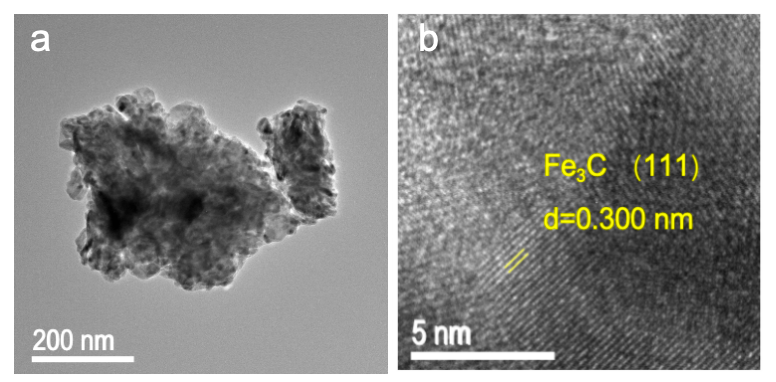


**Figure S4.** High-resolution transmission electron microscopy images of CvCu-ZVI.


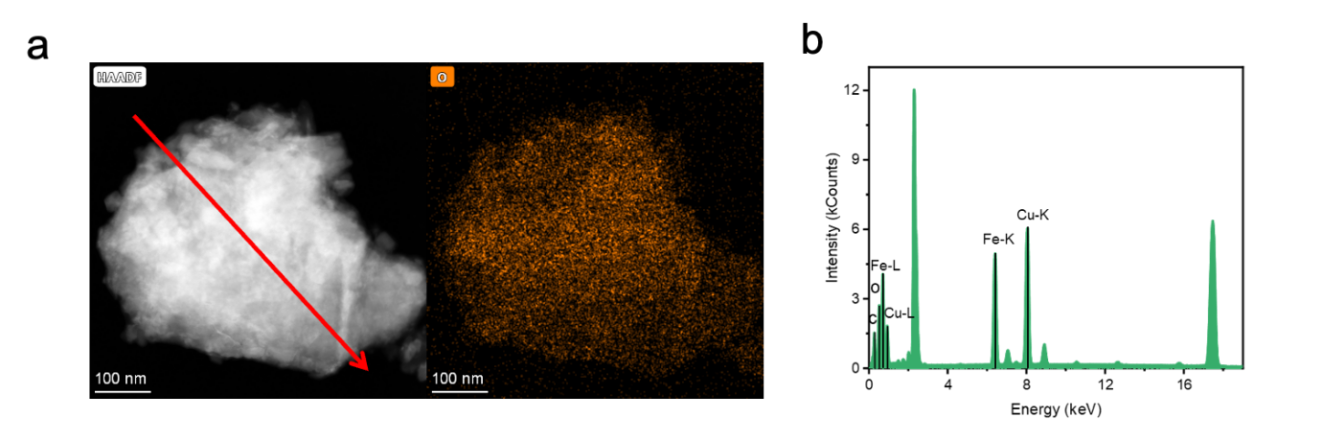


**Figure S5.** (a) High-angle annular dark-field scanning transmission electron microscopy images and the corresponding elemental mapping, and (b) EDS spectrum result of CvCu-ZVI.


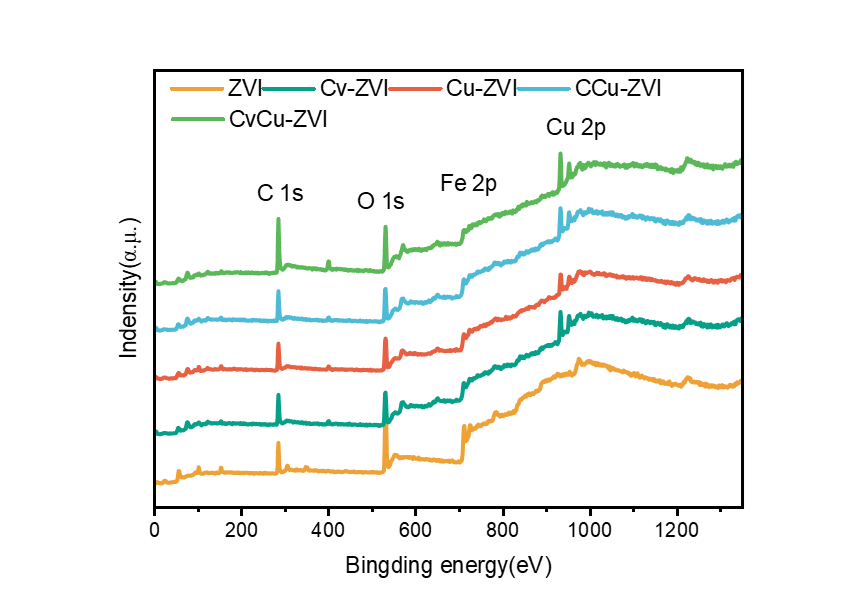


**Figure S6.** Full-scan XPS spectra of different materials.

**
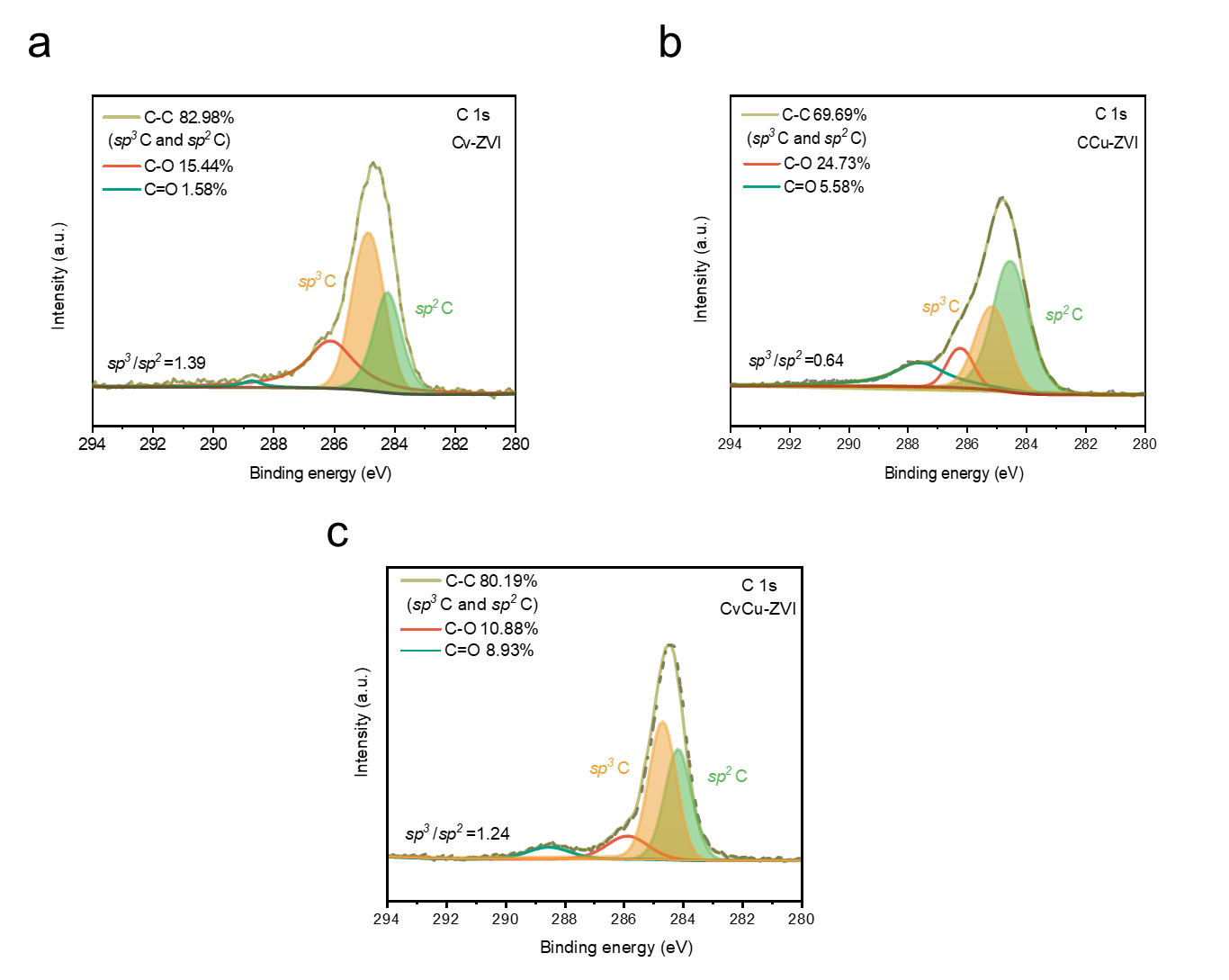
**

**Figure S7.** High-resolution XPS C 1s spectra of (a) Cv-ZVI, (b) CCu-ZVI, and (c) CvCu-ZVI.


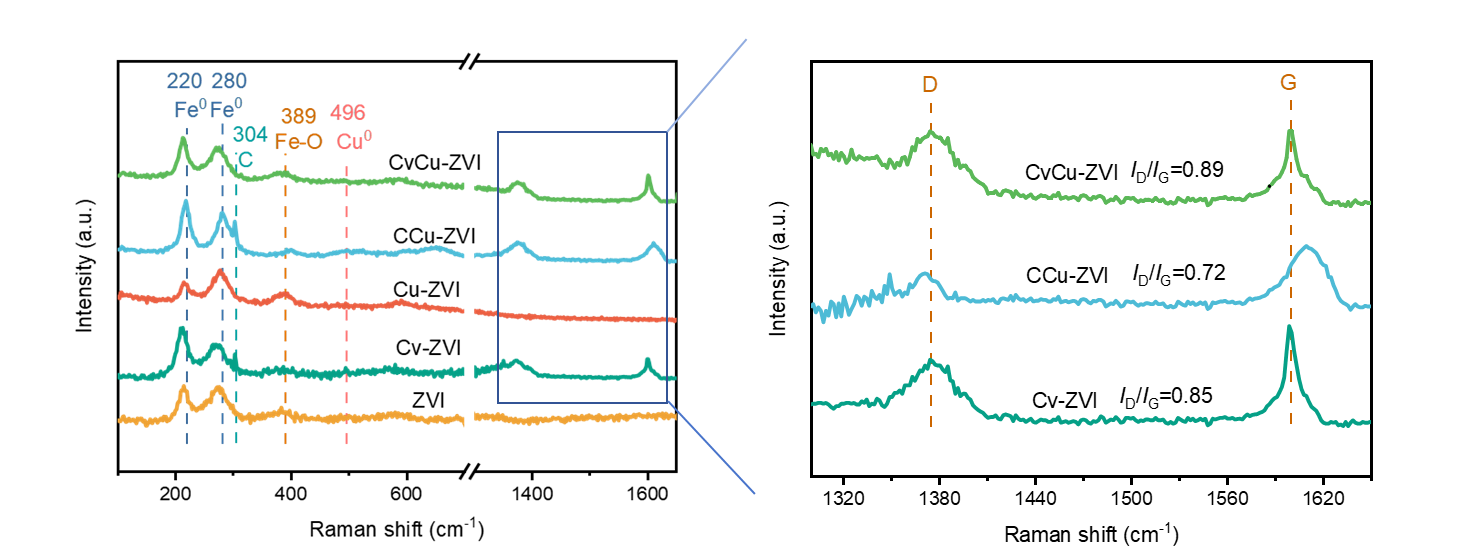


**Figure S8.** Raman spectra of different materials and the *I*_D_/*I*_G_ ratios for Cv-ZVI, CCu-ZVI, and CvCu-ZVI.

Note: Compared to ZVI, the Raman spectrum of Cv-ZVI exhibited a carbon peak at 304 cm^-1^ accompanied by a decrease in the intensity of the corresponding peak for the Fe-O bond at 389 cm^-1^. Interestingly, CCu-ZVI displayed almost no Fe-O peaks and exhibited a distinct carbon peak; on the contrary, the carbon peaks of CvCu-ZVI prepared using untreated iron powder almost completely disappeared, further corroborating the XPS results.

**Figure S9.** EPR spectra of ZVI, Cv-ZVI, Cu-ZVI, CCu -ZVI and CvCu-ZVI.

**
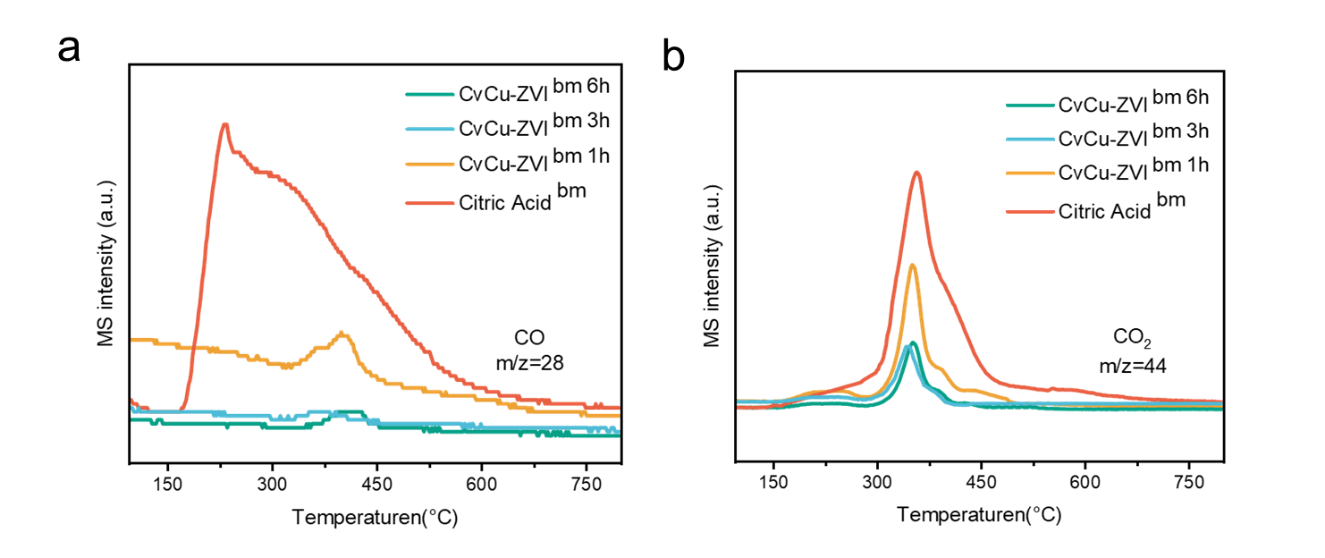
**

**Figure S10.** Thermogravimetric mass spectrometry analysis of gases evolved from CvCu-ZVI prepared by different milling times and individual citric acid.

Note: The signal intensity of CO and CO_2_ released by CvCu-ZVI remained consistent between 3 h and 6 h, indicating that Cv formation primarily occurred in the first half of ball-milling period.

**
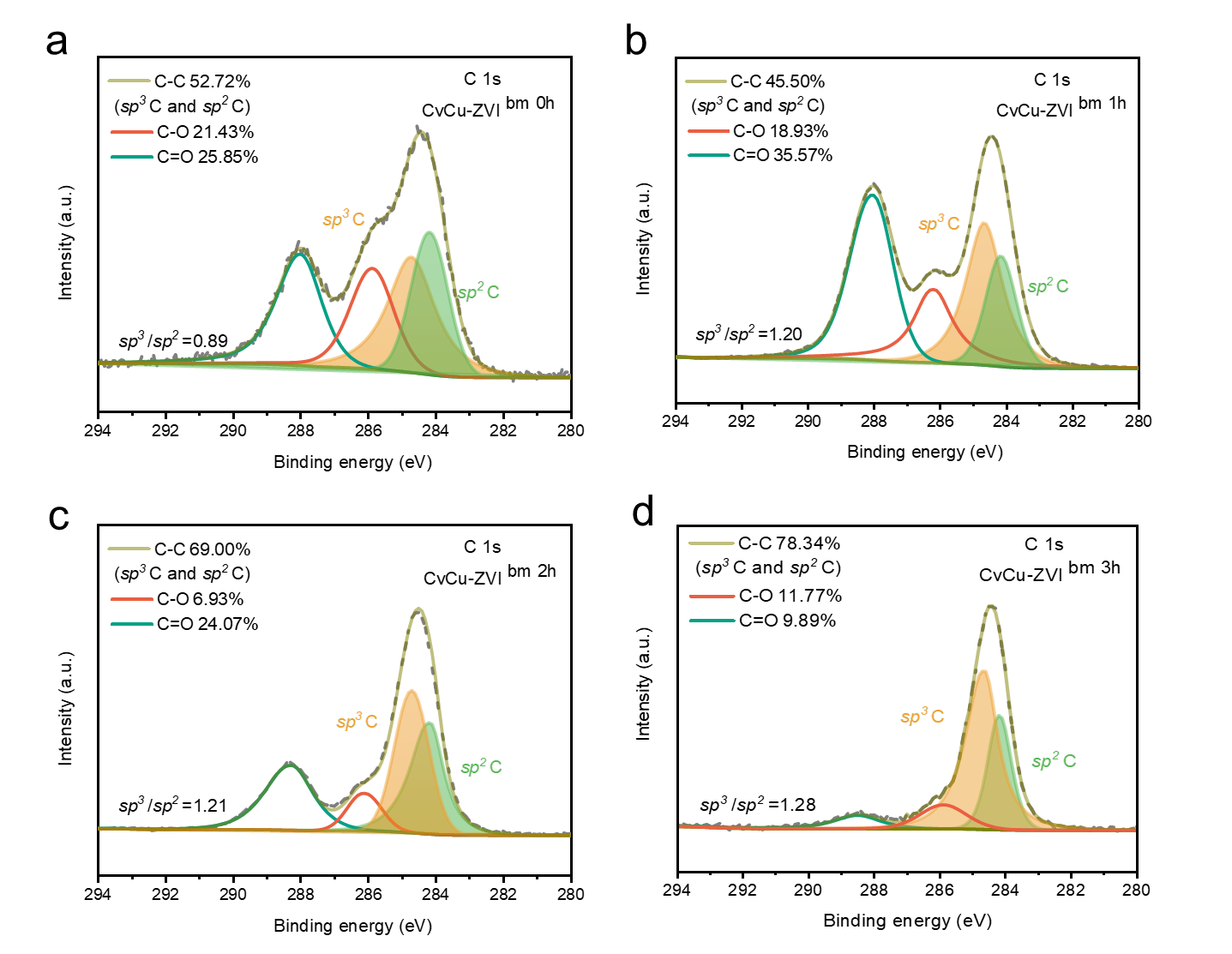
**

**Figure S11.** High-resolution XPS C 1s spectra of CvCu-ZVI prepared by (a) 0 h, (b) 1 h, (c) 2 h, and (d) 3 h of milling time.

**
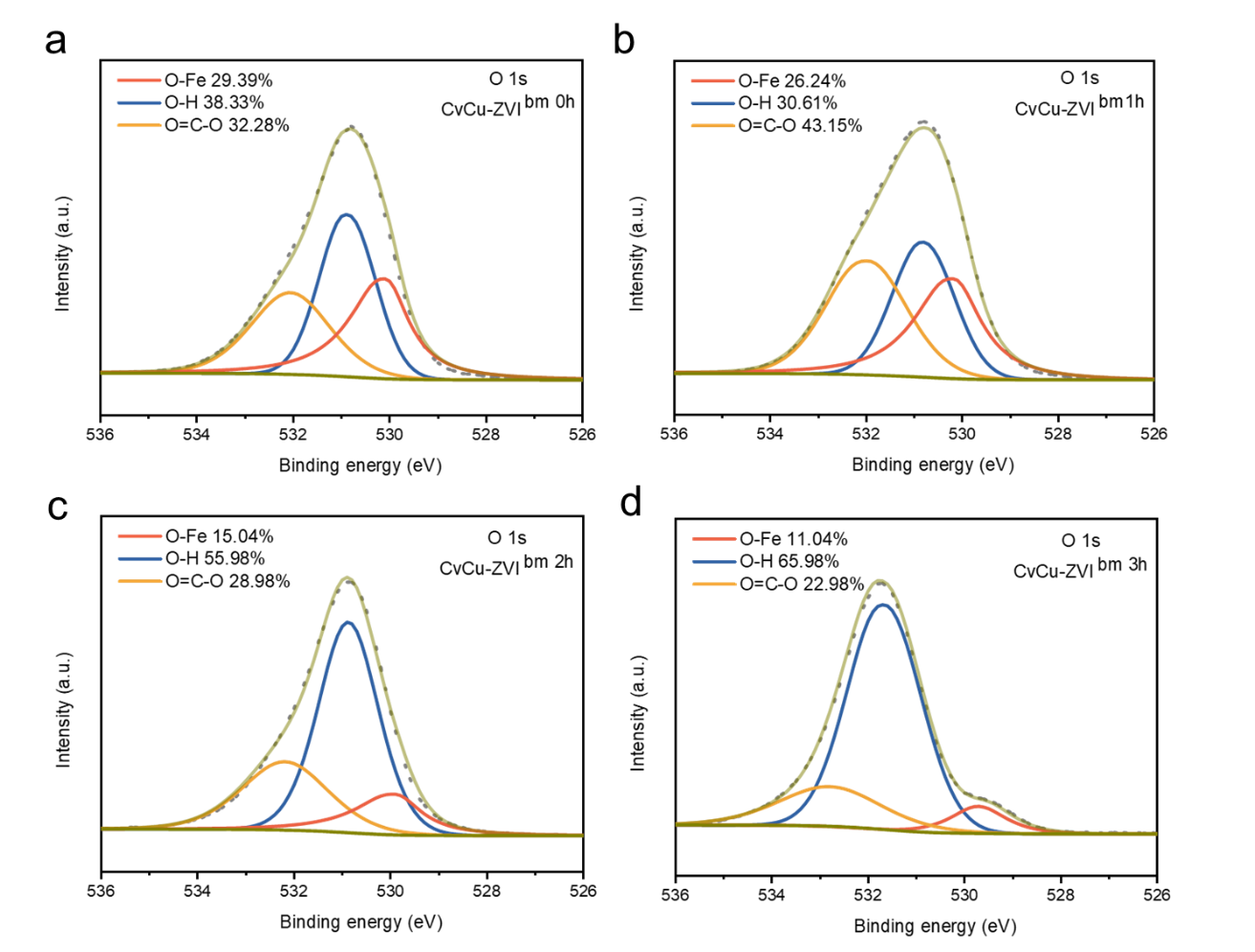
Figure S12.** High-resolution XPS O 1s spectra of CvCu-ZVI prepared by (a) 0 h, (b) 1 h, (c) 2 h, and (d) 3 h of milling time.


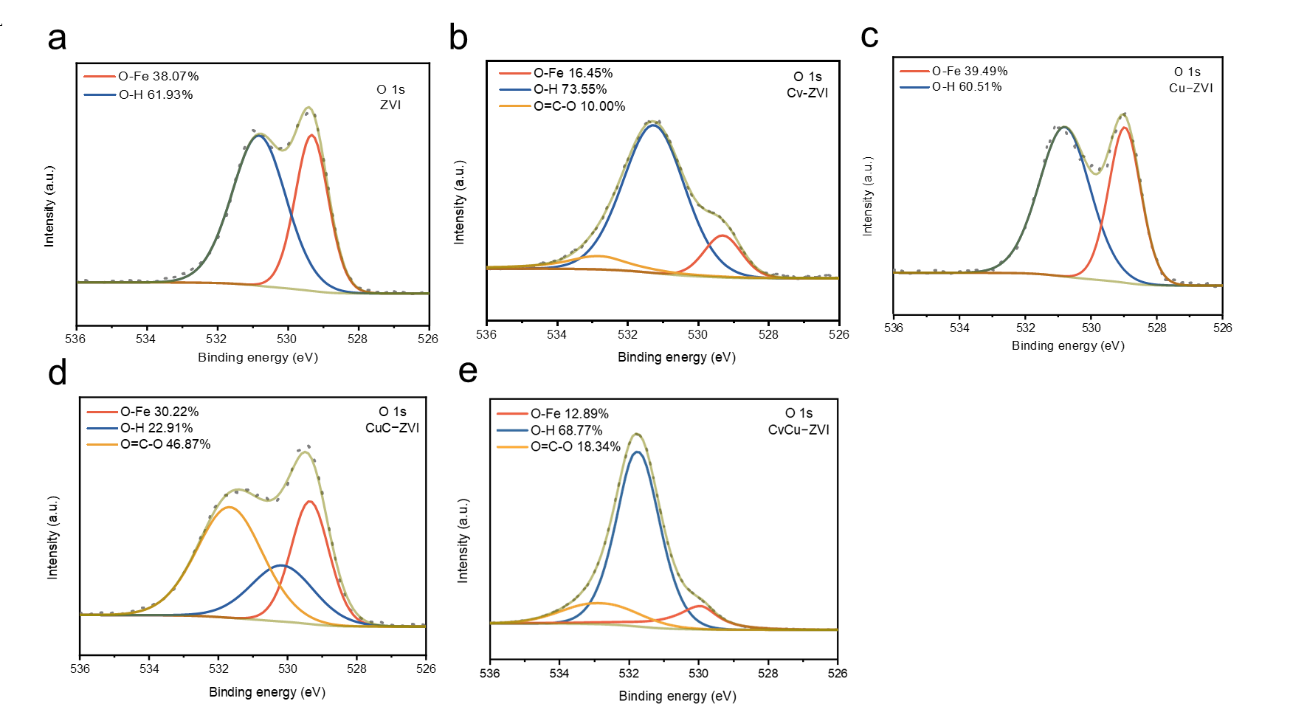


**Figure S13.** High-resolution XPS O 1s spectra of (a) ZVI, (b) Cv-ZVI, (c) Cu-ZVI, (d) CCu-ZVI, and (e) CvCu-ZVI.

**
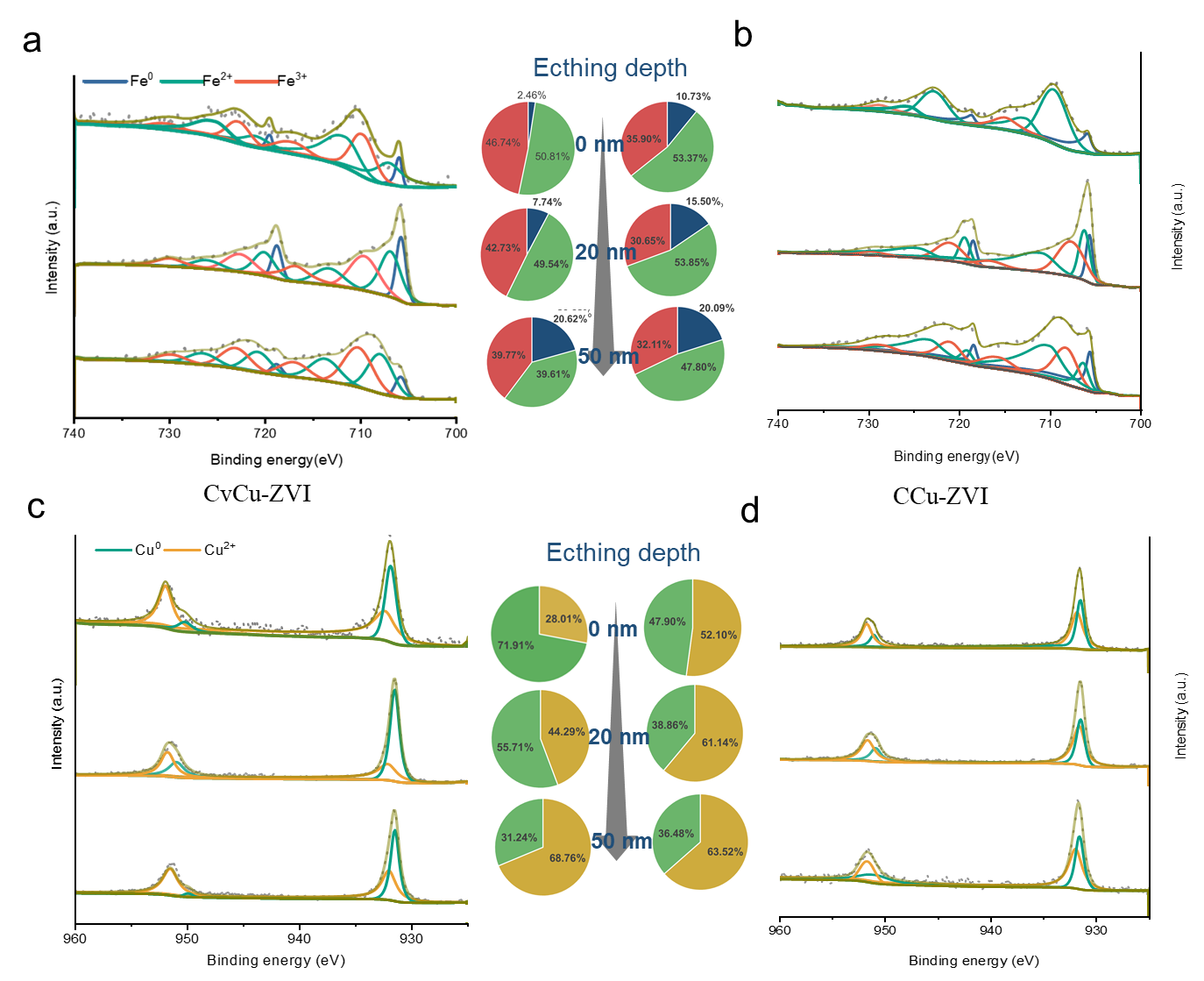
**

**
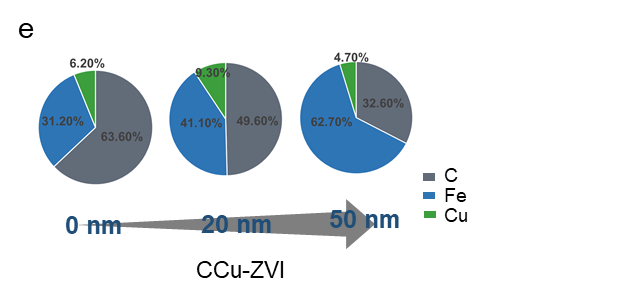
**

**Figure S14.** Fe 2p XPS depth profile of (a) CvCu-ZVI and (b) CCu-ZVI. Cu 2p XPS depth profile of (c) CvCu-ZVI and (d) CCu-ZVI. (e) Content analysis of the elements in the XPS depth profile of CCu-ZVI.

**
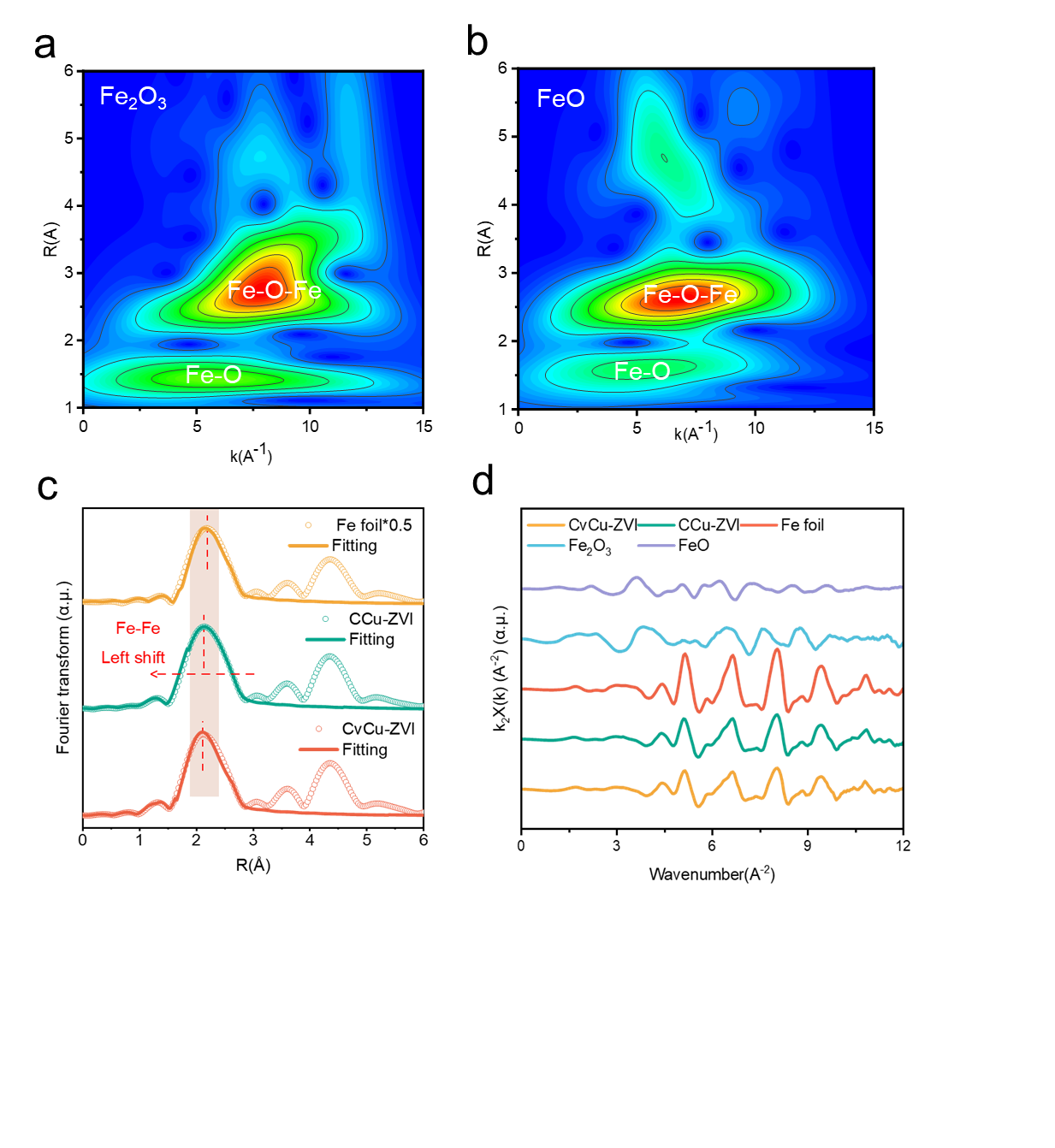
**

**Figure S15.** WT-EXAFS plots of (a) Fe_2_O_3_ and (b) FeO. (c) The fitting results of CvCu-ZVI, CCu-ZVI, and Fe foil in R space. (d) K^2^ space spectra of CvCu-ZVI, CCu-ZVI, Fe foil, Fe_2_O_3_, and FeO at the Fe *K*-edge.

**Figure S16.** High-resolution XPS Fe 2p spectra of (a) ZVI, (b) Cv-ZVI, and (c) Cu-ZVI. (d) XPS Fe 2p content analysis for different materials.

Note: The Fe^0^ content on the surface of Cv-ZVI and CuCv-ZVI was significantly larger compared to that in ZVI and Cu-ZVI, suggesting that the modification of Cv regenerated surface Fe^0^, which may be the result of volatilization of O prompting the self-reduction of the positively oxidized-valent Fe species by using electrons released from the Fe nucleus.^[13]^ The modification of Cv resulted in a higher Fe^0^ content on the surface of CvCu-ZVI (18.52%) than that of CCu-ZVI (15.21%), suggesting that the formation of Cv effectively promoted the conversion of FeO_x_ to Fe^0^. In addition, compared to Cv-ZVI (10.99%), the Fe^0^ content in Cu-ZVI increased from 7.83% in ZVI to 15.25%, indicating that Cu embedded in the lattice significantly induced the reduction of FeO_x_ during ball milling, thereby favoring the formation of Fe^0^.

**Figure S17**. Cu LMM AES spectra of (a) Cu-ZVI, CCu-ZVI and CvCu-ZVI. (c) Corresponding Cu LMM AES content analysis. High-resolution XPS Cu 2p spectra of (b) Cu-ZVI , CCu-ZVI and CvCu-ZVI, and (d) Cu 2p content analysis.

Note: According to the Cu LMM AES results,^[14]^ the content of Cu(II) in CvCu-ZVI increased significantly from 36.59% in CC-ZVI to 55.89%, accompanied by a decrease in Cu(0) from 24.35% to 19.24% and a reduction in Cu(I) from 39.06% to 24.87%. These changes indicated that the formation of Cv promoted electron transfer from Cu^0^ to FeO_x_. This observation was further corroborated by high-resolution Cu 2p XPS analysis. Interestingly, as the Cu^0^ content decreased, a new peak attributed to Cu(I) (953.7 eV) appeared in CCu-ZVI, while a new peak attributed to Cu(II) (940.1 eV) appeared in CvCu-ZVI, indicating that Cv formation facilitated electron transfer from Cu^0^ to FeO_x_.


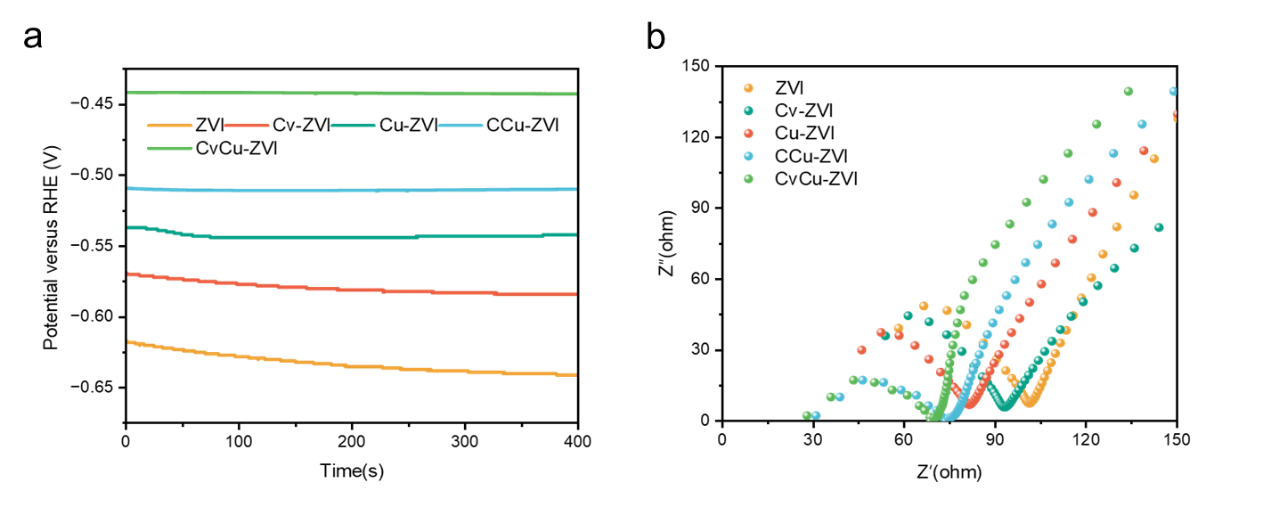


**Figure S18**. (a) OCP curves of different materials and (b) EIS Nyquist plots of different materials.

.


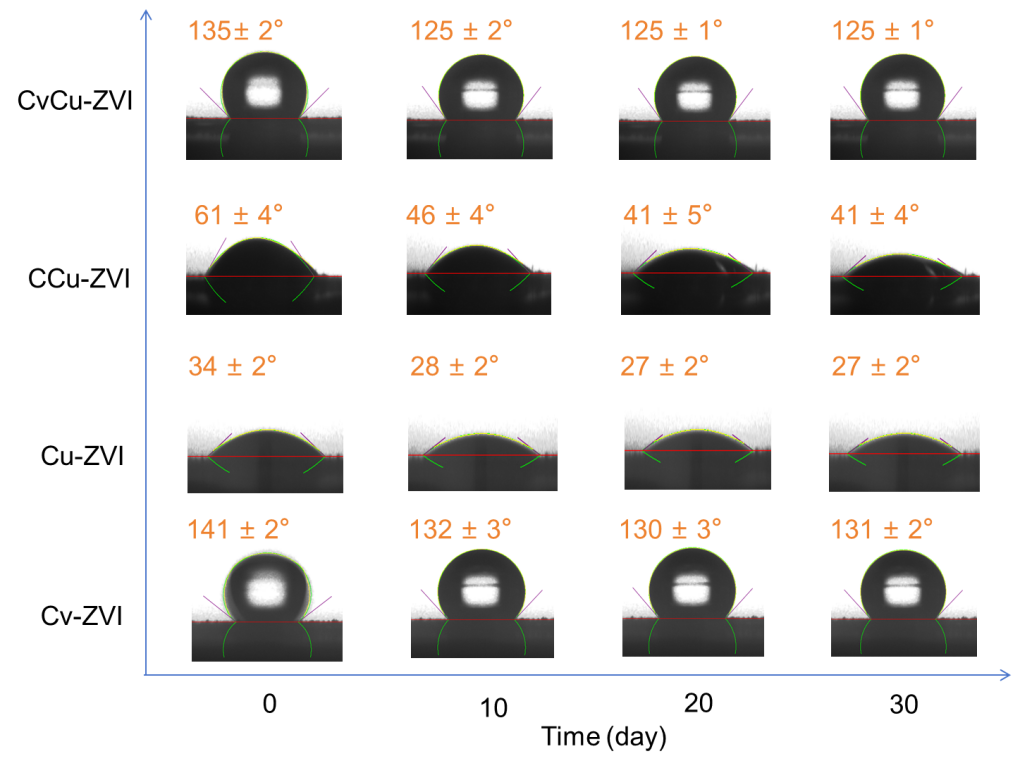


**Figure S19**. WCA measurements of different materials over a 30-day period.

##
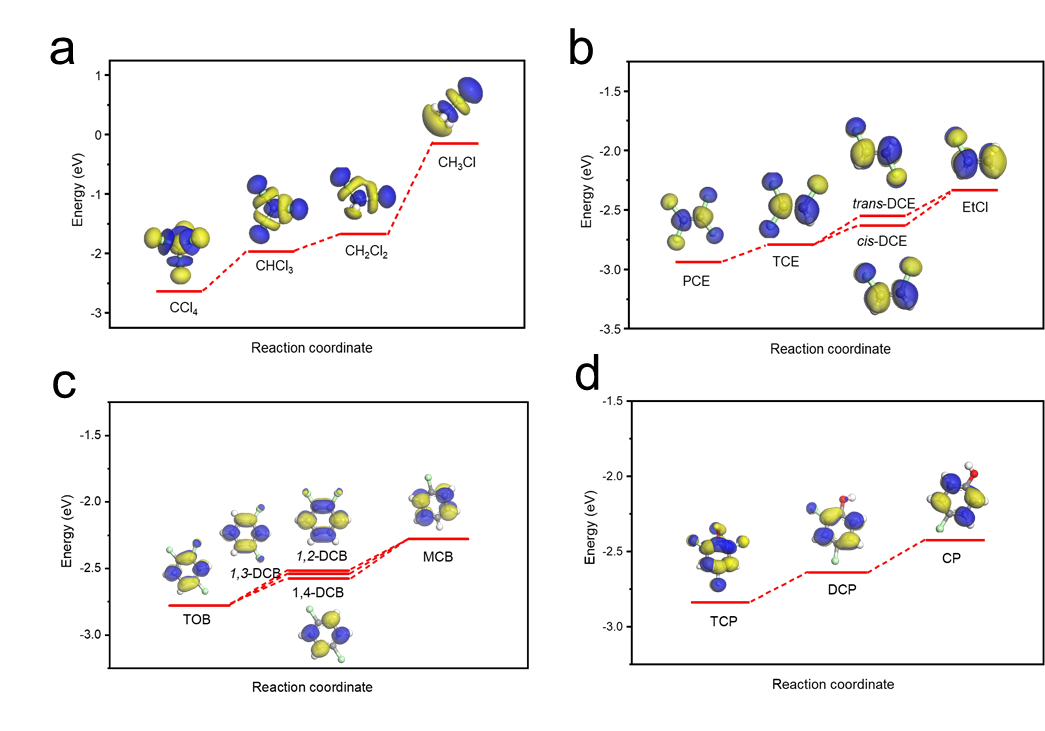


## Figure S20. LUMO diagrams of intermediates and products of CvCu-ZVI for (a) CCl_4_, (b) PCE, (c) TOB, and (d) TCP removal.

Note: Indeed, the structure of PCOs was significantly correlated with the dechlorination activity, which we analyzed in detail using DFT calculations. The lowest unoccupied molecular orbital (LUMO) was defined as the frontier molecular orbital where ET occurs, typically located on the C-Cl bond of PCOs, and the descriptor *E*_LUMO_ can reflect the ease of dechlorination of ET-based PCOs. Compared with the reactants, the *E*_LUMO_ of the dechlorination products increased as the chlorine content decreased, suggesting that the dechlorination of low-chlorine products became more challenging.^[15]^ Particularly, the *E*_LUMO_ of CCl_4_ and its dechlorinated intermediates were significantly higher, with the *E*_LUMO_ of CH_3_Cl being only -0.15 eV, emphasizing the significant challenge of achieving complete dechlorination of CCl_4_.

**
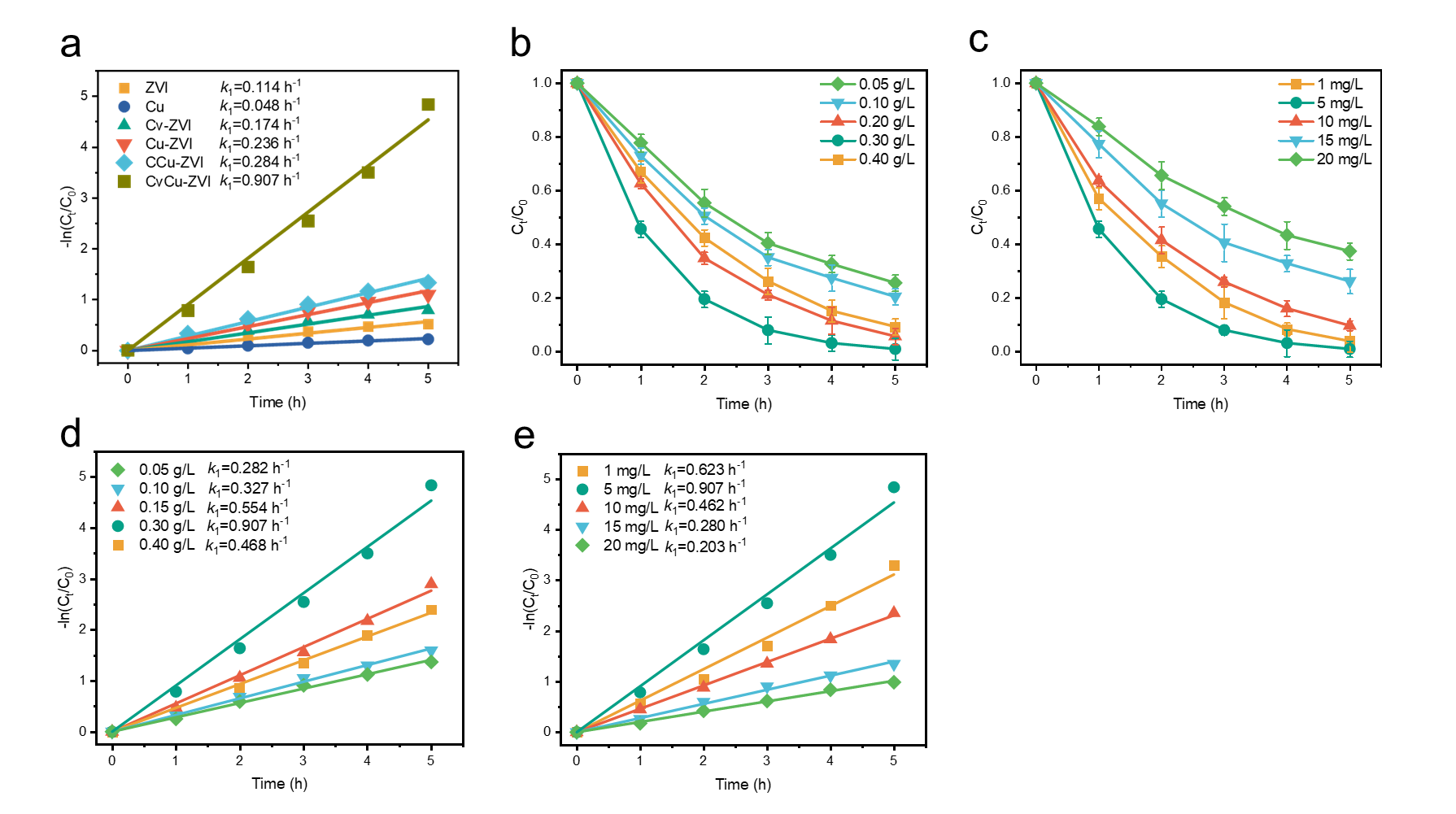
**

**Figure S21.** (a) The efficiency of different materials for the corresponding pseudo-first rate constant (*k*_1_). Effects of (b, d) sample dosage and (c, e) initial concentration of CCl_4_ on CCl_4_ removal by CvCu-ZVI and the corresponding pseudo-first order kinetic constants (*k*_1_). (Reaction conditions: [CvCu-ZVI]_0_= 0.30 g L^-1^, [CCl_4_]_0_= 5.0 mg L^-1^, T= 298 K.)

Note: The reduction efficiency of CCl_4_ gradually increased when the dosage of ZVI was varied from 0.05 to 0.3 g L^-1^, but no significant further improvement was observed beyond this dosage. Consequently, the optimal dosage of CvCu-ZVI was set at 0.3 g L^-1^. In addition, a higher CCl_4_ concentration did not affect the reduction efficiency of CvCu-ZVI, with even 10 mg L^-1^ CCl_4_ being removed by 90.49% within 5 h.

**
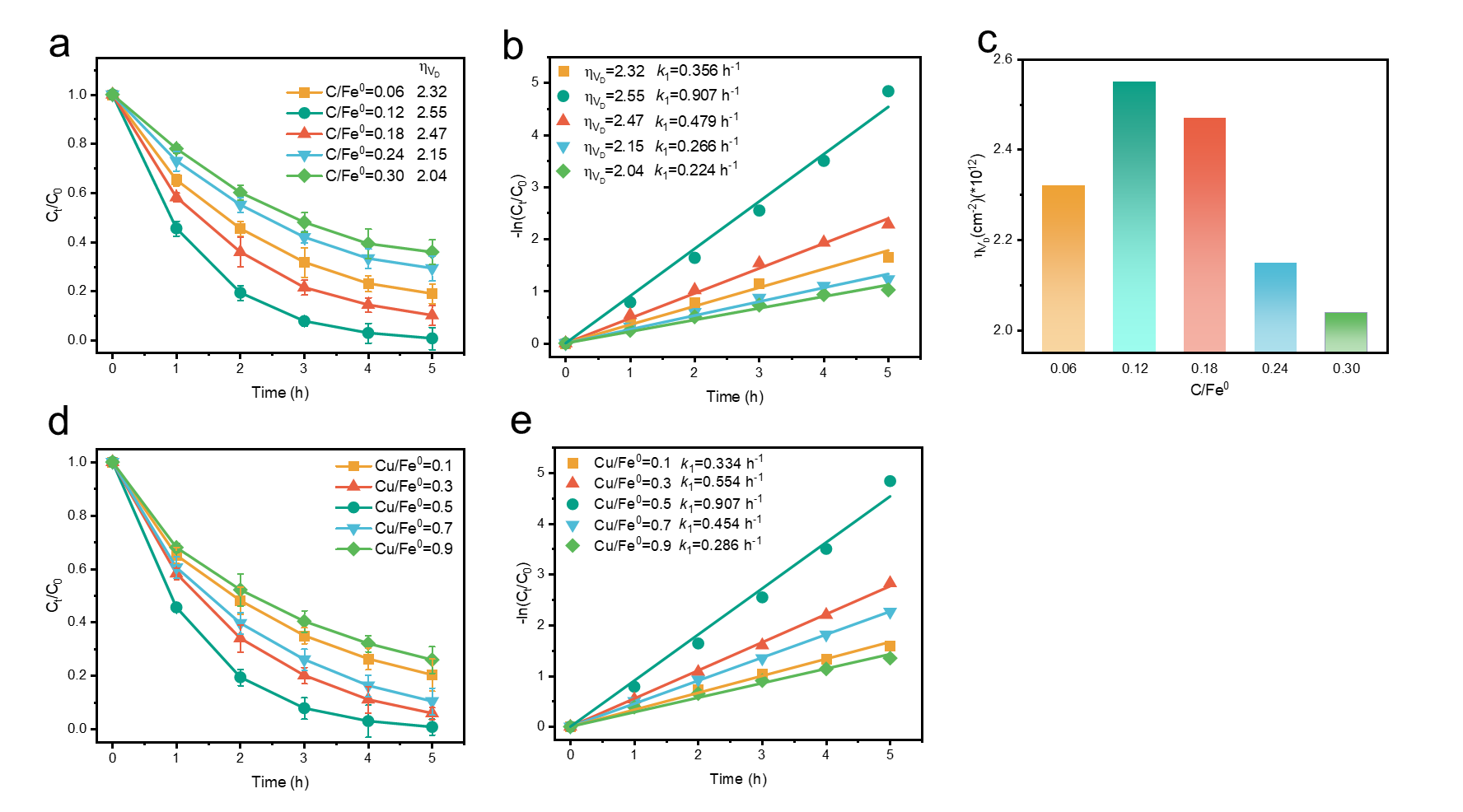
Figure S22.** Effects of (a, b) different defect density (ƞV_D_) and (d, e) Cu content on the removal of CCl_4_ by CvCu-ZVI and the corresponding pseudo-first order kinetic constants (*k*_1_). (c) ƞV_D_ of CvCu-ZVI with different C/Fe^0^ ratios. (Reaction conditions: [CvCu-ZVI]_0_= 0.30 g L^-1^, [CCl_4_]_0_= 5.0 mg L^-1^, T= 298 K.)

Note: The CCl_4_ removal efficiency of CvCu-ZVI was evaluated at various C/Fe^0^ and Cu/Fe^0^ molar ratios. At C/Fe^0^ ratios of 0.06 to 0.30, corresponding ƞV_D_ ranged from 2.32 cm^-2^ to 2.04 cm^-2^, with CCl_4_ removal efficiencies of 81.99% to 64.07%. The peak ƞV_D_ (2.55 cm^-2^) yielded a *k*_1_ of 0.91 h^-1^, significantly higher than other materials. These results indicated that the presence of carbon vacancies significantly enhanced the material’s performance in neutral conditions for CCl_4_ removal. At Cu/Fe^0^ ratios of 0.1 to 0.9, CCl_4_ removal efficiencies ranged from 79.68% to 74.07%, showing an initial increase followed by a decline due to excessive Cu occupying active sites. Overall, the contents of Cv and lattice Cu were key for efficient CCl_4_ dechlorination.

**
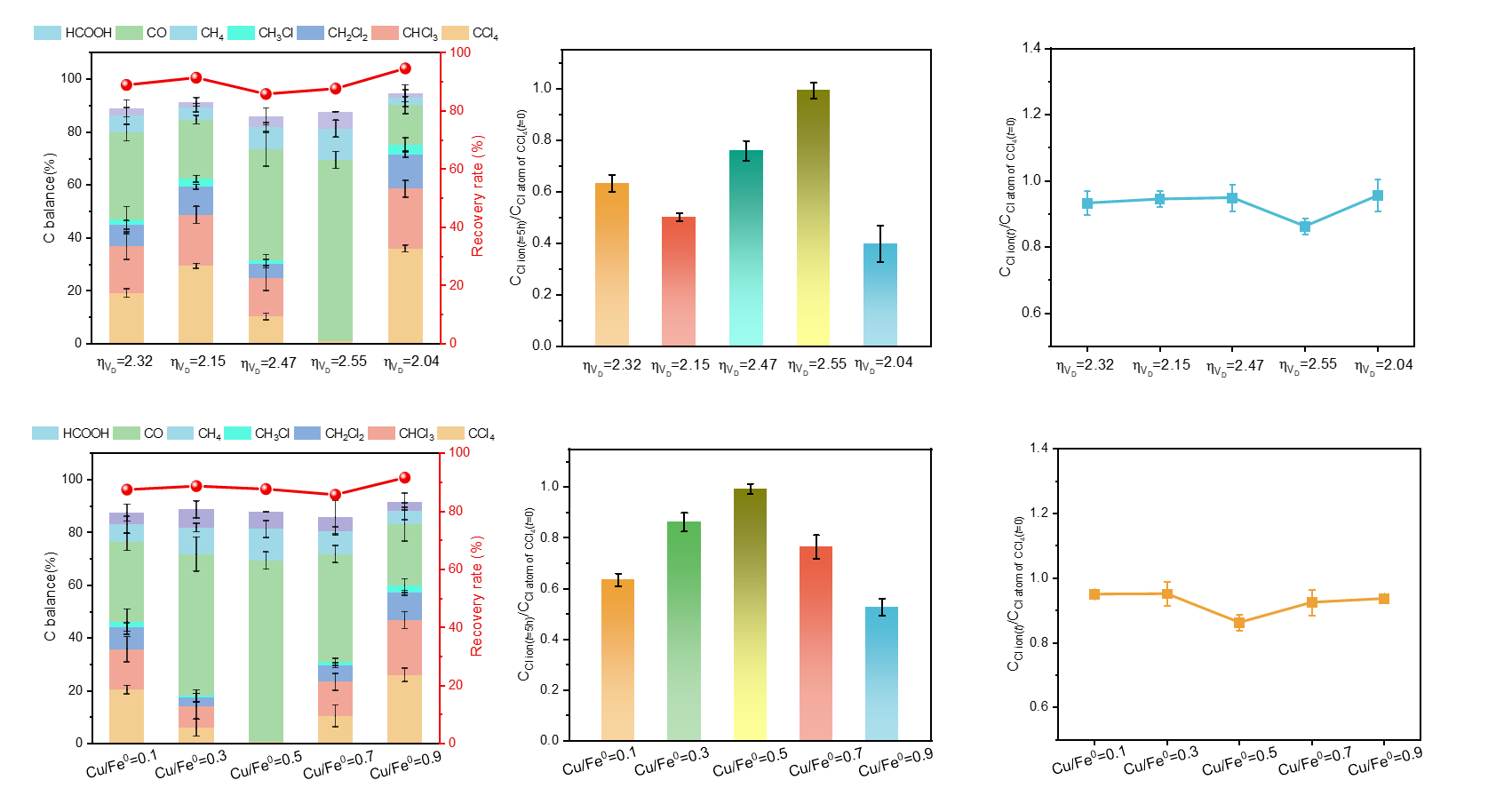
**

**Figure S23.** (a, d) Product analysis, (b, e) concentration ratios of Cl^-^ ions in solution and Cl atoms in original CCl_4_ during the 5-h reation and (c, f) mass balance of chlorine elements of chlorine elements in CCl_4_ removal by CvCu-ZVI with different ƞV_D_ and Cu content. (Reaction conditions: [CvCu-ZVI]_0_= 0.30 g L^-1^, [CCl_4_]_0_= 5.0 mg L^-1^, T= 298 K.)

Note: In addition to the intermediates of trichloromethane, dichloromethane, and monochloromethane, which were sequentially dechlorinated and converted, the final products were detected to be primarily methane with small amounts of CO and HCOOH. Where CO formation may be the result of carbonylation and dehydrogenation of the dechlorination intermediates (CCl_4_ → ·CCl_3_ → ·CCl_2_ → ·CHO → CO), and HCOOH may be formed by hydroxylation of some of the intermediates by water or by hydroxylation of oxygen-containing functional groups on ZVI for dechlorination.


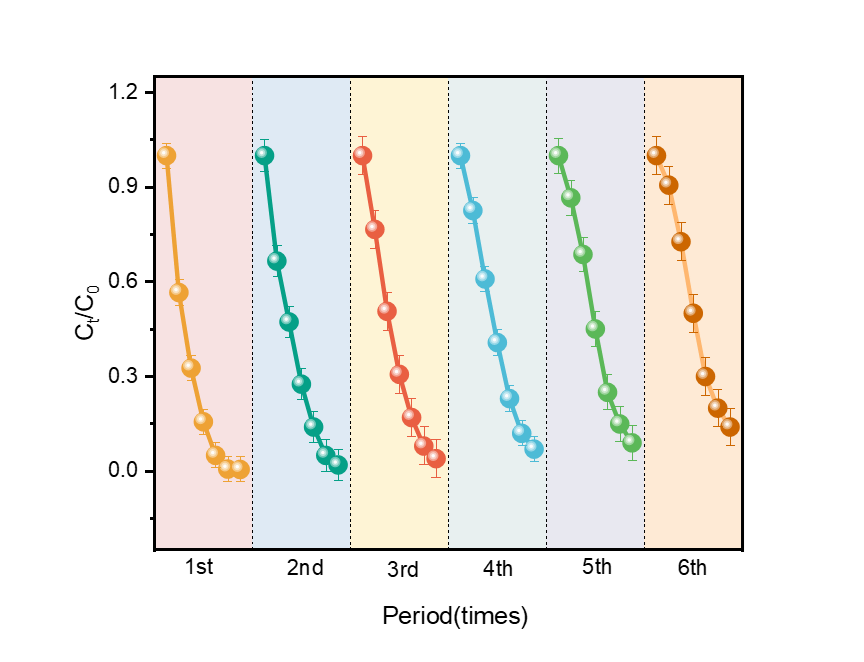


**Figure S24**. The removal efficiency of CCl_4_ by CvCu-ZVI across six cycles and subsequent recovery treatments. (Reaction conditions: [CvCu-ZVI]_0_= 0.30 g L^-1^, [CCl_4_]_0_= 5.0 mg L^-1^, T= 298 K.)

**Figure S25.** In-suit FTIR spectrum of CvCu-ZVI for CCl_4_ removal. (Reaction conditions: [CvCu-ZVI]_0_= 0.30 g L^-1^, [CCl_4_]_0_= 5.0 mg L^-1^, T= 298 K.)

Note: In-situ FTIR was employed to monitor reaction intermediates in real-time, which was crucial for elucidating the reaction mechanism. Upward peaks indicated the consumption of reactants, while downward peaks reflected the accumulation of products.^[11]^ Specifically, a significant upward peak was observed at 816 cm^-1^, corresponding to C–Cl. Significant downward peaks were observed at 1150 cm^-1^ and 1445 cm^-1^, corresponding to C–H and C–H_2_, respectively. These observations suggested the cleavage of the C–Cl bond and the formation of new -CH and -CH_2_ bonds. Additionally, two downward peaks were observed at 1810 cm^-1^ and 3370 cm^-1^, corresponding to -OH^-^. These peaks likely resulted from the dissociation of water molecules into H*, further confirming the generation of H*.


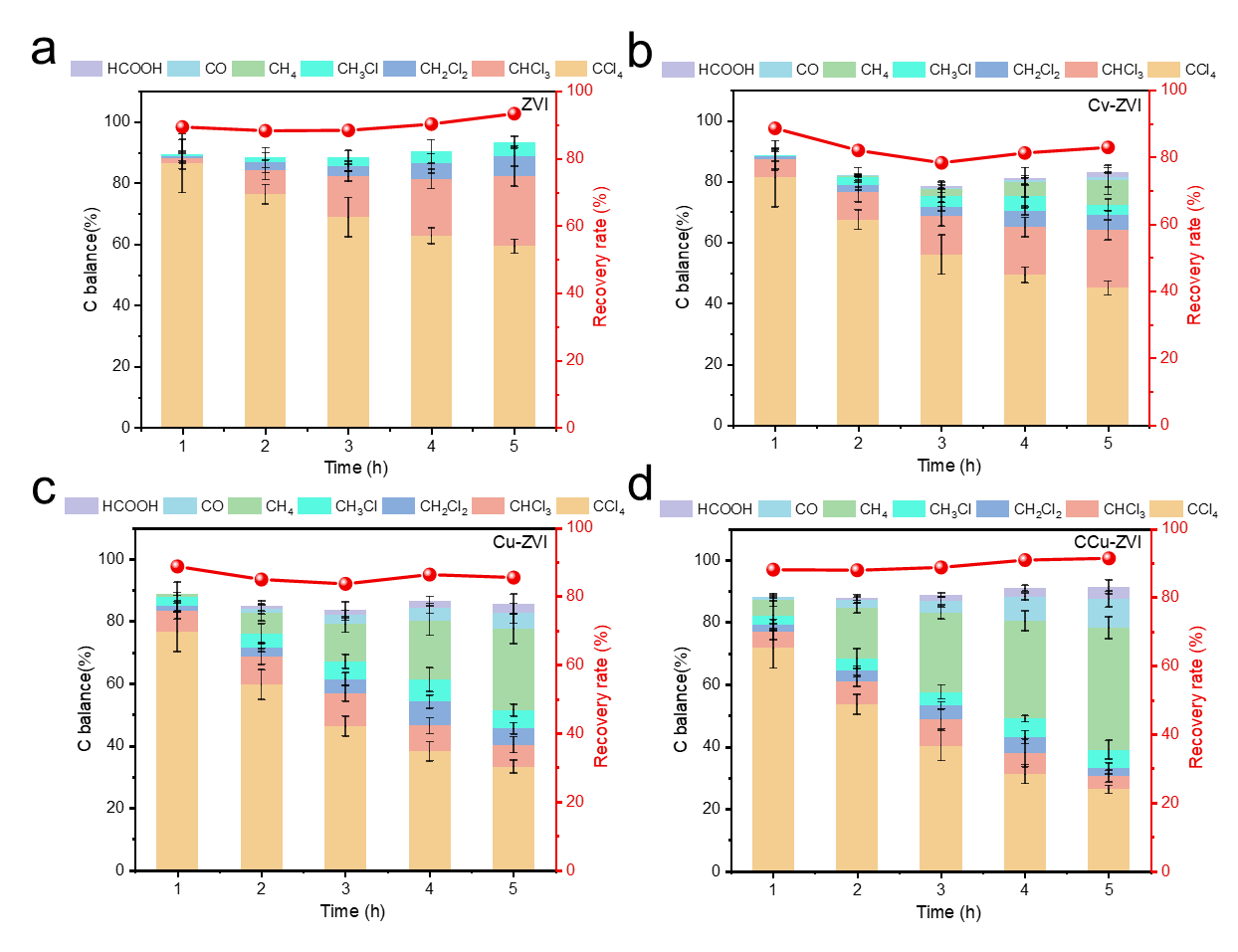


**Figure S26**. Product analysis of (a) ZVI, (b) Cv-ZVI, (c) Cu-ZVI, and (d) CCu-ZVI for CCl_4_ removal. (Reaction conditions: [Materials]_0_= 0.30 g L^-1^, [CCl_4_]_0_= 5.0 mg L^-1^, T= 298 K.)


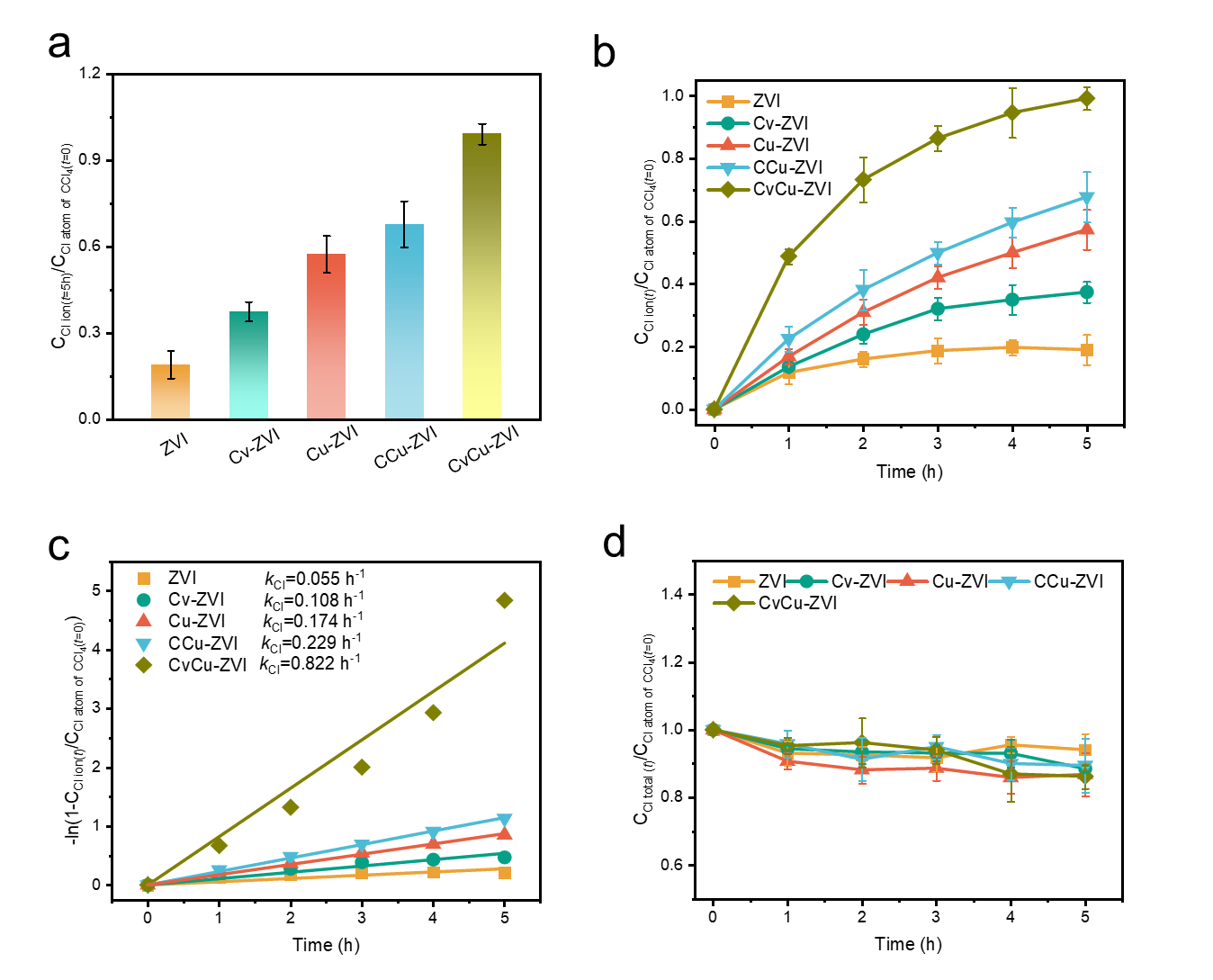


**Figure S27**. (a) Concentration ratios of Cl^-^ ions in solution and Cl atoms in original CCl_4_ during the 5-h reation process with different materials. (b) Concentration ratios of Cl^-^ ions in solution and Cl atoms in original CCl_4_ removal by different materials. (c) Dechlorination rate constants for the removal of CCl_4_ by different materials. (d) Mass balance of chlorine elements, including CHCl_3_, CH_2_Cl_2_, CH_3_Cl, and the released Cl^−^ ions. (Reaction conditions: [Materials]_0_= 0.30 g L^-1^, [CCl_4_]_0_=5.0 mg L^-1^, T= 298 K.)


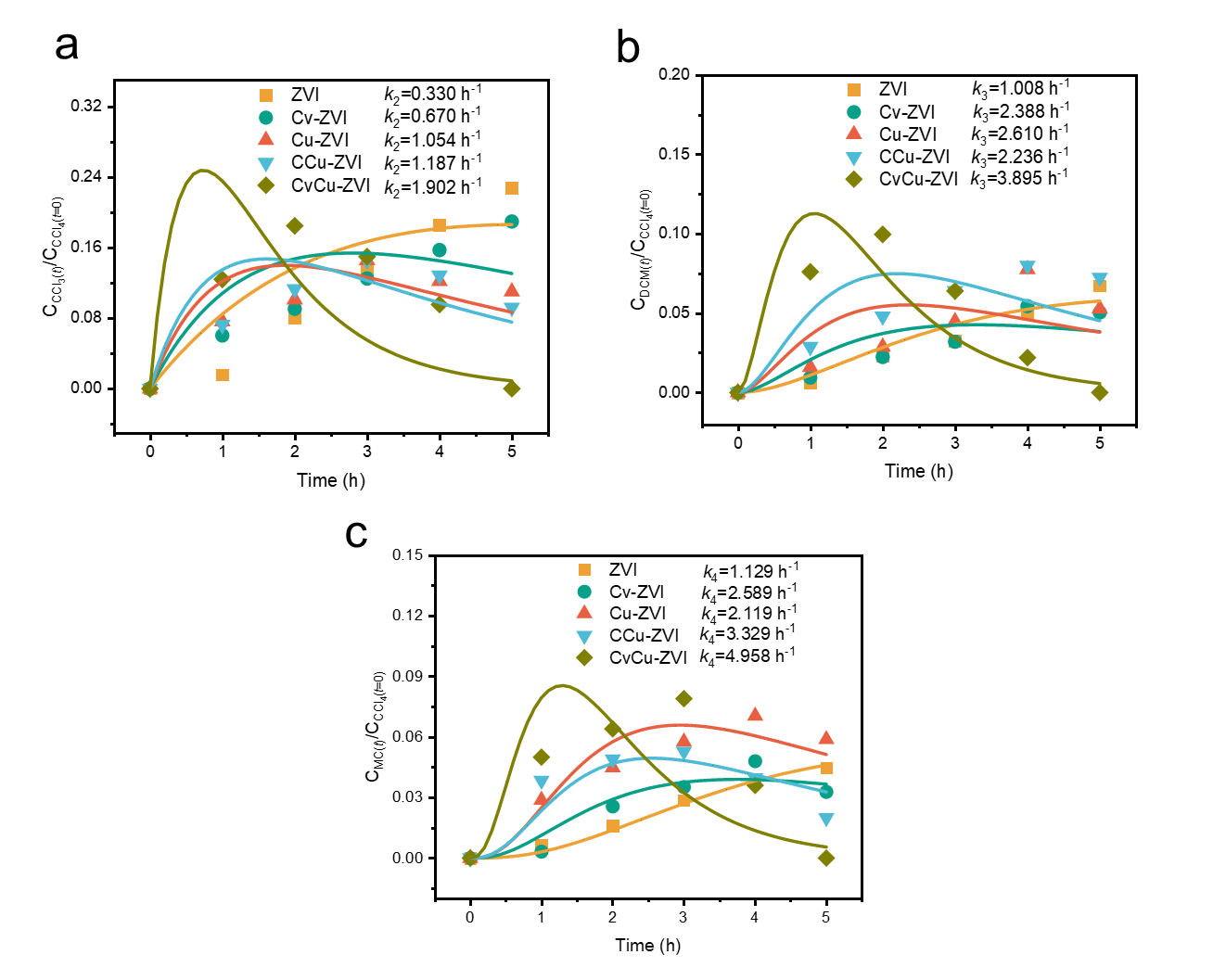


**Figure S28**. Apparent rate constants of (a) CHCl_3_ conversion (*k*_2_), (b) CH_2_Cl_2_ conversion (*k*_3_), and (c) CH_3_Cl conversion (*k*_4_).

**Figure S29**. Dechlorination rate constants and actual quantities of Fe^0^ for CCl_4_ removal by different materials. (Reaction conditions: [Materials]_0_= 0.30 g L^-1^, [CCl_4_]_0_= 5.0 mg L^-1^, pH= 7.0, T=298K, Error bars indicate standard deviations obtained from thrice independent measurements).

##
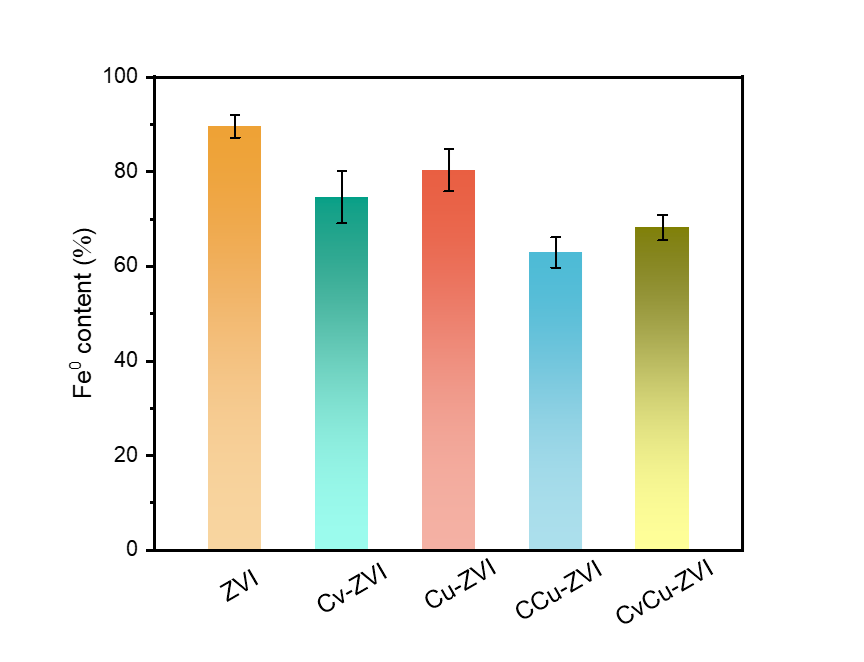


## Figure S30. The Fe^0^ content of different materials.


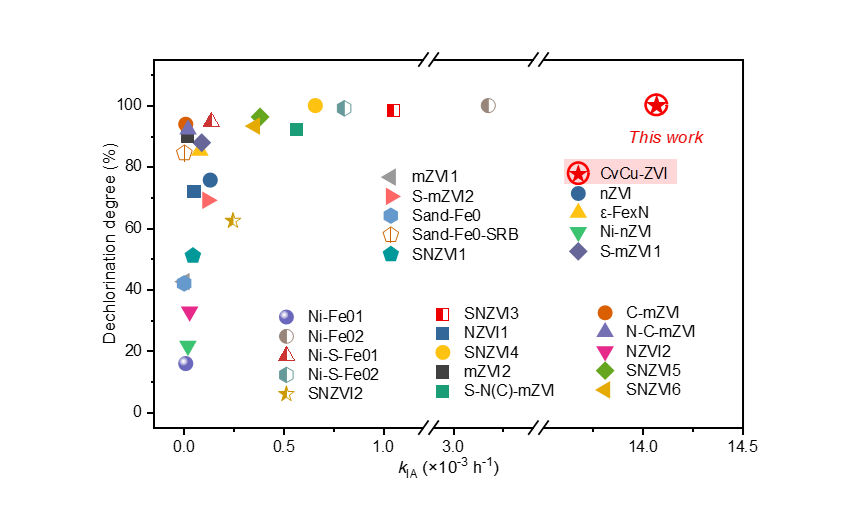


**Figure S31.** Comparison of the published intrinsic activity rate constants (*k*_IA_) and dechlorination degree of Fe^0^-based materials for TCE removal (The related information was presented in Table S8).

**
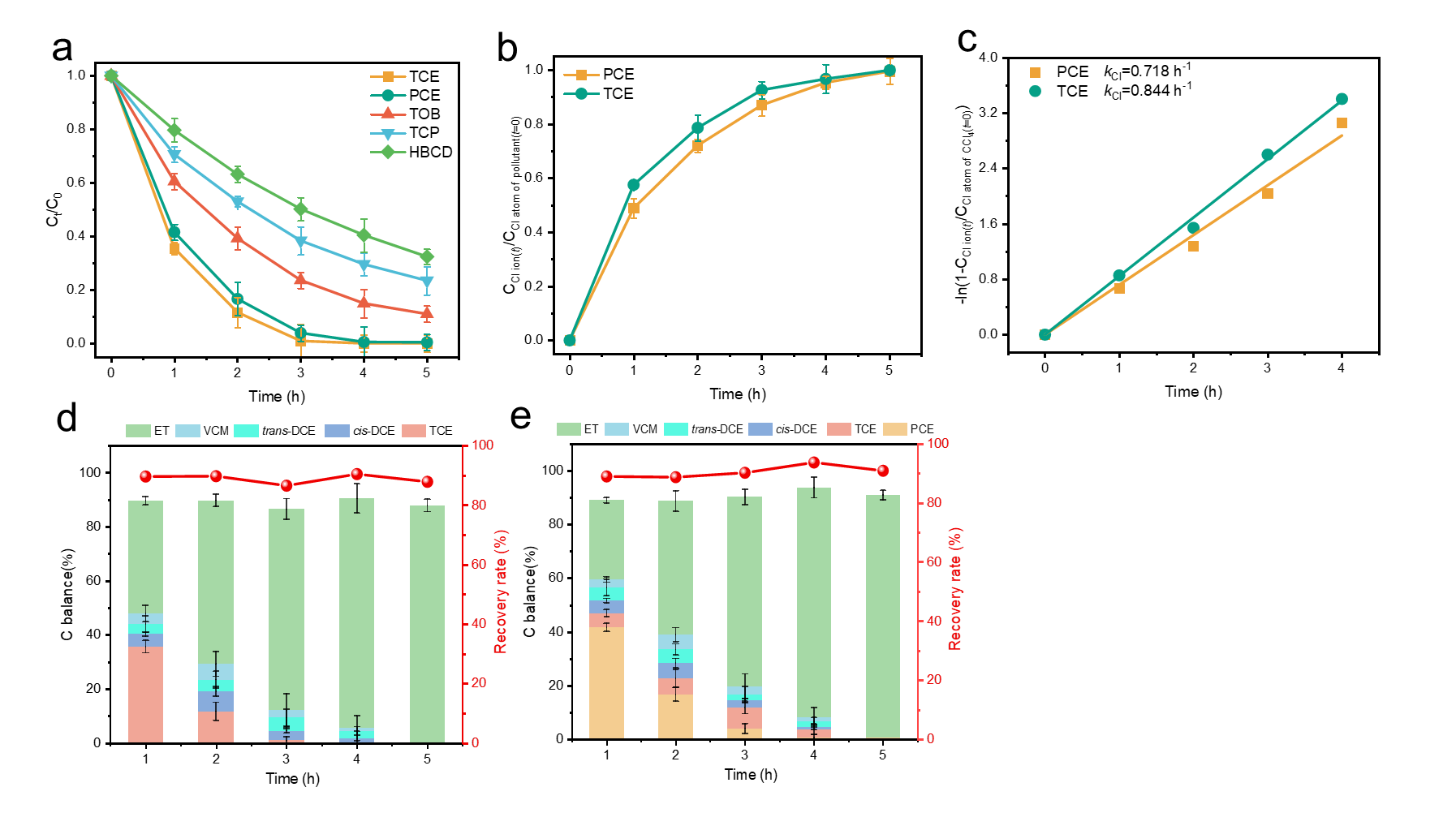
**

**Figure S32.** (a) The efficiency of CvCu-ZVI for different pollutions removal. (b) Concentration ratios of Cl^-^ ions in solution and Cl atoms in pollutions during the 5-h reation, (c) dechlorination rate constants for the removal of pollutions and (d, e) product analysis of CvCu-ZVI for different pollutions removal. (Reaction conditions: [CvCu-ZVI]_0_= 0.30 g L^-1^, [TCE]_0_=[PCE]_0_= [TOB]_0_= [TCP]_0_= [HBCD]_0_= 5.0 mg L^-1^, T= 298 K.)

**
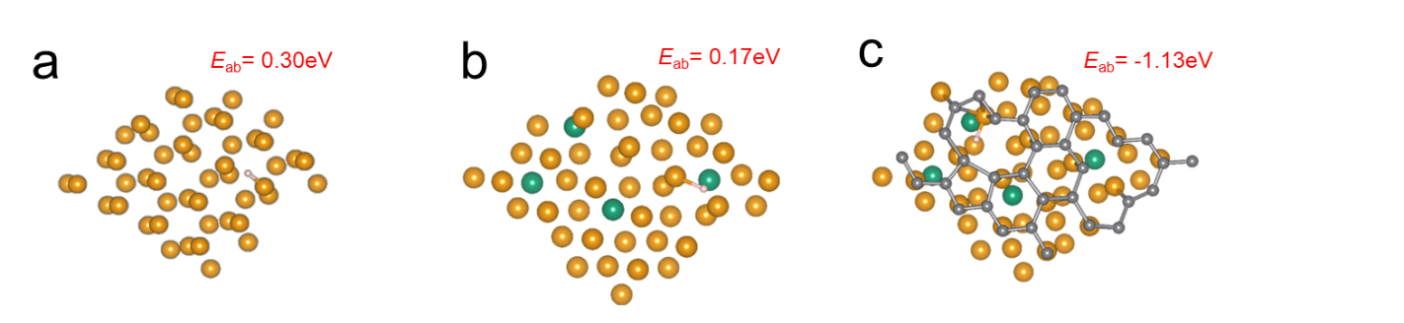
**

**Figure S33**. The model of H^*^ adsorbed on (a) ZVI, (b) Cu-ZVI, and (c) CvCu-ZVI.

**
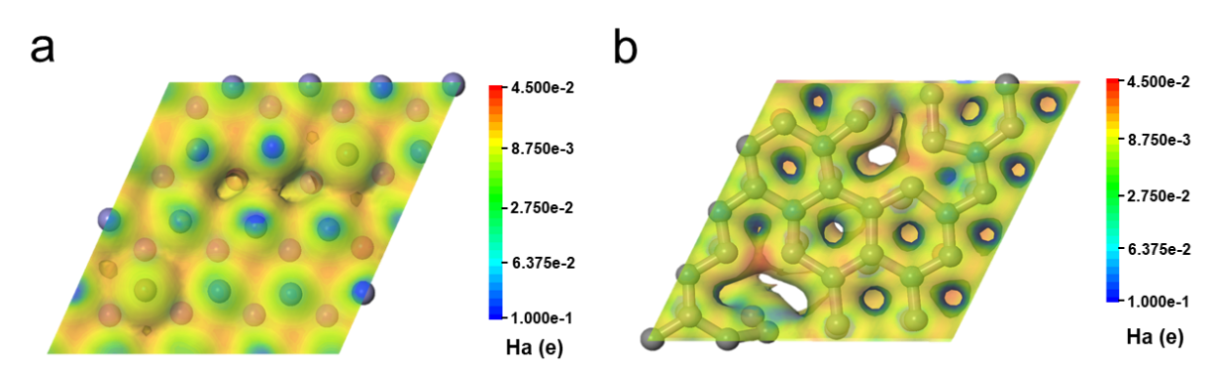
**

**Figure S34**. Electrostatic potential diagrams of (a) Cu-ZVI and (b) Cv-ZVI.


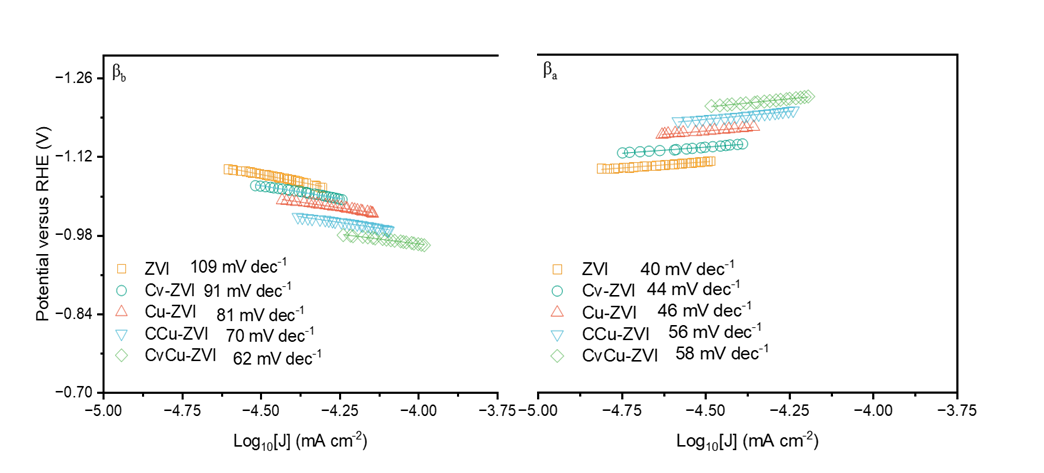


## Figure S35. The anodic Tafel slope (*β*_a_) and cathodic Tafel slope (*β*_b_) of different materials.


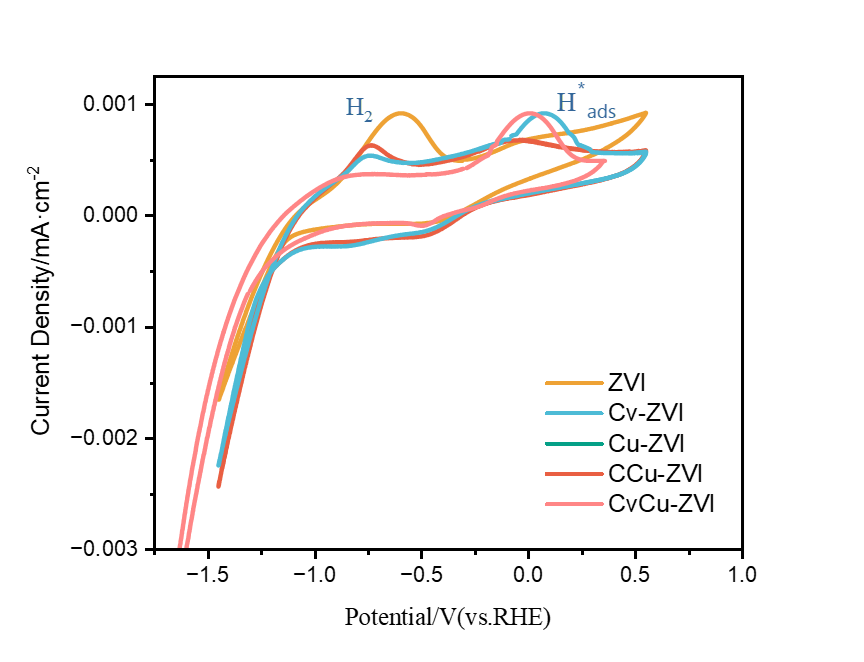


## Figure S36. The cyclic voltammetry curves of different materials.

**
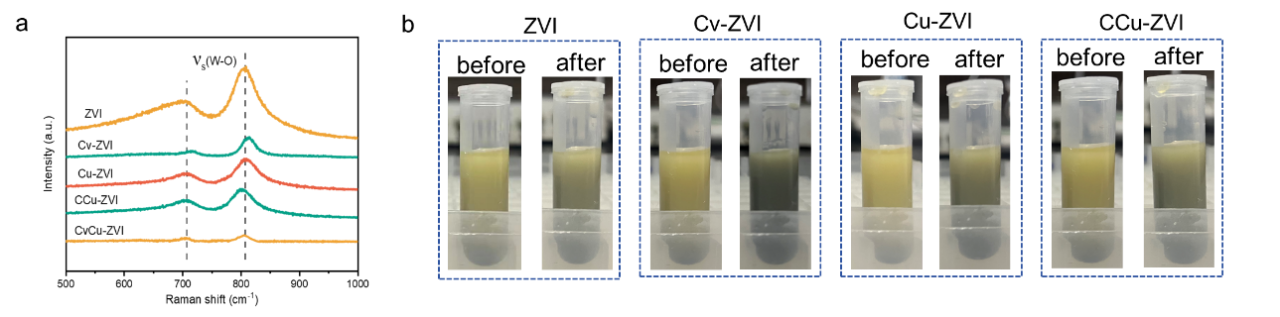
**

**Figure S37**. (a) Raman spectra of different materials in combination with WO_3_. (b) Color change of WO_3_ on different materials by hydrogen spillover effect.


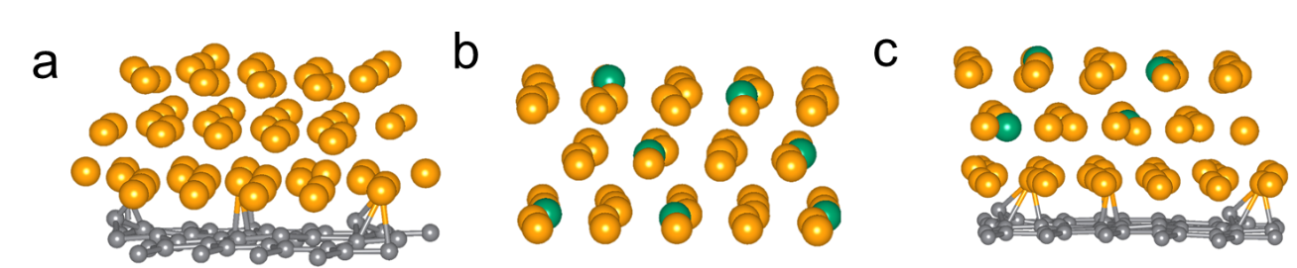


**Figure S38**. Optimal configurations of (a) Cv-ZVI, (b) Cu-ZVI and (c) CvCu-ZVI. (Elements corresponding to atoms of different colors: yellow =Fe, gray=C,green=Cu.)


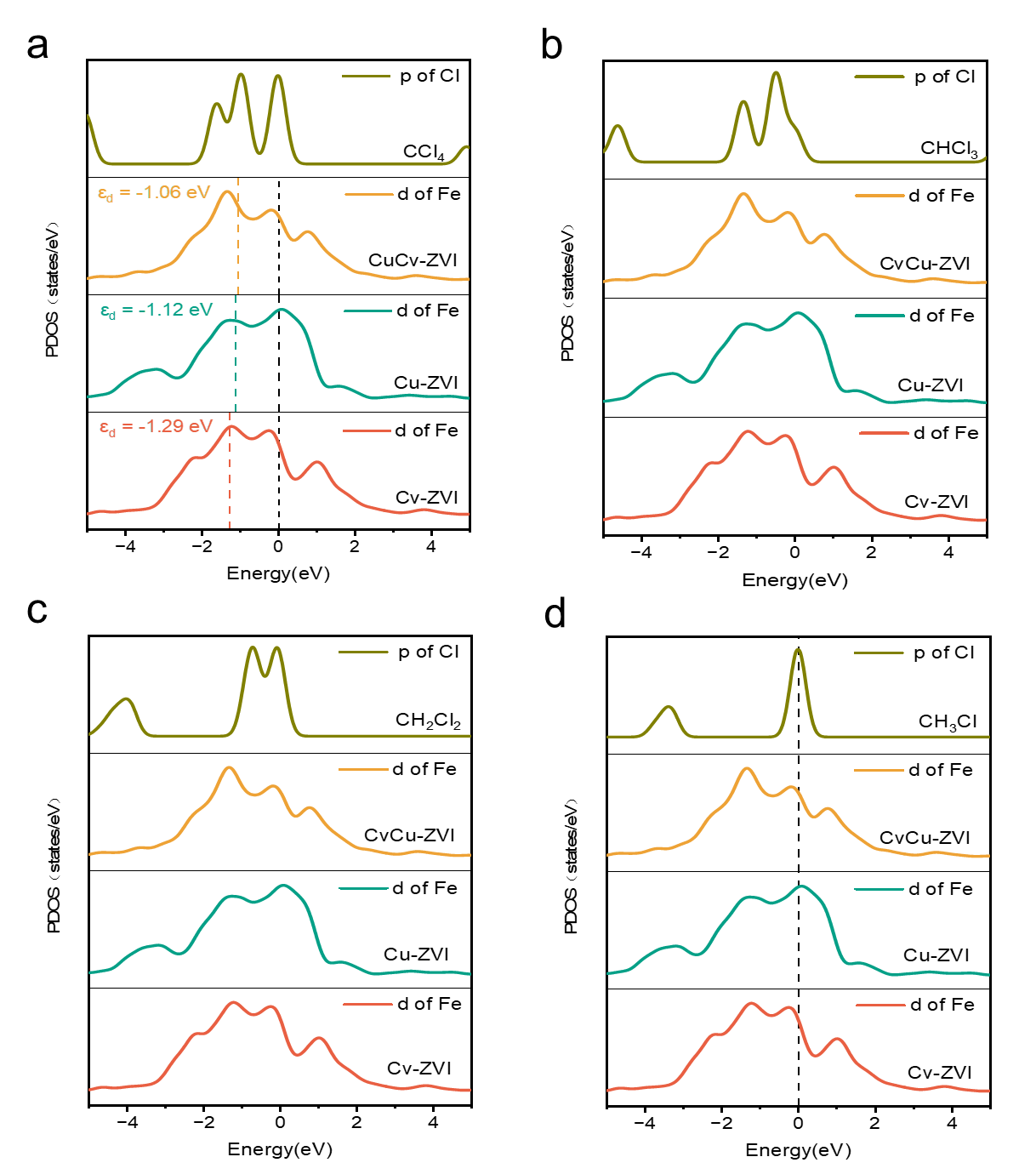


**Figure S39.** The partial fractional density of states analysis of the interaction between the Fe 3d orbitals of different materials and the Cl 2p orbitals of (a) CCl_4_, (b) CHCl_3_, (c) CH_2_Cl_2_, and (d) CH_3_Cl.

**
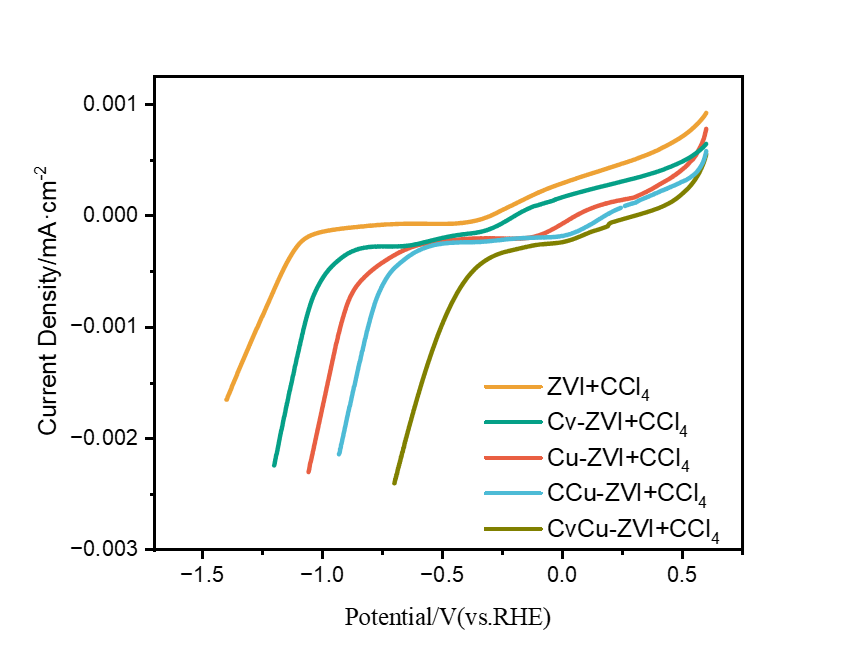
**

**Figure S40**. LSV curves of different materials after the addition of CCl_4_. (Reaction conditions: [Materials]_0_= 0.30 g L^-1^, [CCl_4_]_0_= 5.0 mg L^-1^, T= 298 K.)

**
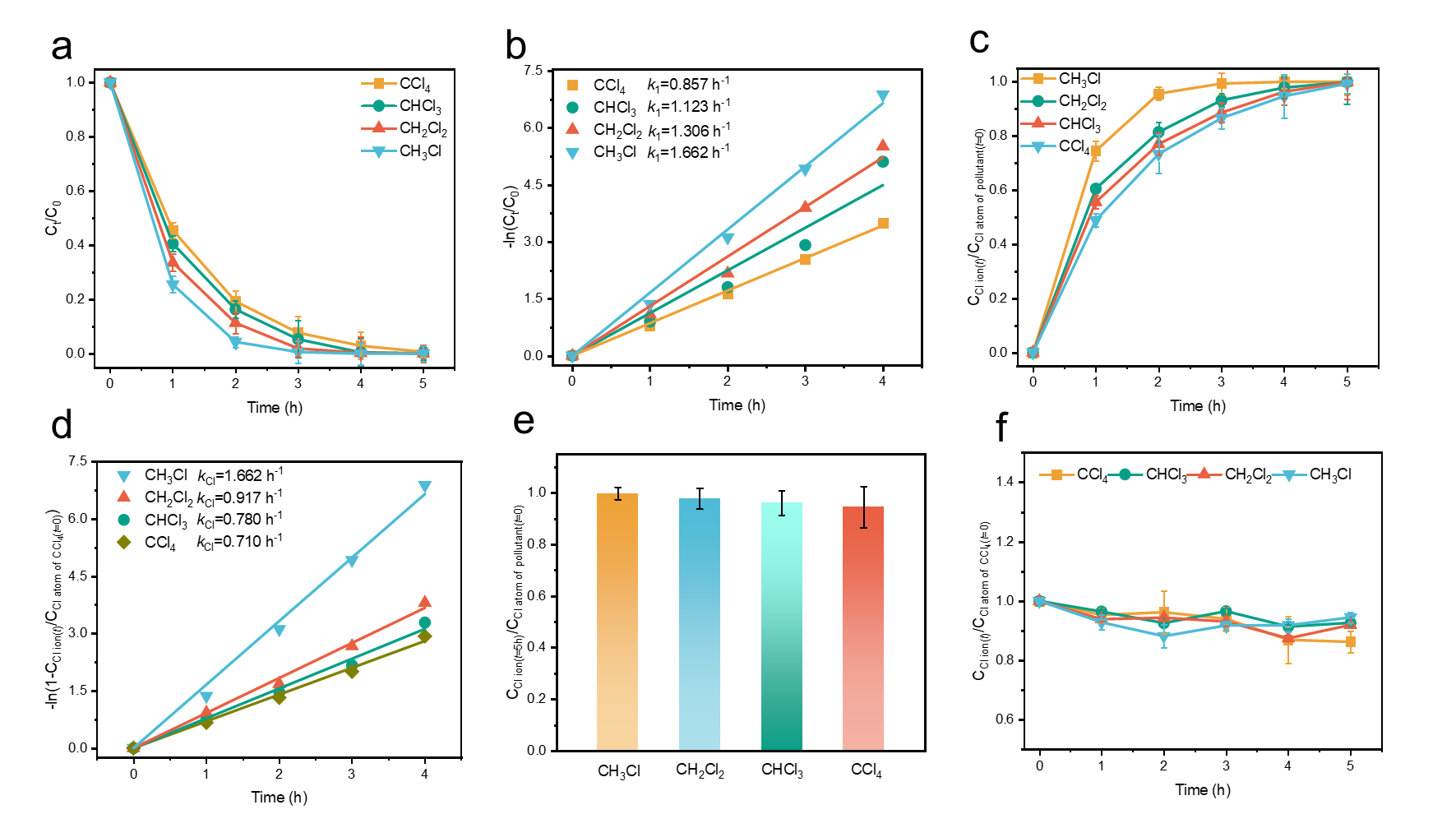
**

**Figure S41.** (a) The efficiency, (b) the corresponding pseudo-first order kinetic constants (*k*_1_), (c) concentration ratios of Cl^-^ ions in solution and Cl atoms in pollutions, (d) dechlorination rate constants for the removal of pollutions, (e) concentration ratios of Cl^-^ ions in solution and Cl atoms in pollutions during the 4-h reation, and (f) mass balance of chlorine elements of CvCu-ZVI for different pollutions removal. (Reaction conditions: [CvCu-ZVI]_0_= 0.30 g L^-1^, [CCl_4_]_0_= [CHCl_3_]_0_= [CH_2_Cl_2_]_0_= [CH_3_Cl]_0_= 5.0 mg L^-1^, T= 298 K.)


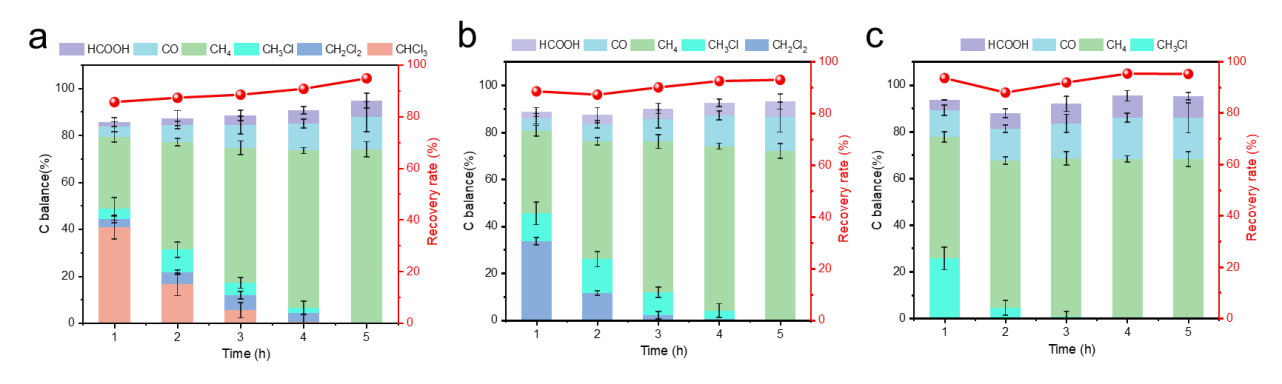


**Figure S42.** Product analysis of CvCu-ZVI for (a) CHCl_3_, (b) CH_2_Cl_2_, and (c) CH_3_Cl removal. (Reaction conditions: [CvCu-ZVI]_0_= 0.30 g L^-1^, [CHCl_3_]_0_= [CH_2_Cl_2_]_0_= [CH_3_Cl]_0_= 5.0 mg L^-1^, T= 298 K.)


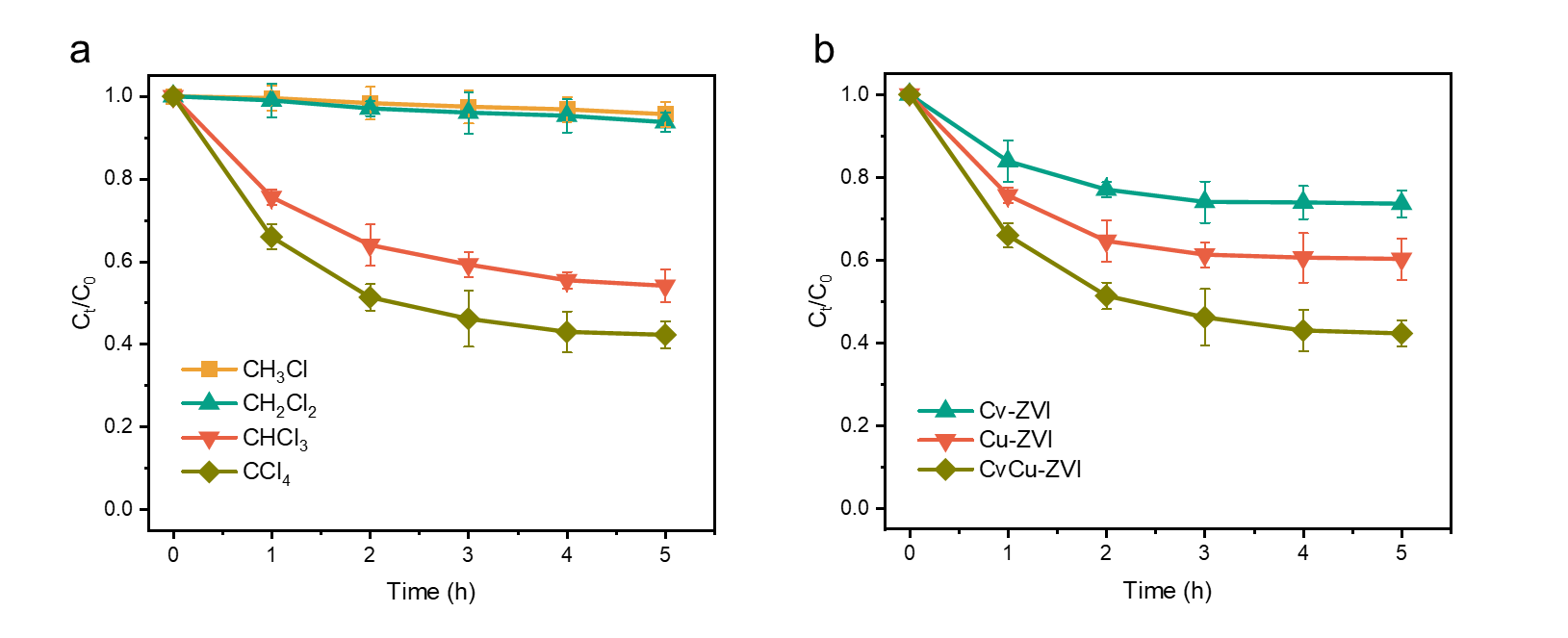


**Figure S43.** (a) The efficiency of CvCu-ZVI for CCl_4_, CHCl_3_, CH_2_Cl_2_, and CH_3_Cl removal in a double-chamber galvanic cell. (b) The efficiency of Cv-ZVI, Cu-ZVI and CvCu-ZVI for CCl_4_ removal in a double-chamber galvanic cell. (Reaction conditions: [CvCu-ZVI]_0_=[Cu-ZVI]_0_= [CvCu-ZVI]_0_=0.30 g L^-1^, [CCl_4_]_0_=[CHCl_3_]_0_= [CH_2_Cl_2_]_0_= [CH_3_Cl]_0_= 5.0 mg L^-1^, T= 298 K.)

##
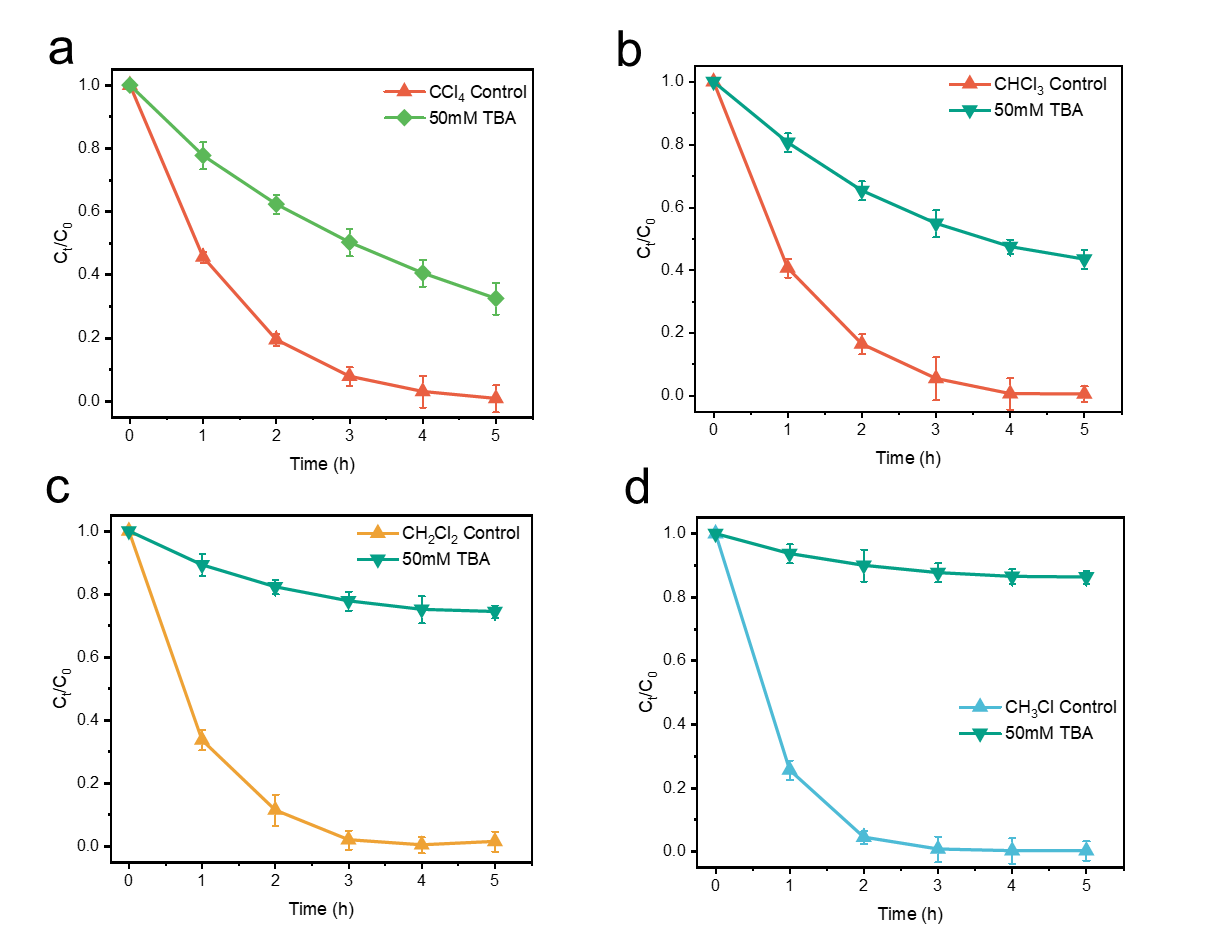


## Figure S44. The efficiency of CvCu-ZVI for (a) CCl_4_, (b) CHCl_3_, (c) CH_2_Cl_2_, and (d) CH_3_Cl removal with the addition of TBA. (Reaction conditions: [CvCu-ZVI]_0_= 0.30 g L^-1^, [CCl_4_]_0_= [CHCl_3_]_0_= [CH_2_Cl_2_]_0_= [CH_3_Cl]_0_= 5.0 mg L^-1^, T= 298 K.)

**Figure S45.** The efficiency of CvCu-ZVI for different pollutions removal. (Reaction conditions: [CvCu-ZVI]_0_= 0.30 g L^-1^, [CCl_4_]_0_= [CHCl_3_]_0_= [CH_2_Cl_2_]_0_= [CH_3_Cl]_0_= 5.0 mg L^-1^, pH= 3.0, T= 298 K.)

**
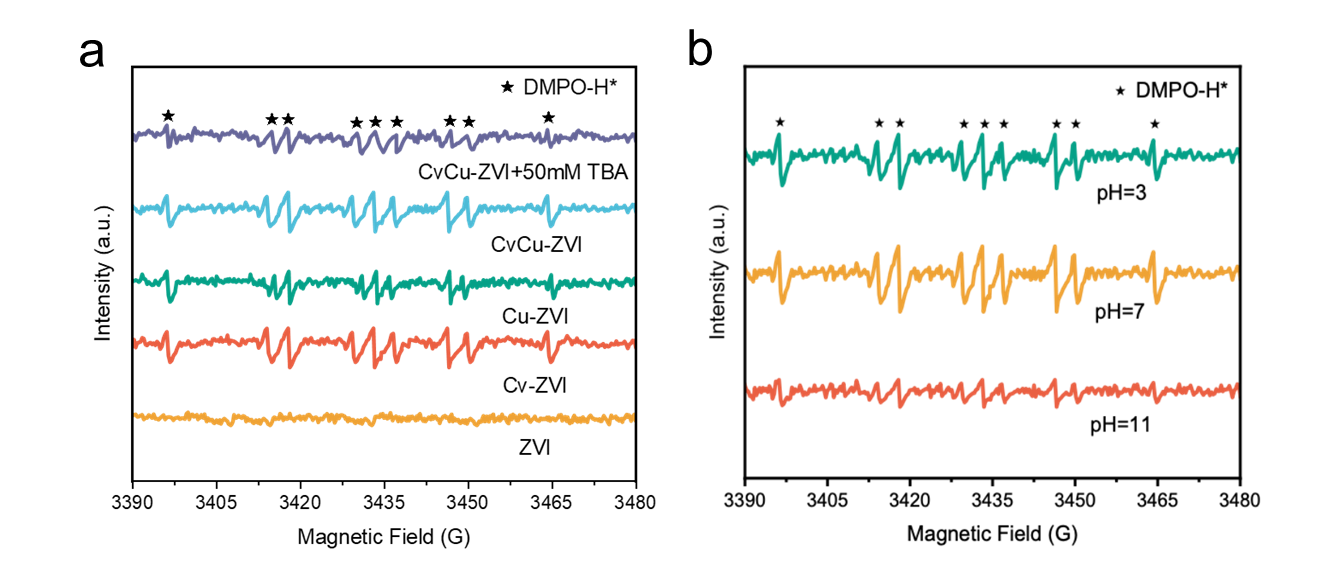
**

**Figure S46**. (a) EPR spectra of DMPO adducts recorded during materials activation and quenching effects on the removal of CCl_4_. (Reaction conditions: [CCl_4_]_0_= 5.0 mg L^-1^, [Materials]_0_= 0.30 g L^-1^, [DMPO]_0_= 1.0M, [TBA]_0_= 50.0mM, pH= 7.0, T=298K). (b) EPR spectra of DMPO adducts recorded of CvCu-ZVI for CH_2_Cl_2_ removal during different pH levels. (Reaction conditions: [CH_2_Cl_2_]_0_= 5.0 mg L^-1^, [CvCu-ZVI]_0_= 0.30 g L^-1^, [DMPO]_0_= 1.0M, pH= 3.0, 7.0, 11.0, T=298K.)


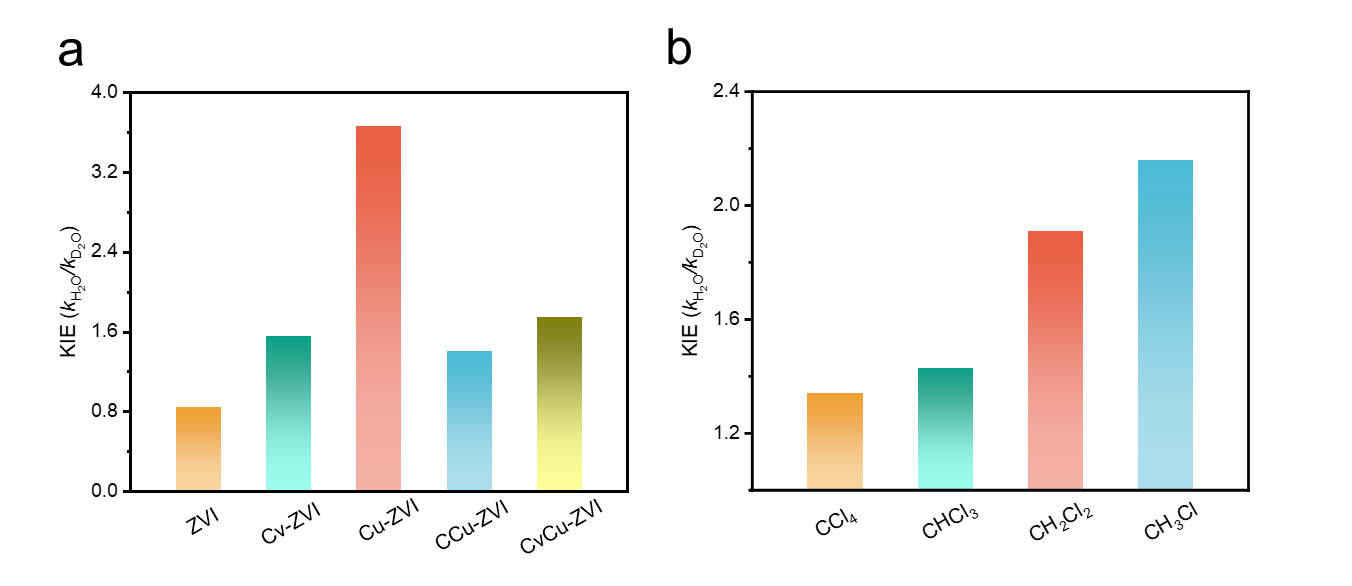


**Figure S47**. KIE plot of dechlorination rate constant ratio (KIE=$k_{H_{2}O}$*/*$k_{D_{2}O}$) on (a) different materials and (b) CvCu-ZVI during pollutants removal process. (Reaction conditions: [Materials]_0_= 0.30 g L^-1^, [CCl_4_]_0_= [CHCl_3_]_0_= [CH_2_Cl_2_]_0_= [CH_3_Cl]_0_= 5.0 mg L^-1^, T= 298 K.)

**Figure S48.** (a) Partial free energy step diagram of the DET dechlorination by Cu-ZVI, Cv-ZVI and CvCu-ZVI during CCl_4_ removal process. Free energy step diagram of (b) the direct dechlorination and (c) hydrodechlorination by Cu-ZVI and Cv-ZVI during CCl_4_ removal process. (d) Reaction mechanism for the complete dechlorination of CCl_4_ by CvCu-ZVI.

**
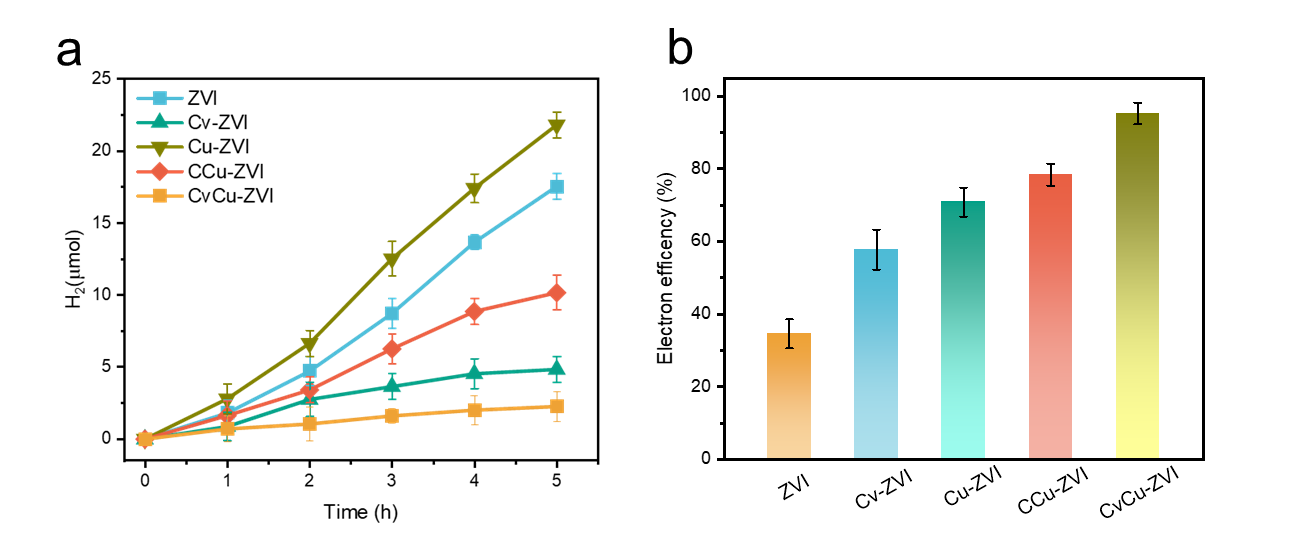
**

## Figure S49. (a) H_2_ production concentration and (b) Electron efficiency for CCl_4_ removal by different materials. (Reaction conditions: [Materials]_0_= 0.30 g L^-1^, [CCl_4_]_0_= 5.0 mg L^-1^, pH= 7.0, T=298K.)

**
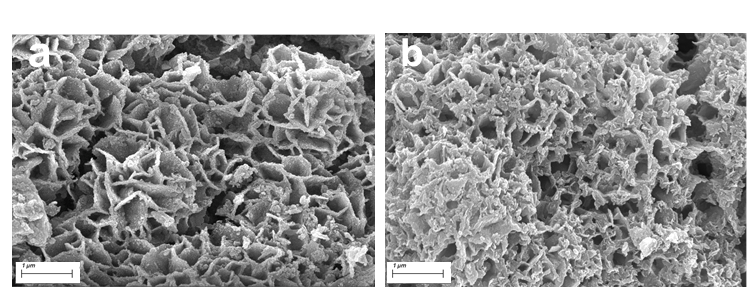
**

**Figure S50**. SEM images of CvCu-ZVI before (a) and after (b) reaction.

**Figure S51.** ^57^Fe Mössbauer spectroscopy spectra of CvCu-ZVI after reaction.

**Figure S52**. Full-scan XPS spectra of CvCu-ZVI before and after reaction.


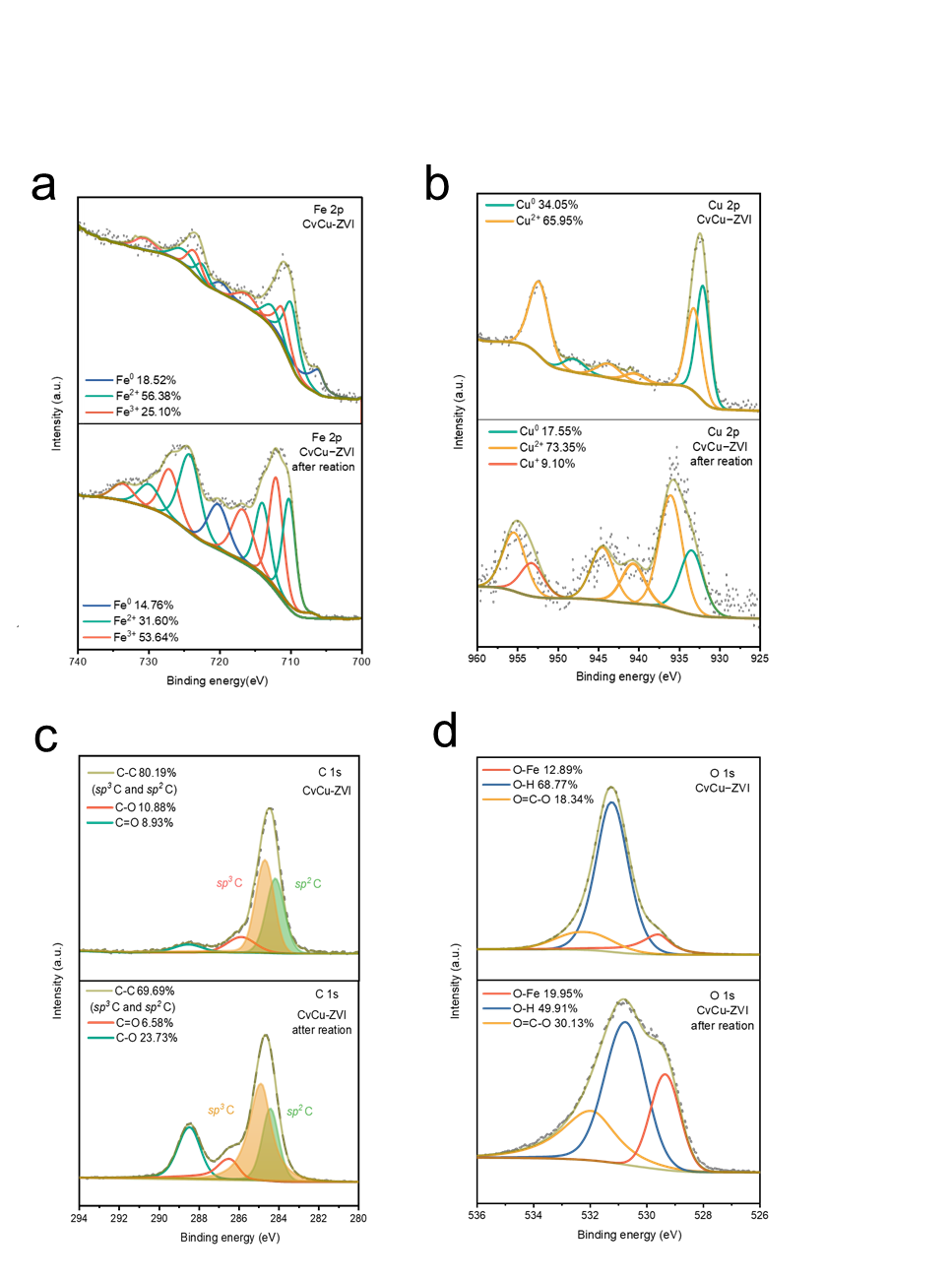


**Figure S53**. XPS analysis of (a) Fe 2p, (b) Cu 2p, (c) C 1s, and (d) O 1s spectra of CvCu-ZVI before and after reaction.


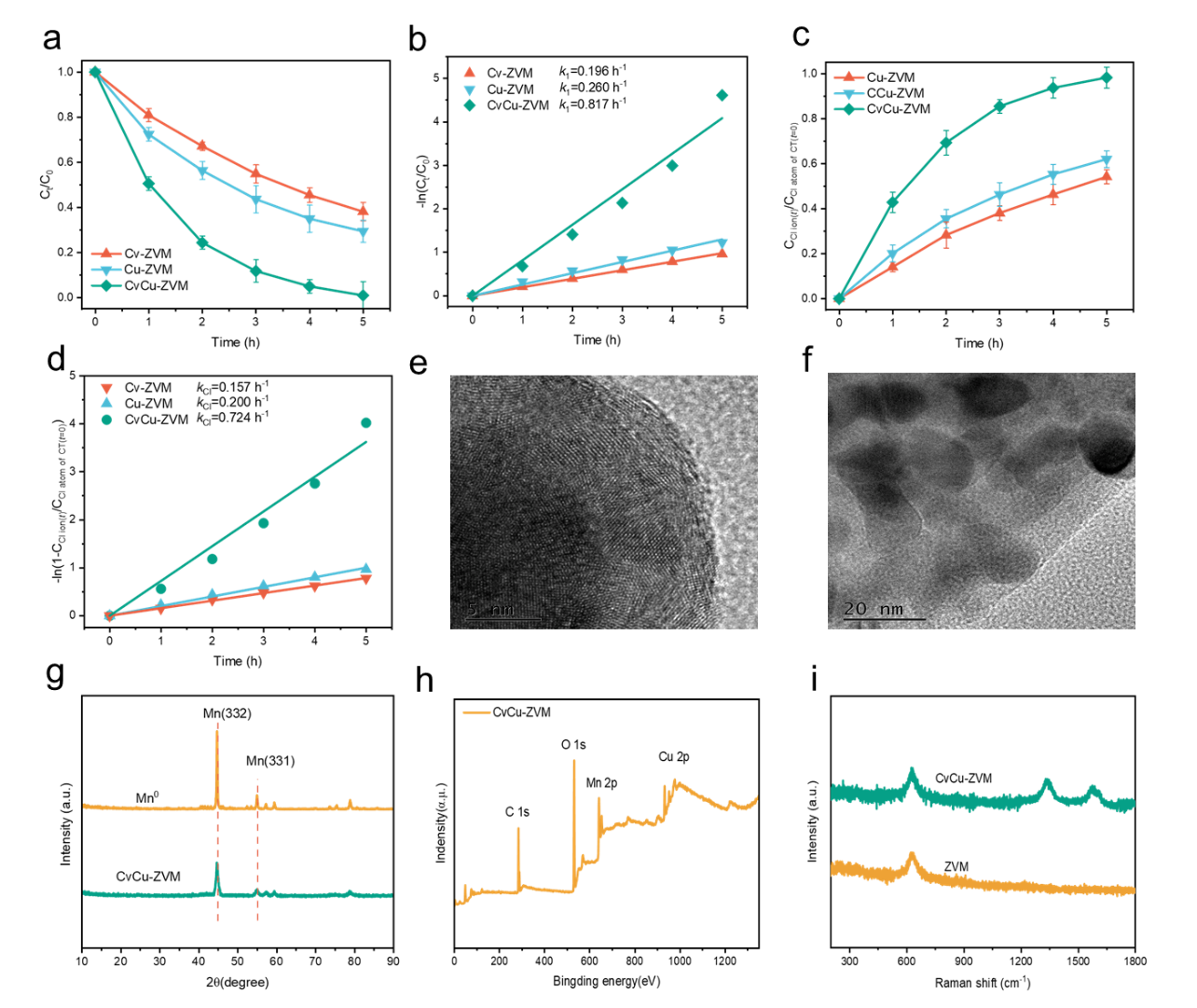


**Figure S54.** (a) The efficiency, (b) the corresponding pseudo-first order kinetic constants (*k*_1_), (c) concentration ratios of Cl^-^ ions in solution and Cl atoms in original CCl_4_, and (d) dechlorination rate constant of CvCu-ZVM for CCl_4_ removal. (Reaction conditions: [CvCu-ZVM]_0_= 0.30 g L^-1^, [CCl_4_]_0_= 5.0 mg L^-1^, T= 298 K.) (e, f) High-resolution transmission electron microscopy images images, (g) XRD pattern results, (h) XPS spectra, and (i) Raman spectra of CvCu-ZVM.

**
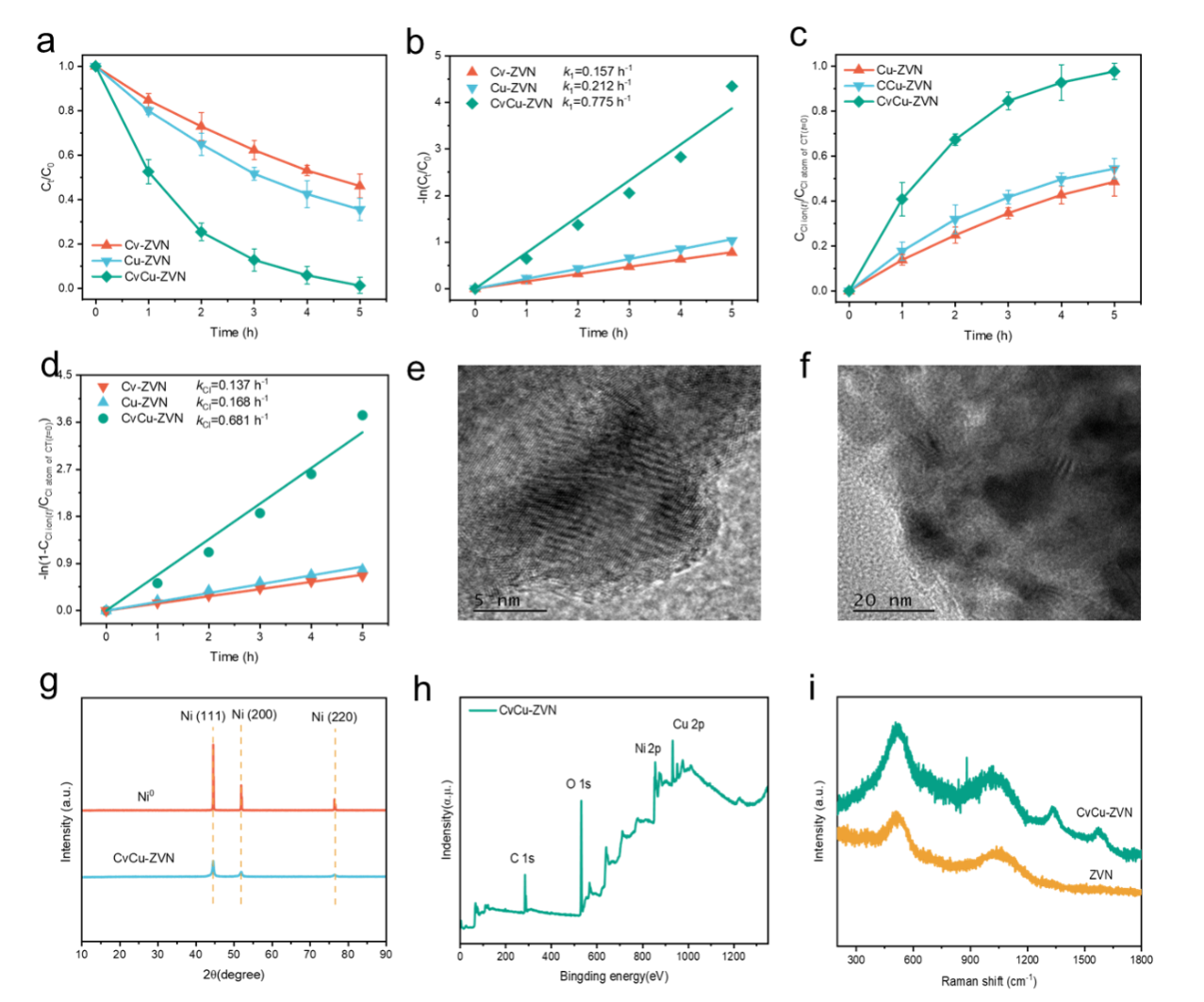
**

**Figure S55.** (a) The efficiency, (b) the corresponding pseudo-first order kinetic constants (*k*_1_), (c) concentration ratios of Cl^-^ ions in solution and Cl atoms in original CCl_4_, and (d) dechlorination rate constant of CvCu-ZVN for CCl_4_ removal. (Reaction conditions: [CvCu-ZVN]_0_= 0.30 g L^-1^, [CCl_4_]_0_= 5.0 mg L^-1^, T= 298 K.) (e, f) High-resolution transmission electron microscopy images, (g) XRD pattern results, (h) XPS spectra, and (i) Raman spectra of CvCu-ZVN.

##
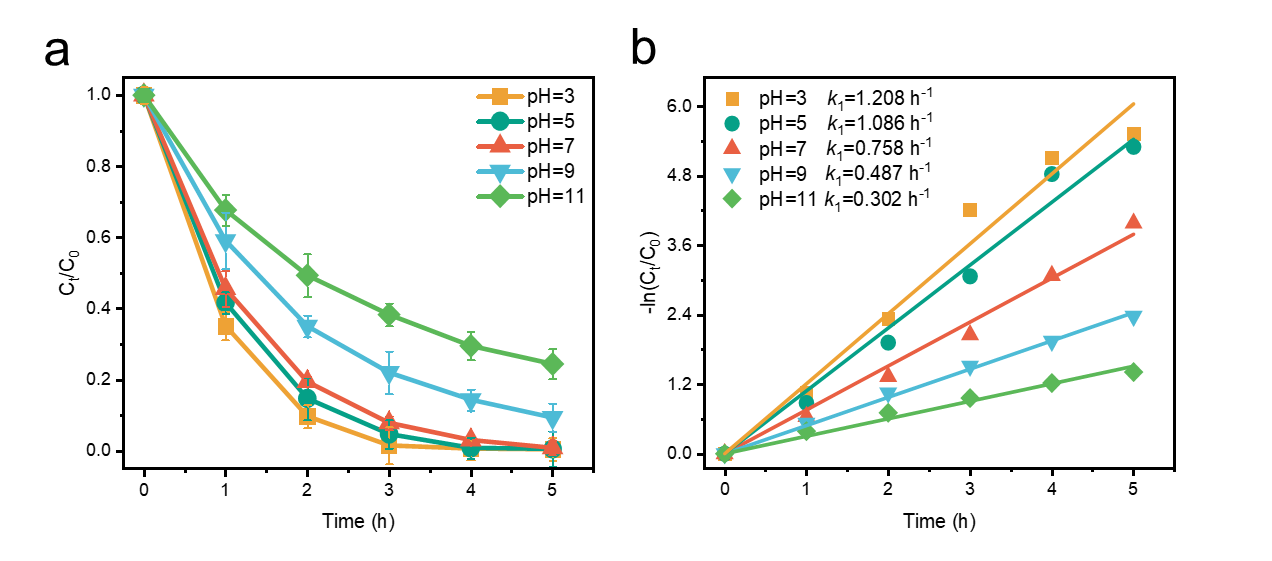


**Figure S56.** (a) The efficiency of CvCu-ZVI for CCl_4_ removal under different pH and (b) the corresponding pseudo-first rate constant (*k*_1_). (Reaction conditions: [CvCu-ZVI]_0_= 0.30 g L^-1^, [CCl_4_]_0_= 5.0 mg L^-1^, T= 298 K.)

**
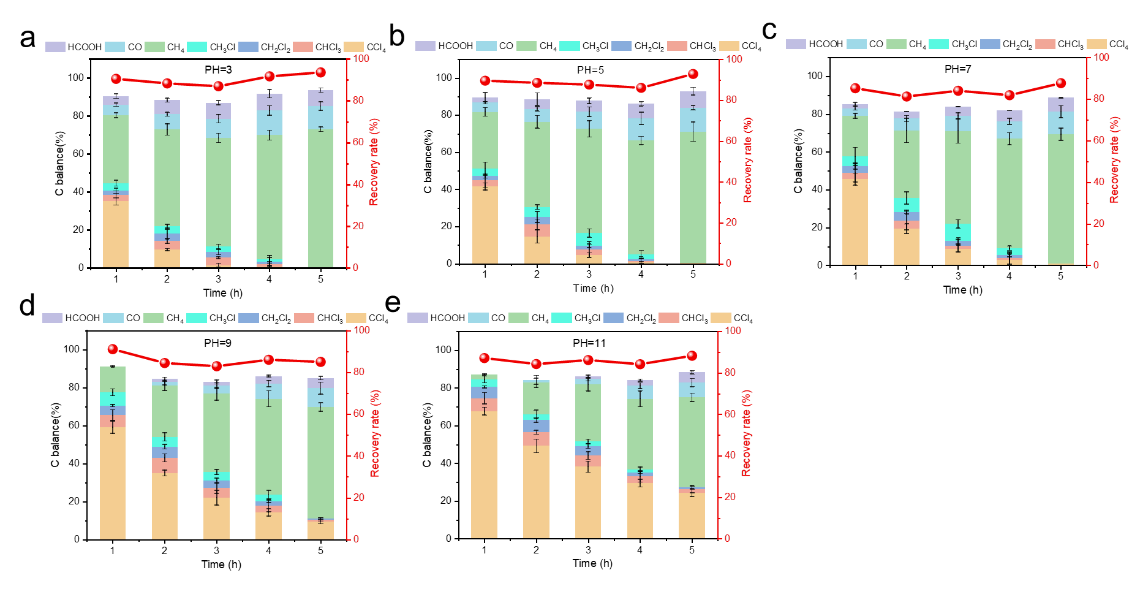
**

**Figure S57**. Product analysis of CvCu-ZVI for CCl_4_ removal under different pH. (Reaction conditions: [CvCu-ZVI]_0_= 0.30 g L^-1^, [CCl_4_]_0_= 5.0 mg L^-1^, T= 298 K.)

**
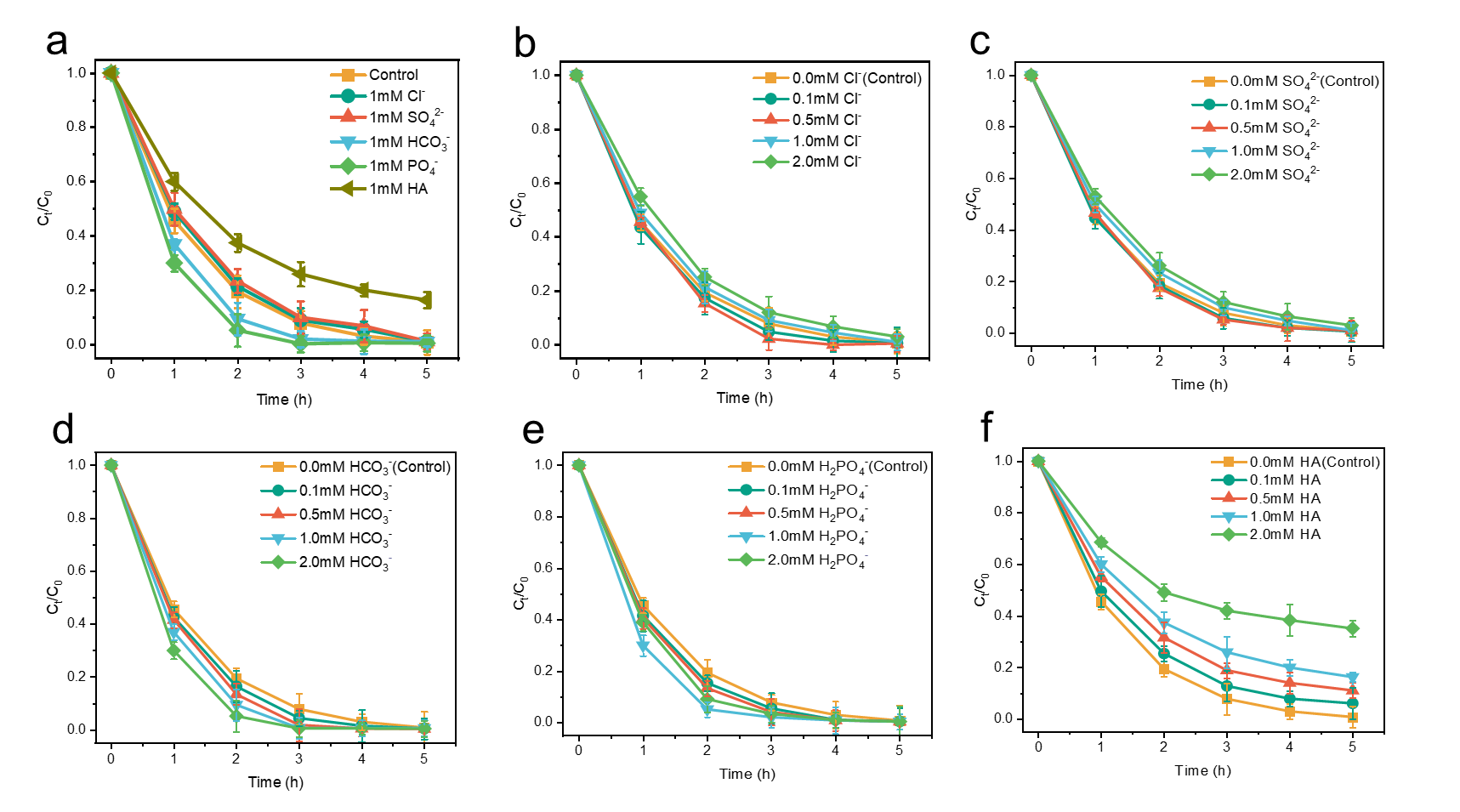
**

**Figure S58**. Effects of (a) various inorganic ions and organic substances, (b) Cl^−^ ion, (c) SO_4_^2−^ ion, (d) HCO_3_^−^ ion, (e) H_2_PO^4−^ ion, and (f) HA on CCl_4_ removal by CvCu-ZVI. (Reaction conditions: [CvCu-ZVI]_0_= 0.30 g L^-1^, [CCl_4_]_0_= 5.0 mg L^-1^, T= 298 K.)

Note: The influence of environmental factors, including inorganic ions (Cl^-^, SO_4_^2-^, HCO_3_^-^, and H_2_PO_4_^-^) and dissolved organic matter (humic acid, HA), on CCl_4_ removal by CvCu-ZVI was preliminarily evaluated. CvCu-ZVI exhibited high CCl_4_ degradation rates across various anionic conditions. Cl^-^ and SO_4_^2-^ showed minimal impact on the reductive dechlorination by CvCu-ZVI. In contrast, HCO_3_^-^ and H_2_PO_4_^-^ facilitated the dechlorination process. However, HA was found to inhibit CCl_4_ removal by CvCu-ZVI. As the HA concentration increased, the material′s corrosion intensity also escalated, leading to greater losses, reduced electron transfer capability, and an increase in the thickness of the surface iron oxide passivation layer, thereby diminishing the reactivity of CvCu-ZVI towards CCl_4_.

**
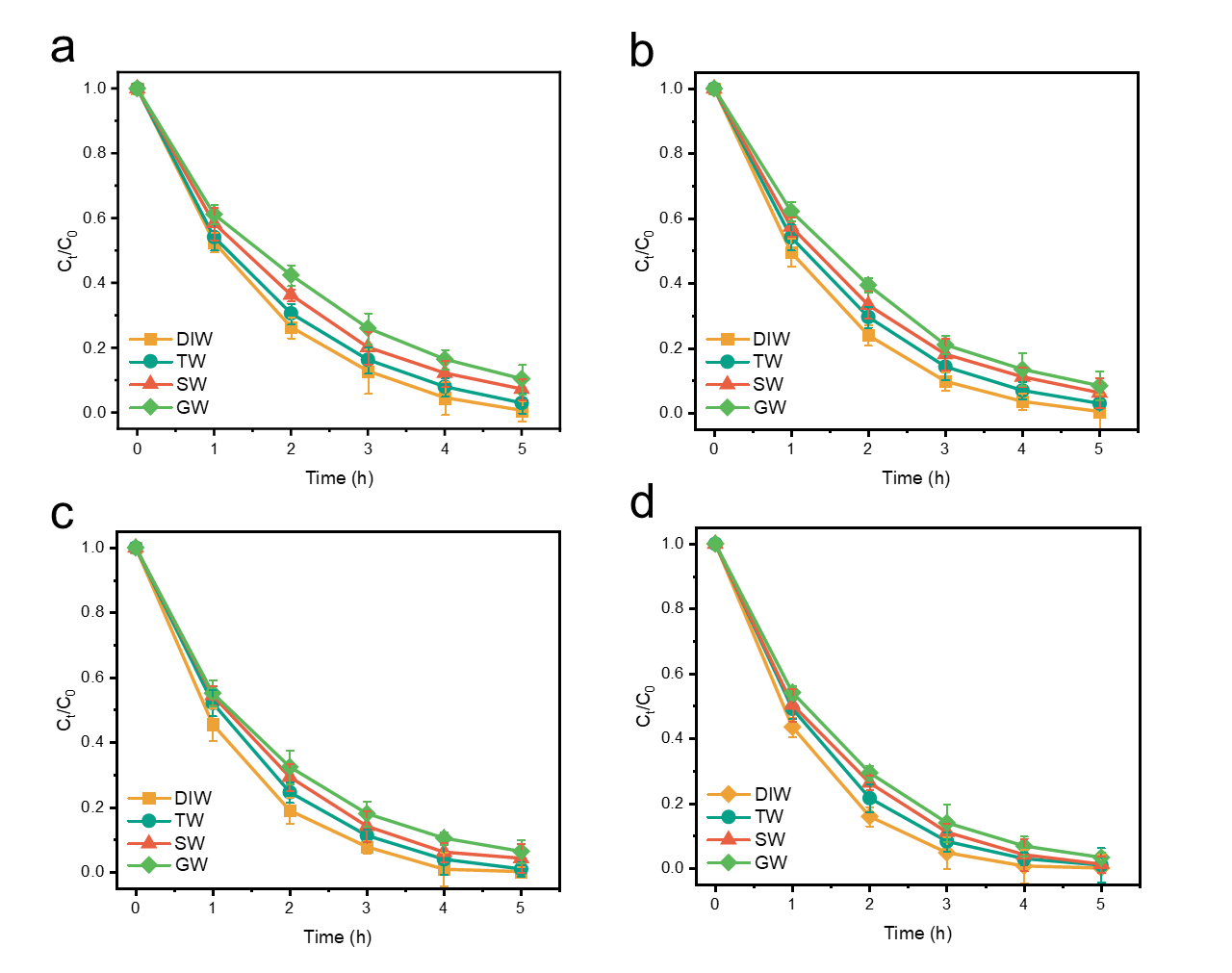
**

**Figure S59**. Effects of water substrate on (a) CCl_4_, (b) CHCl_3_, (c) CH_2_Cl_2_ and (d) CH_3_Cl removal by CvCu-ZVI. (Reaction conditions: [CvCu-ZVI]_0_= 0.30 g L^-1^, [CCl_4_]_0_=[CHCl_3_]_0_= [CH_2_Cl_2_]_0_=[ CH_3_Cl]_0_=5.0 mg L^-1^, T= 298 K.)

Note: CvCu-ZVI attained over 90% removal efficiency for CCl_4_, CHCl_3_, CH_2_Cl_2_, and CH_3_Cl in groundwater, surface water, and tap water in 5 h.


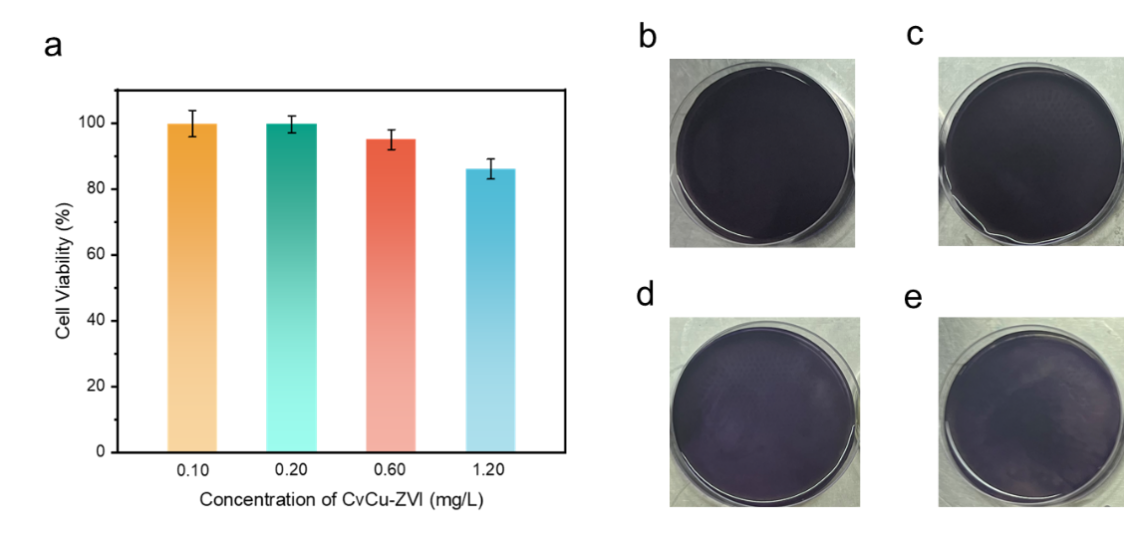


**Figure S60**. The effects of (a) 0.10 mg L^-1^, (b) 0.20 mg L^-1^, (c) 0.16 mg L^-1^, and (d) 1.20 mg L^-1^ CvCu-ZVI on the growth of *Escherichia coli* analyzed using a cytotoxicity assay.


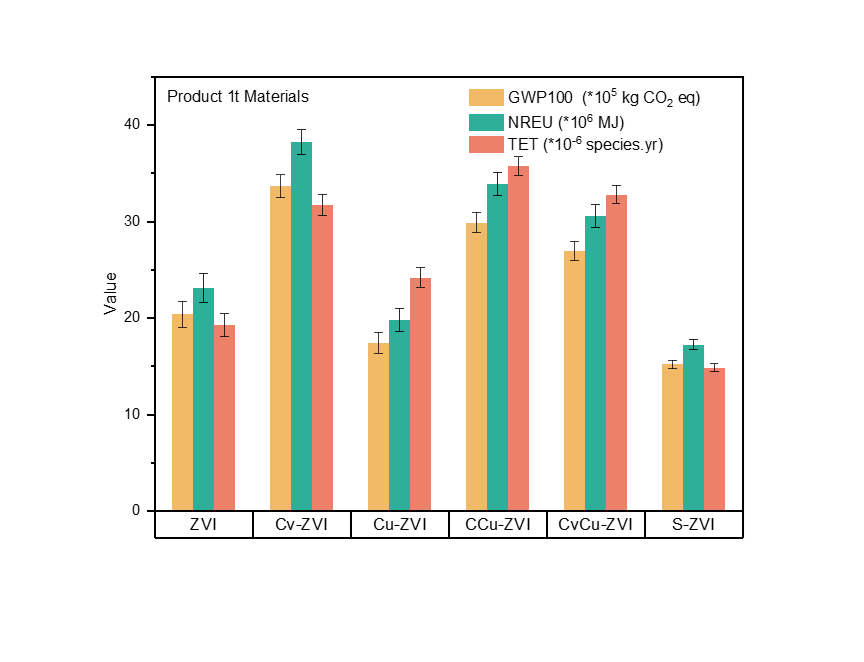


**Figure S61**. Life cycle assessment results per functional unit for the production of 1 tonne of materials, including GWP, NREU, and TET.


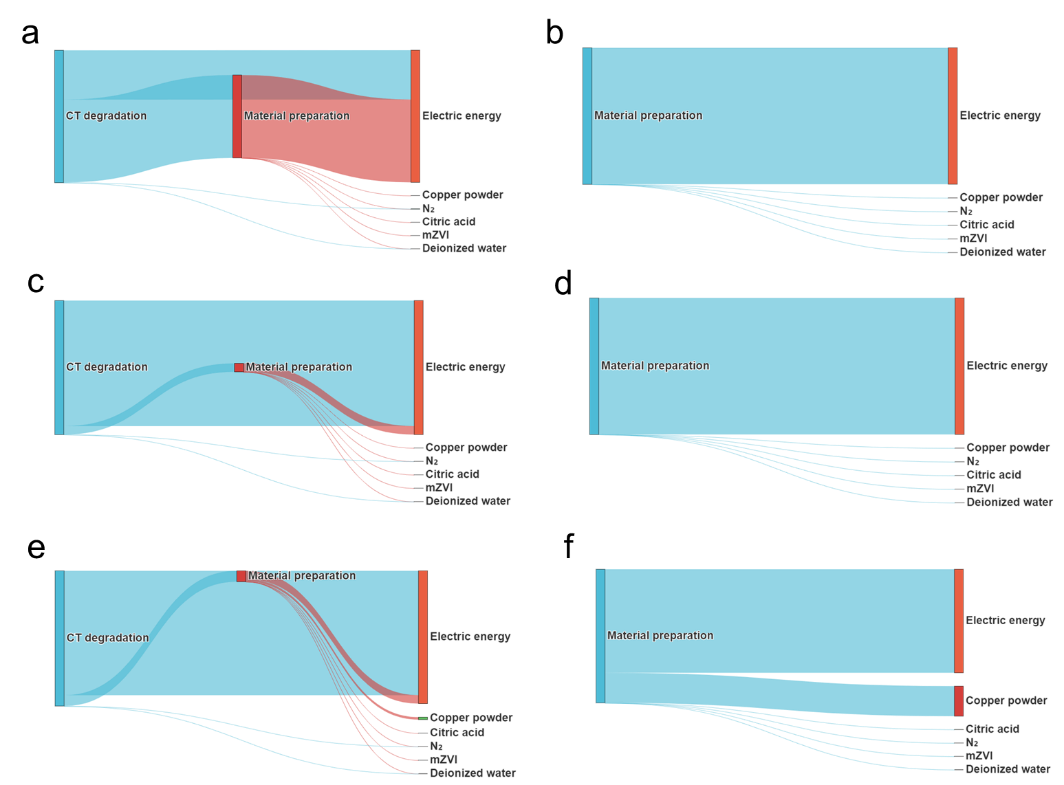


**Figure S62**. Sankey diagram of functional unit for the production of 1 ton of materials and the treatment of 1000 L of groundwater by CvCu-ZVI, including (a, b) GWP, (c, d) NREU, and (e, f) TET.


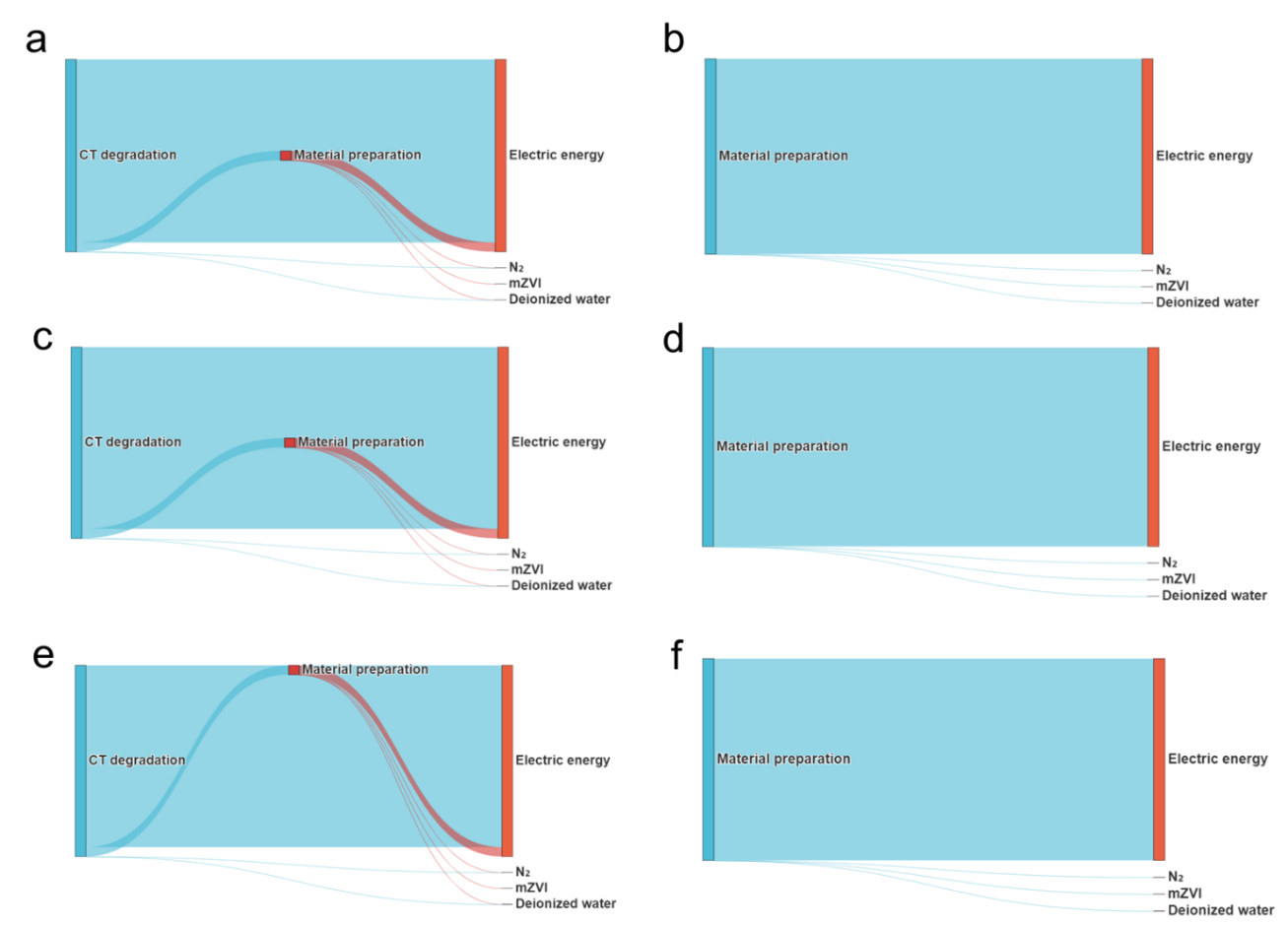


**Figure S63**. Sankey diagram of functional unit for the production of 1 ton of materials and the treatment of 1000 L of groundwater by ZVI，including (a, b) GWP, (c, d) NREU, and (e, f) TET.

Note: The electricity was the primary contributor to the GWP and NREU impacts across the life cycle stages of CCl_4_ reduction facilitated by CvCu-ZVI, while other chemicals such as copper powder made a minor contribution. This is because mechanical ball milling relies on high electricity, thus leading to high energy consumption. Given that thermal power generation (comprising 67.88% of electricity generation) predominantly relies on highly polluting fossil fuels such as coal and oil,^[16,17]^ the impact of electrical energy on GWP and NREU is consequently higher. The impact of other chemicals, such as copper powders, on TET is more specific and is primarily driven by the leaching of potentially toxic metal ions and depletion of non-metals from the material.


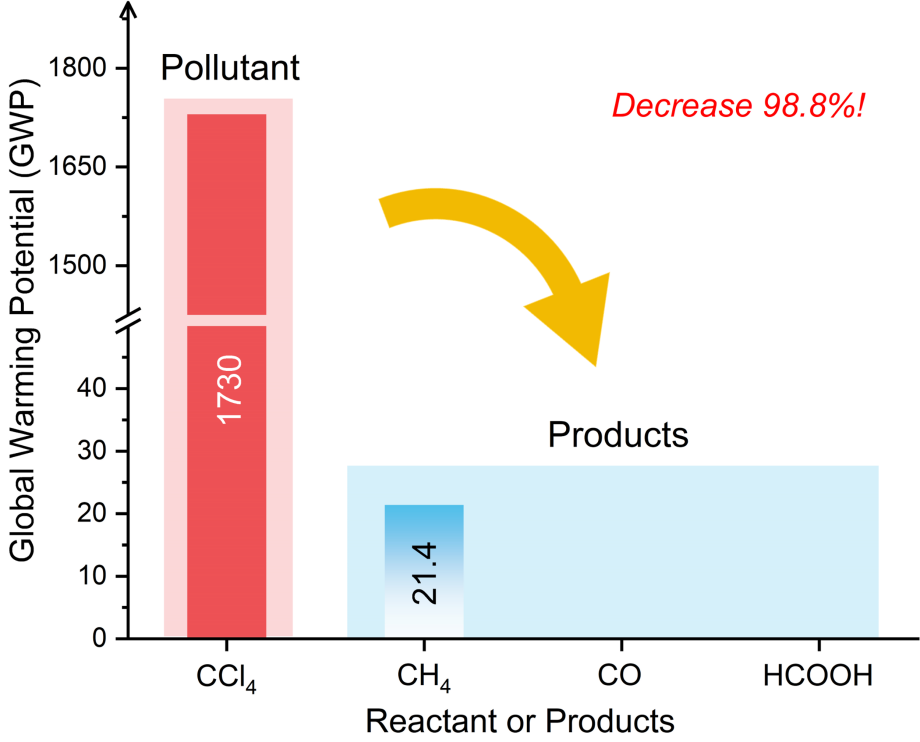


**Figure S64**. The impact of CCl_4_ conversion products on the global greenhouse effect.

Note: In order to explain the impact of CCl_4_ conversion products (e.g., methane, carbon monoxide, and formic acid) on the global greenhouse effect, the Global Warming Potential (GWP100) was used as an assessment metric. In this study, CCl_4_ was converted to methane, carbon monoxide and formic acid by 72.3%, 12.0% and 5.8%, respectively. According to the IPCC assessment report, carbon monoxide was not classified as a greenhouse gas with significant GWP due to its short atmospheric lifetime and lack of direct infrared absorption. In addition, despite the volatility of formic acid at room temperature, the low probability of trace amounts of generated formic acid leaving groundwater and entering the atmosphere precluded an effect on the greenhouse effect. According to the Montreal Protocol and Kyoto Protocol reports,^[18,19]^ the GWPs for CCl_4_ and methane were 1730 and 30, respectively, i.e., the emission of 1 kg of CCl_4_ and 1 kg of methane was equivalent to 1,730 kg and 30 kg of CO_2_ emission equivalents, respectively. It was calculated that for every 1kg of CCl_4_ removed in this study, the GWP was significantly reduced by 98.8%, which not only provided an efficient and feasible remediation solution for groundwater contaminated with highly toxic halogenated hydrocarbons, but also greatly reduced the GWP.

# Supplementary Tables

**Table S1.** Specific surface area, pore size, and XPS elementary composition (wt %) of different materials.

|  | | ZVI | Cv-ZVI | Cu-ZVI | CCu-ZVI | CvCu-ZVI |
| --- | --- | --- | --- | --- | --- | --- |
| Specific surface area（m^2^ g^-1^) | | 10.18 | 890.55 | 190.36 | 573.33 | 1090.86 |
| Pore size（nm） | | 0.84 | 40.50 | 1.37 | 7.22 | 18.13 |
| XPS  elementary composition (wt %） | Fe 2p | 28.60 | 13.19 | 12.72 | 9.27 | 7.29 |
|  | Cu 2p | 0.81 | 1.55 | 7.11 | 4.85 | 5.25 |
|  | C 1s | 35.41 | 65.58 | 30.23 | 51.50 | 60.57 |
|  | O 1s | 35.18 | 19.68 | 49.94 | 34.38 | 26.89 |

**Table S2.** EDS elementary composition (wt %) of different materials.

|  | | CvCu-ZVI |
| --- | --- | --- |
| EDS elementary composition (wt %) | Fe | 65.924 |
|  | Cu | 8.673 |
|  | C | 15.212 |
|  | O | 10.191 |

**Table S3.** ICP-OES elementary composition (wt %) of different materials.

|  | | Cu-ZVI | CCu-ZVI | CvCu-ZVI |
| --- | --- | --- | --- | --- |
| ICP-OES elementary composition (wt %) | Fe | 85.728 | 56.167 | 69.588 |
|  | Cu | 9.153 | 8.363 | 9.942 |

Note: The Cu content of CvCu-ZVI was higher than that of Cu-ZVI (9.94 wt% vs. 8.36 wt%). This phenomenon likely occurred due to the disappearance of the dense FeO*_x_* layer on the surface, which induced the spontaneous diffusion of Fe from the core to the exterior, thereby prompting Cu with a similar atomic radius (1.17 vs. 1.16) to migrate inward (a Kirkendall-like effect).

**Table S4.** The structural parameters extracted from the Fe *K*-edge EXAFS fitting.

| materials | Shell | | CN**^[a]^** | | R**^[b]^**(Å) | σ^2^**^[c]^** (Å^2^) | ∆E_0_**^[d]^**(eV) | R factor**^[e]^** |
| --- | --- | --- | --- | --- | --- | --- | --- | --- |
| Fe foil | | Fe-Fe^1^ | | 8 | 2.45±0.023 | 0.0063 | 1.455±1.024 | 0.0032 |
|  |  | Fe-Fe^2^ | | 6 | 2.82±0.016 | 0.0038 | 1.455±1.236 |  |
| CCu-ZVI | | Fe-Fe | | 4.41±0.16 | 2.48±0.014 | 0.0126 | 8.247±2.021 | 0.0027 |
| CvCu-ZVI | | Fe-Fe | | 4.24±0.20 | 2.46±0.025 | 0.0135 | 9.379±2.724 | 0.0035 |

1. CN was coordination numbers;
2. R was bond distance;
3. σ^2^ was Debye-Waller factors;
4. ΔE_0_ was the inner potential correction;
5. R factor reflected goodness of fitting.

**Table S5.** Hyperfine parameters obtained for CvCu-ZVI with ^57^Fe Mössbauer spectra collected at 77 K.

|  | IS**^[a]^** (mm s^-1^) | QS**^[b]^** (mm s^-1^) | H**^[c]^** (T) | *Γ***^[d]^**(mm s^-1^) | Area (%) |
| --- | --- | --- | --- | --- | --- |
| a-Fe | -0.11 | 0.06 | 34.71 | 0.73 | 85 |
| Amorphous iron | -0.13 | 1.76 | — | 0.19 | 4 |
| Fe(Ⅱ)-Sextet | 0.53 | -0.01 | 45.71 | 0.61 | 4.80 |
| Fe(Ⅲ)-Sextet | 0.36 | -0.10 | 20 | 0.79 | 6.20 |

1. IS is isomer shifts;
2. QS is quadrupole splitting;
3. H is hyperfine fields;
4. *Γ* is Line width.

**Table S6.** Highest unoccupied molecular orbital (HOMO) and lowest unoccupied molecular orbital (LUMO) results for intermediates and products during the degradation of CCl_4_, PCE, TCP, and TOB by CvCu-ZVI.

|  | | HOMO（eV） | LOMO（eV） |
| --- | --- | --- | --- |
| Chloromethane | CCl_4_ | -7.583 | -2.636 |
|  | CHCl_3_ | -7.340 | -1.960 |
|  | CH_2_Cl_2_ | -7.197 | -1.666 |
|  | CH_3_Cl | -6.943 | -0.150 |
| Chloroethylene | PCE | -5.933 | -2.937 |
|  | TCE | -5.938 | -2.791 |
|  | trans-DCE | -5.913 | -2.631 |
|  | cis-DCE | -5.916 | -2.549 |
|  | EtCl | -5.978 | -2.331 |
| Chlorophenol | TCP | -5.970 | -2.837 |
|  | DCP | -5.832 | -2.640 |
|  | CP | -5.666 | -2.424 |
| chlorobenzene | TOB | -6.083 | -2.779 |
|  | 1,4-DCB | -5.953 | -2.576 |
|  | 1,2-DCB | -6.039 | -2.515 |
|  | 1,3-DCB | -6.096 | -2.540 |
|  | MCB | -5.959 | -2.279 |

**Table S7.** Summary of published intrinsic activity rate constants (*k*_IA_) and dechlorination degree of Fe-based materials for CCl_4_ removal.

| Materials | CCl_4_ removal | Dechlorination | Preparation method | CCl_4_  concentration（mg L^-1^） | *k*_Cl_*(*h^-1^*)* | Dechlorination products | Materials dosage（g L^-1^） | *k*_IA_**^[a]^**（10^-3^ h^-1^） | Ref. |
| --- | --- | --- | --- | --- | --- | --- | --- | --- | --- |
| CvCu-ZVI | 100%，5h | 100%，5h | ball-milling | 5 | 0.822 | Mainly CH_4_, CO, and C_3_HOOH | 0.3 | 13.700 | This work |
| CCu-ZVI | 72%，6h | 57%，6h | ball-milling | 5 | 0.222 | Mainly CHCl_3_, CH_2_Cl_2_, and CH_4_ | 0.3 | 3.700 | This work |
| Cu-ZVI | 69%，6h | 50%，6h | ball-milling | 5 | 0.174 | Mainly CHCl_3_, CH_2_Cl_2_, and CH_4_ | 0.3 | 2.900 | This work |
| Cv-ZVI | 61%，6h | 31%，6h | ball-milling | 5 | 0.108 | Mainly CHCl_3_, with small amounts of CH_2_Cl_2_ and CH_4_ | 0.3 | 1.800 | This work |
| ZVI | 45%，6h | 19%，6h | ball-milling | 5 | 0.055 | Mainly CHCl_3_, with small amounts of CH_2_Cl_2_ | 0.3 | 0.917 | This work |
| nZVI | 95%，10h | 7.25%，10h | Liquid phase reduction | 7.7 | 0.008 | Mainly CHCl_3_, with small amounts of CH_2_Cl_2_ | 0.3 | 0.116 | [20] |
| EDTA-ZVI | 98.9%, 8h | 89.1%，8h | commercial | 20 | 0.277 | Mainly CHCl_3_, with small amounts of CH_2_Cl_2_ | 5 | 1.108 | [21] |
| S-ZVI | 91%，2h | 19%，2h | Liquid phase reduction | 3 | 0.105 | CHCl_3_ | 0.5 | 0.632 | [22] |
| Fe^0^/Fe_3_O_4_ | 100%，1h | 8.38%，5h | Liquid phase reduction | 2 | 0.018 | Mainly CHCl_3_, with small amounts of CH_2_Cl_2_ | 0.5 | 0.070 | [23] |
| r-NZVI | 100%，9h | 35.25%，9h | Liquid phase reduction | 15.4 | 0.048 | Mainly CHCl_3_, with small amounts of CH_2_Cl_2_ and CH_4_ | 0.5 | 1.487 | [24] |
| Pd/FePs@G | 93.5%，50min | 24.26%，50min | Liquid phase reduction | 3 | 0.305 | Mainly CHCl_3_, with small amounts of CH_2_Cl_2_ | 0.5 | 1.829 | [25] |
| u-n  ZVI@SN-rGO | 95.07%，3h | 16.27%，3h | Liquid phase reduction | 3 | 0.059 | No chloromethane | 0.4 | 0.444 | [26] |
| SC-NZVAl | 100%，7h | 4.5%，18h | commercial | 15.4 | 0.003 | Mainly CHCl_3_, with small amounts of CH_2_Cl_2_ | 4 | 0.010 | [27] |
| S-nZVI | 100%，0.2h | 19.7% ，0.54h | Liquid phase reduction | 7.7 | 0.399 | Mainly CHCl_3_, with small amounts of CH_2_Cl_2_ | 1.23 | 2.500 | [28] |
| Pd-nZVI | 100%，0.51h | 25.0 %，0.91h | Liquid phase reduction | 7.7 | 0.316 | Mainly CHCl_3_, with small amounts of CH_2_Cl_2_ | 1.23 | 1.979 | [28] |
| Ag-Fe | 98%，40min | 12.75%，1h | impregnate | 20 | 0.136 | Mainly CHCl_3_, with small amounts of CH_2_Cl_2_ | 20 | 0.136 | [29] |
| Pd-Fe | 100%，5min | 13.73%，110min | impregnate | 4 | 0.081 | Mainly CHCl_3_, with small amounts of CH_2_Cl_2_ | 4.83 | 0.067 | [30] |
| FHC | 96%，90min | 53.04%，1.5h | impregnate | 3.08 | 0.504 | Mainly CHCl_3_, with small amounts of CH_2_Cl_2_, CH_4_ and CO | 1.8 | 0.862 | [31] |

1. *k*_IA_=*w***k*_Cl_ *m^-1^*, where *w* represents the concentration of CCl_4_ , *k*_Cl_ is the dechlorination rate constant, and *m* represents the materials dosage.

**Table S8.** Summary of published intrinsic activity rate constants (*k*_IA_) and dechlorination degree of Fe-based materials for TCE removal.

| Materials | TCE removal | Dechlorination | Preparation method | TCE concentration（mg L^-1^） | *k*_Cl_*(*h^-1^*)* | Materials dosage（g L^-1^） | *k*_IA_**^[a]^**（10^-3^ h^-1^） | Ref. |
| --- | --- | --- | --- | --- | --- | --- | --- | --- |
| CvCu-ZVI | 100%，4h | 100%，5h | ball-milling | 5 | 0.844 | 0.3 | 14.067 | This work |
| nZVI | 100%, 216h | 75.7%，216h | ball-milling | 20 | 0.007 | 1 | 0.131 | [32] |
| ε-Fe_x_N | 100%，216h | 45.3%，216h | ball-milling | 20 | 0.004 | 1 | 0.079 | [32] |
| Ni-nZVI | 100%，5h | 21.62%，5h | Liquid phase reduction | 0.4 | 0.049 | 1 | 0.019 | [33] |
| S-mZVI^1^ | 93.8%，24h | 87.9%，24h | ball-milling | 10 | 0.088 | 10 | 0.088 | [34] |
| mZVI^1^ | 44%，192h | 42.6%，192h | ball-milling | 10 | 0.002 | 10 | 0.002 | [34] |
| S-mZVI^2^ | 86.3%，10h | 69.1%，10h | Liquid phase reduction | 10 | 0.117 | 10 | 0.117 | [35] |
| Sand-Fe^0^ | 56.9%，360h | 42%，360h | Biological reduction | 6 | 0.002 | 50 | 0.000 | [36] |
| Sand-Fe^0^-SRB | 92.6%，360h | 84.5%，360h | Biological reduction | 6 | 0.005 | 50 | 0.001 | [36] |
| SNZVI^1^ | 51%，192h | 51%，192h | Liquid phase reduction | 11.83 | 0.004 | 1 | 0.044 | [4] |
| Ni-Fe^01^ | 15.9%，200h | 15.9%，200h | Liquid phase reduction | 9.2 | 0.001 | 1 | 0.008 | [37] |
| Ni-Fe^02^ | 100%，26.7h | 99.99%，26.7h | Liquid phase reduction | 9.2 | 0.345 | 1 | 3.174 | [37] |
| Ni-S-Fe^01^ | 94.7%，200h | 94.7%，200h | Liquid phase reduction | 9.2 | 0.015 | 1 | 0.135 | [37] |
| Ni-S-Fe^02^ | 99.1%，54h | 99.1%，54h | Liquid phase reduction | 9.2 | 0.087 | 1 | 0.803 | [37] |
| SNZVI^2^ | 62.4%，8h | 62.4%，8h | Liquid phase reduction | 4 | 0.122 | 2 | 0.245 | [1] |
| SNZVI^3^ | 98.5%，8h | 98.5%，8h | Liquid phase reduction | 4 | 0.525 | 2 | 1.050 | [1] |
| NZVI^1^ | 72%，241h | 72%，241h | Liquid phase reduction | 9.2 | 0.005 | 1 | 0.049 | [38] |
| SNZVI^4^ | 99.98%，119h | 99.98%，119h | Liquid phase reduction | 9.2 | 0.072 | 1 | 0.658 | [38] |
| mZVI^2^ | 92.7%，167h | 89.9%，167h | ball-milling | 13.14 | 0.014 | 10 | 0.018 | [39] |
| S-N(C)-mZVI | 100%，6h | 92.3%，6h | ball-milling | 13.14 | 0.427 | 10 | 0.562 | [39] |
| C-mZVI | 94.9%，480h | 93.9%，480h | ball-milling | 13.14 | 0.006 | 10 | 0.008 | [40] |
| N-C-mZVI | 94.1%，168h | 92.2%，168h | ball-milling | 13.14 | 0.015 | 10 | 0.020 | [40] |
| NZVI^2^ | 32.8%，120h | 32.8%，120h | Liquid phase reduction | 8.4 | 0.003 | 1 | 0.028 | [41] |
| SNZVI^5^ | 100%，120h | 96.3%，72.6h | Liquid phase reduction | 8.4 | 0.045 | 1 | 0.381 | [41] |
| SNZVI^6^ | 93.3%，70h | 93.3%，70h | Liquid phase reduction | 9.2 | 0.039 | 1 | 0.355 | [42] |

1. *k*_IA_=*w***k*_Cl_ *m^-1^*, where *w* represents the concentration of TCE, *k*_Cl_ is the dechlorination rate constant, and *m* represents the materials dosage.

**Table S9.** The C-Cl bond (*I*_C-Cl_) in the CCl_4_ molecules adsorbed on different materials.

|  | Cv-ZVI | Cu-ZVI | CvCu-ZVI |
| --- | --- | --- | --- |
| *I*_C-Cl_ | 2.041 | 2.142 | 2.224 |

**Table S10.** Hyperfine parameters obtained for CvCu-ZVI after reation with ^57^Fe Mössbauer spectra collected at 77 K.

|  | IS**^[a]^** (mm s^-1^) | QS**^[b]^** (mm s^-1^) | H**^[c]^** (T) | *Γ***^[d]^**(mm s^-1^) | Area (%) |
| --- | --- | --- | --- | --- | --- |
| a-Fe | -0.12 | 0.01 | 33.43 | 0.46 | 57.10 |
| Amorphous iron | 0.29 | 0.85 | — | 0.37 | 11.30 |
| Fe(Ⅱ)-Sextet | 0.40 | 0.33 | 40.89 | 0.19 | 3.20 |
| Fe(Ⅲ)-Sextet | 0.05 | 0 | 35.93 | 0.56 | 28.40 |

1. IS is isomer shifts;
2. QS is quadrupole splitting;
3. H is hyperfine fields;
4. *Γ* is Line width.

**Table S11.** Input chemical amount and costs of 1 tonne CvCu-ZVI production.

| Chemcial | Price ($ t^-1^) | Amount (t) | Total cost ($) |
| --- | --- | --- | --- |
| C_6_H_8_O_7_ | 497.47 | 0.09 | 42.34 |
| mZVI | 5700 | 1.04 | 5915.68 |
| Cu | 35000 | 0.13 | 4447.81 |
| Ar | 300 | 1.99 | 596.74 |
| Water | 0.65 | 20.80 | 13.52 |

**Table S12.** Capacity, electricity power and price of equipment used in the techno-economic analysis.

| Equipment | Capacity | Electrical power (kW) | Price ($) |
| --- | --- | --- | --- |
| Water purification apparatus | 4 m^3^ h^-1^ | 13.3 | 36892.13 |
| Planetary ball mill | 0.002 m^3^ | 0.75 | 3,868.54 |
| Glove box | 0.113 m^3^ | 2 | 7,571.28 |

**Table S13.** Techno-economic analysis results for other materials.

| ZVI | Feed stock ($ ton^-1^) | | | | | | Manufacturing expenses ($ ton^-1^) | | | | Ball-mi-lling  ($ ton^-1^) | Total cost  ($ ton^-1^) | Ref. |
| --- | --- | --- | --- | --- | --- | --- | --- | --- | --- | --- | --- | --- | --- |
|  | FeCl_3_ | NaBH_4_ | Water | mZVI | Cu  powder | Citri-  cacid | Water purific-ation | Agitat-or  tank | Ssepar-ator | Vacu-um  oven |  |  |  |
| Commercial nZVI^1^ | - | - | - | - | - | - | - | - | - | - | - | 80000 | [26] |
| Commercial nZVI^2^ | - | - | - | - | - | - | - | - | - | - | - | 1400000 | [26] |
| Commercial nZVI^3^ | - | - | - | - | - | - | - | - | - | - | - | 1500000 | [26] |
| Laboratorial nZVI | 33460 | 1857590 | - | - | - | - | - | - | - | - | - | 1891050 | [26] |

**Table S14.** Life cycle assessment results of preparing different materials, functional unit = 1 tonne materials.

| Material | NREU  (*10^6^ MJ) | Error bar (*10^6^ +) | GWP100  (*10^5^ kg CO_2_ eq) | Error bar (*10^6^ +) | TET  (*10^-6^species.yr) | Error bar (*10^-6^+) |
| --- | --- | --- | --- | --- | --- | --- |
| CvCu-ZVI | 30.56 | 1.155 | 26.95 | 1.026 | 32.75 | 9.436 |
| CCu-ZVI | 33.89 | 1.191 | 29.88 | 1.054 | 35.74 | 9.443 |
| Cu-ZVI | 19.78 | 1.234 | 17.44 | 1.091 | 24.17 | 1.016 |
| Cv-ZVI | 38.18 | 1.293 | 33.66 | 1.142 | 31.72 | 1.122 |
| ZVI | 23.09 | 1.534 | 20.36 | 1.358 | 19.22 | 1.182 |
| S-nZVI | 38.52 | 0.725 | 33.94 | 0.645 | 32.24 | 5.921 |

**Table S15.** Life cycle assessment results of CCl_4_ removal by different materials, functional unit = 1000 L groundwater.

| Material | NREU  (*10^6^ MJ) | Error bar (*10^6^+) | GWP100  (*10^5^ kg CO_2_ eq) | Error bar (*10^6^ +) | TET  (*10^-6^ species.yr) | Error bar (*10^-6^+) |
| --- | --- | --- | --- | --- | --- | --- |
| CvCu-ZVI | 14.96 | 1.260 | 13.19 | 1.110 | 12.65 | 1.043 |
| CCu-ZVI | 18.61 | 1.464 | 16.40 | 1.291 | 15.76 | 1.184 |
| Cu-ZVI | 19.54 | 1.668 | 17.22 | 1.472 | 16.59 | 1.361 |
| Cv-ZVI | 30.34 | 2.394 | 26.74 | 2.111 | 25.30 | 2.071 |
| ZVI | 41.19 | 3.431 | 36.32 | 3.025 | 33.85 | 2.826 |
| S-nZVI | 21.40 | 2.479 | 18.88 | 2.186 | 17.73 | 2.001 |

# References

1. Xu, J., Cao, Z., Zhou, H., et al., “Sulfur dose and sulfidation time affect reactivity and selectivity of post-sulfidized nanoscale zerovalent iron,” *Environ. Sci. Technol.* **2019**, 53, 13344-13352. <https://doi.org/10.1021/acs.est.9b04210>
2. Wu, Q., Jia, Y., Liu, Q., et al., “Ultra-dense carbon defects as highly active sites for oxygen reduction catalysis,” *Chem* **2022**, 8, 2715-2733. <https://doi.org/10.1016/j.chempr.2022.06.013>
3. Jia, Y., Chen, J., & Yao, X., “Defect electrocatalytic mechanism: concept, topological structure and perspective,” *Mater. Chem. Front.* **2018**, 2, 1250-1268. <https://doi.org/10.1039/C8QM00070K>
4. Xu, J., Avellan, A., Li, H., et al., “Sulfur loading and speciation control the hydrophobicity, electron transfer, reactivity, and selectivity of sulfidized nanoscale zerovalent iron,” *Adv. Mater.* **2020**, 32, 1906910. <https://doi.org/10.1002/adma.201906910>
5. Kresse, G., & Furthmüller, J., “Efficiency of ab-initio total energy calculations for metals and semiconductors using a plane-wave basis set,” *Comp. Mater. Sci.* **1996**, 6, 15–50. <https://doi.org/10.1016/0927-0256(96)00008-0>
6. Blo ̈chl, P.E., Jepsen, O., & Andersen, O. K., “Improved tetrahedron method for brillouin- zone integrations,” *Phys. Rev. B.* **1994**, 49, 16223–16233. <https://doi.org/10.1103/PhysRevB.49.16223>
7. Perdew, J. P., Chevary, J. A., Vosko, S. H., et al., “Erratum: atoms, molecules, solids, and surfaces: applications of the generalized gradient approximation for exchange and correlation,” *Phys. Rev. B.* **1993**, 48, 4978-4978. <https://doi.org/10.1103/PhysRevB.48.4978.2>
8. Grimme, S., Antony, J., Ehrlich, S., & Krieg, H., “A consistent and accurate ab initio parametrization of density functional dispersion correction (DFT-D) for the 94 elements H-Pu,” *The Journal of Chemical Physics* **2010**, 132, 154104. <https://doi.org/10.1063/1.3382344>
9. Dronskowski R., & Blöchl, P. E., “Crystal orbital Hamilton populations (COHP): energy-resolved visualization of chemical bonding in solids based on density-functional calculations,” *The Journal of Physical Chemistry* **1993**, 97, 8617-8624. <https://doi.org/10.1021/j100135a014>
10. Wei, K., Li, H., Gu, H., et al., “Strained zero-valent iron for highly efficient heavy metal removal,” *Adv. Funct. Mater.* **2022**, 32, 2200498. <https://doi.org/10.1002/adfm.202200498>
11. Wang, X., Chen, Y., Li, F., et al., “Site-selective protonation enables efficient carbon monoxide electroreduction to acetate,” *Nat. Commun.* **2024**, 15(1), 616. <https://doi.org/10.1038/s41467-024-44727-z>
12. Jin, X., Chen, H., Yang, Q., Hu, Y., & Yang, Z., “Dechlorination of Carbon Tetrachloride by Sulfide-Modified Nanoscale Zerovalent Iron,” *Environmental Engineering Science* **2018**, 35, 560–567. <https://doi.org/10.1089/ees.2016.0580>
13. Yu, F., Jia, C., Wu, X., et al., “Rapid self-heating synthesis of Fe- based nanomaterial catalyst for advanced oxidation,” *Nat. Commun.* **2023**, 14, 4975. <https://doi.org/10.1038/s41467-023-40691-2>
14. Li, Y., Lu, Z., Zheng, L., et al., “The synergistic catalysis effect on electrochemical nitrate reduction at the dual-function active sites of the heterostructure,”*Energy Environ. Sci.* **2024**, 17(13), 4582-4593. <https://doi.org/10.1039/D4EE00784K>
15. Lansford, J. L. & Vlachos, D. G., “Spectroscopic probe molecule selection using quantum theory, first-principles calculations, and machine learning,” *ACS Nano* **2020**, 14, 17295–17307. <https://doi.org/10.1021/acsnano.0c07408>
16. Liu Z., & Cheng Q., “Research on influencing factors of carbon information disclosure quality in China’s power industry,” *Environmental Science and Pollution Research* **2023**, 30, 20185-20202. <https://doi.org/10.1007/s11356-022-23554-z>
17. Bi, X., Cheng, Y., Dai, Q., et al., “Characteristics of the main primary source profiles of particulate matter across China from 1987 to 2017,” *Atmospheric Chemistry and Physics* **2019**, 19, 3223-3243. <https://doi.org/10.5194/acp-19-3223-2019>
18. Protocol M., “Montreal protocol on substances that deplete the ozone layer,” *Washington, DC: US Government Printing Office*, **1987**, 26: 128-136.
19. Nations U., “Kyoto protocol to the united nations framework convention on climate change,” **1998**.
20. Huo, Y. C., Li, W. W., Min, D., et al., “Zero-valent iron nanoparticles with sustained high reductive activity for carbon tetrachloride dechlorination,” *Rsc Advances* **2015**, 5(67), 54497-54504. <https://doi.org/10.1039/C5RA07052J>
21. Zhang, X., Deng, B., Guo, J., Wang, Y., & Lan, Y., “Ligand-assisted degradation of carbon tetrachloride by microscale zero-valent iron,” *Journal of environmental management* **2011**, 92(4), 1328-1333. <https://doi.org/10.1039/C5RA07052J>
22. Jin, X., Chen, H., Yang, Q., Hu, Y., & Yang, Z., “Dechlorination of carbon tetrachloride by sulfide-modified nanoscale zerovalent iron,” *Environmental Engineering Science* **2018**, 35(6), 560-567. <https://doi.org/10.1089/ees.2016.0580>
23. Lv, X., Prastistho, W., Yang, Q., & Tokoro, C., “Application of nano‐scale zero‐valent iron adsorbed on magnetite nanoparticles for removal of carbon tetrachloride: Products and degradation pathway,” *Applied Organometallic Chemistry* **2020**, 34(5), e5592. <https://doi.org/10.1002/aoc.5592>
24. Bae, Sungjun, and Woojin Lee., “Influence of riboflavin on nanoscale zero-valent iron reactivity during the degradation of carbon tetrachloride,” *Environ. Sci. Technol.* **2014**, 48(4), 2368-2376. <https://doi.org/10.1021/es4056565>
25. Ma, Y., Wang, Y., Lv, X., Meng, F., & Yang, Q., “Insight into the mode of action of Pd-doped zero-valent iron nanoparticles@ graphene (Pd/FePs@G) toward carbon tetrachloride dechlorination reaction in aqueous solution,” *Applied Catalysis A: General* **2018**, 560, 84-93. <https://doi.org/10.1016/j.apcata.2018.05.002>
26. Meng, Fansheng, Yiyang Ma, and Yeyao Wang., “Degradation of carbon tetrachloride using ultrasound‐assisted nanoscaled zero-valent iron particles@ sulfur/nitrogen dual-doped reduced graphene oxide composite: Kinetics, activation energy, effects of reaction conditions and degradation mechanism,” *Applied Organometallic Chemistry* **2019**, 33(8), e5014. <https://doi.org/10.1002/aoc.5014>
27. Li, Y., Zhang, Y., Yang, S., et al., “Citrate ligand-enhanced microscale zero-valent aluminum corrosion for carbon tetrachloride degradation with high electron utilization efficiency,” *Science of the Total Environment* **2021**, 783, 146999. <https://doi.org/10.1016/j.watres.2021.117328>
28. Zhang, Yanyan, Pinar Ozcer, and Subhasis Ghoshal., “A comprehensive assessment of the degradation of C1 and C2 chlorinated hydrocarbons by sulfidated nanoscale zerovalent iron,” *Water Res.* **2021**, 201, 117328. <https://doi.org/10.1016/j.watres.2021.117328>
29. Zhu, X., Zhou, L., Li, Y., Han, B., & Feng, Q., “Rapid degradation of carbon tetrachloride by microscale Ag/Fe bimetallic particles,” *International Journal of Environmental Research and Public Health* **2021**, 18(4), 2124. <https://doi.org/10.3390/ijerph18042124>
30. Huang, L. Z., Zhan, X., Chen, W., et al., “Catalytic dechlorination of carbon tetrachloride to combustible hydrocarbons by Pd-Fe hydroxides through atomic hydrogen attack and direct electron transfer,” *Separation and Purification Technology* **2024**, 346, 127449. <https://doi.org/10.1016/j.seppur.2024.127449>
31. Jia, Q. Q., Deng, J., Zhang, X., Dai, Y., Wu, F., & Huang, L. Z., “Hydroxyl groups bridge the electron transfer from Fe (II) to carbon tetrachloride,” *Water Res.* **2022**, 221, 118791. <https://doi.org/10.1016/j.watres.2022.118791>
32. Brumovský, M., Oborná, J., Micić, V., et al., “Iron nitride nanoparticles for enhanced reductive dechlorination of trichloroethylene,” *Environ. Sci. Technol.* **2022**, 56(7), 4425-4436. <https://doi.org/10.1021/acs.est.1c08282>
33. Liu X, Wu M, Zhao J., “Removal of trichloroethylene from water by bimetallic Ni/Fe nanoparticles,” *Water*, **2022**, 14(10): 1616. <https://doi.org/10.3390/w14101616>
34. Gu, Y., Wang, B., He, F., Bradley, M. J., & Tratnyek, P. G., “Mechanochemically sulfidated microscale zero valent iron: pathways, kinetics, mechanism, and efficiency of trichloroethylene dechlorination,” *Environ. Sci. Technol.* **2017**, 51(21), 12653-12662. <https://doi.org/10.1021/acs.est.7b03604>
35. Cai, S., Cao, Z., Yang, L., et al., “Cations facilitate sulfidation of zero-valent iron by elemental sulfur: Mechanism and dechlorination application,” *Water Res.* **2023**, 242, 120262. <https://doi.org/10.1016/j.watres.2023.120262>
36. Wang, B., Luo, Q., Pan, Y., et al., “Enhanced biogenic sulfidation of zero-valent iron in columns: Implications for promoting dechlorination in permeable reactive barriers,” *Environ. Sci. Technol.* **2023**, 57(49), 20951-20961. <https://doi.org/10.1021/acs.est.3c06976>
37. Hu, X., Chen, C., Chen, D., et al., “Lattice engineered nanoscale Fe0 for selective reductions,” *Nature Water*, **2024**, 2(1), 84-92. <https://doi.org/10.1038/s44221-023-00175-5>
38. Xu, J., Wang, Y., Weng, C., et al., “Reactivity, selectivity, and long-term performance of sulfidized nanoscale zerovalent iron with different properties,” *Environ. Sci. Technol.* **2019**, 53(10), 5936-5945. <https://doi.org/10.1021/acs.est.9b00511>
39. Gong, L., Qiu, X., Cheng, D., et al., “Coincorporation of N and S into zero-valent iron to enhance TCE dechlorination: kinetics, electron efficiency, and dechlorination capacity,” *Environ. Sci. Technol.* **2021**, 55(23), 16088-16098. <https://doi.org/10.1021/acs.est.1c03784>
40. Gong, L., Qiu, X., Tratnyek, P. G., Liu, C., & He, F., “FeN_x_ (C)-coated microscale zero-valent iron for fast and stable trichloroethylene dechlorination in both acidic and basic pH conditions,” *Environ. Sci. Technol.* **2021**, 55(8), 5393-5402. <https://doi.org/10.1021/acs.est.0c08176>
41. Mo, Y., Xu, J., & Zhu, L., “Molecular structure and sulfur content affect reductive dechlorination of chlorinated ethenes by sulfidized nanoscale zerovalent iron,” *Environ. Sci. Technol.* **2022**, 56(9), 5808-5819. <https://doi.org/10.1021/acs.est.2c00284>
42. Xu, J., Avellan, A., Li, H., et al., “Iron and sulfur precursors affect crystalline structure, speciation, and reactivity of sulfidized nanoscale zerovalent iron,” *Environ. Sci. Technol.* **2020**, 54(20), 13294-13303. <https://doi.org/10.1021/acs.est.0c03879>
